# Mitochondrial and Nuclear DNA Variants in Amyotrophic Lateral Sclerosis: Enrichment in the Mitochondrial Control Region and Sirtuin Pathway Genes in Spinal Cord Tissue

**DOI:** 10.3390/biom14040411

**Published:** 2024-03-28

**Authors:** Sharon Natasha Cox, Claudio Lo Giudice, Anna Lavecchia, Maria Luana Poeta, Matteo Chiara, Ernesto Picardi, Graziano Pesole

**Affiliations:** 1 Department of Biosciences, Biotechnology and Environment, University of Bari “Aldo Moro”, 70126 Bari, Italy; anna.lavecchia@uniba.it (A.L.); marialuana.poeta@uniba.it (M.L.P.); ernesto.picardi@uniba.it (E.P.); 2 Institute of Biomedical Technologies, National Research Council, 70126 Bari, Italy; claudio.logiudice@cnr.it; 3 Department of Biosciences, University of Milan, 20133 Milan, Italy; matteo.chiara@unimi.it; 4 Institute of Biomembranes, Bioenergetics and Molecular Biotechnology, National Research Council, 70126 Bari, Italy

**Keywords:** Amyotrophic Lateral Sclerosis, bioinformatic pipeline, mtDNA, nDNA, WES, WGS, variants, heteroplasmy

## Abstract

Amyotrophic Lateral Sclerosis (ALS) is a progressive disease with prevalent mitochondrial dysfunctions affecting both upper and lower motor neurons in the motor cortex, brainstem, and spinal cord. Despite mitochondria having their own genome (mtDNA), in humans, most mitochondrial genes are encoded by the nuclear genome (nDNA). Our study aimed to simultaneously screen for nDNA and mtDNA genomes to assess for specific variant enrichment in ALS compared to control tissues. Here, we analysed whole exome (WES) and whole genome (WGS) sequencing data from spinal cord tissues, respectively, of 6 and 12 human donors. A total of 31,257 and 301,241 variants in nuclear-encoded mitochondrial genes were identified from WES and WGS, respectively, while mtDNA reads accounted for 73 and 332 variants. Despite technical differences, both datasets consistently revealed a specific enrichment of variants in the mitochondrial Control Region (CR) and in several of these genes directly associated with mitochondrial dynamics or with Sirtuin pathway genes within ALS tissues. Overall, our data support the hypothesis of a variant burden in specific genes, highlighting potential actionable targets for therapeutic interventions in ALS.

## 1. Introduction

Amyotrophic lateral sclerosis (ALS) is a neurodegenerative disorder characterised by upper motor neuron death in the cerebral cortex and lower motor neurons in the brainstem and spinal cord. ALS leads to progressive paralysis, disability, and eventually death [1]. Despite the fact that there are five FDA approved treatments for the disease (tofersen, AMX0035, edaravone, riluzole and Dextromethorphan/quinidine) [2,3,4], they mainly address the management of the symptoms with limited improvement in survival. In this perspective, finding safe and effective drugs for ALS remains an unmet clinical need. ALS can be categorised into two main forms based on patterns of inheritance: sporadic ALS (sALS) and familial ALS (fALS). In fALS, which occurs in 10% of patients, there is a familial mode of inheritance that can be identified by the occurrence of ALS in other first-degree relatives. Genome sequencing of fALS subjects has led to the discovery of several genomic variants implicated in the pathogenesis of the disease [5]. Studies on twins have also uncovered genetic risk factors for sALS, with an estimated heritability rate of 61%. However, at present, only a fraction of these determinants have been identified [6]. Prior to 2014, only 22 genes were known to be associated with ALS, and mutations in these genes explain around two-thirds of all fALS and approximately 10% of sALS cases [7]. More recently, the application of Next Generation Sequencing technologies in the genetic profiling of ALS patients has enabled the rapid identification of several new ALS-related genes, significantly enhancing our understanding of the disease [8].

Mitochondrial dysfunction is a prevalent feature of many neurodegenerative diseases, including ALS [9,10]. Different mitochondrial impairments have been identified in both the central nervous system and the muscle tissue of in vitro and in vivo models, playing a crucial role in ALS. A plethora of studies have been conducted on ALS-derived tissues. Including post-mortem brain tissue [11,12] and body fluid specimens such as cerebrospinal fluid [13], plasma [14], and urine [15], they have indeed shown increased oxidative damage to proteins, lipids, and DNA and a decrease in antioxidant defences, including glutathione reductase, glutathione, and catalase. Impairment in mitochondrial respiration has been extensively demonstrated in *SOD*-mutated mouse models, as well as in ALS patients carrying *SOD1* mutations [16,17]. Moreover, metabolic dysregulation has been observed in the spinal cord of *SOD1*^G93A^ mice across all disease stages [18]. Alterations in mitochondrial trafficking and morphology have also been demonstrated in ALS *SOD1* mutant mice, as well as in the early stages of ALS patients [19]. In this perspective, preclinical and clinical studies [20] targeting mitochondria pave the way for a new and promising therapeutic intervention in ALS. Motor neurons depend on mitochondrial function to fulfil their energetic requirements. Mitochondrial damage and the specific accumulation of mutated proteins (i.e., TDP-43) cause motor neuron intraneuronal damage and death via calcium-mediated excitotoxicity, an increase in ROS generation, and intrinsic apoptotic pathway activation [10,21,22]. Mitochondrial function relies on both mitochondrial DNA (mtDNA) and nuclear DNA (nDNA), and mutations in either of these genomes can damage the mitochondrial respiratory chain and lead to mitochondrial dysfunction. mtDNA encodes 13 essential catalytic peptides of the oxidative phosphorylation complexes (I, III–V), 22 transfer RNAs (tRNAs), and 2 ribosomal RNAs (rRNAs). These molecules are necessary for translating the protein-coding genes encoded in mitochondria, as they adhere to the specificity of the mitochondrial genetic code. The human mtDNA possesses a single non-coding control region (CR) spanning 1.1 kb, a portion (∼650 bp) of which contains a unique three-stranded DNA loop structure, referred to as the D-loop [23,24]. Regulatory elements for mtDNA transcription and replication, such as the light-strand promoter (LSP) and the heavy-strand promoters (HSP), Conserved Sequence Blocks (CSB), and termination-associated sequences (TAS), are located within the CR [25,26]. The mammalian mitochondrial proteome contains proteins responsible for cell apoptosis, mitophagy, nucleotide biosynthesis, metabolism, iron and calcium regulation, and those ensuring mitochondrial homeostasis and function. Nearly 99% of these proteins originate from genes encoded by the nDNA. These proteins are synthesised by cytoplasmic ribosomes before being transported into the mitochondria [26]. For instance, proteins involved in apoptosis regulation, such as cytochrome c and members of the Bcl-2 family, are encoded by nDNA, are predominantly located within the mitochondria, and play a pivotal role in the activation or inhibition of programmed cell death pathways [27]. Additionally, proteins engaged in mitophagy are also encoded by nDNA genes (i.e., *PARK7, PINK1, TOMM*) and function in a highly coordinated manner to selectively remove damaged mitochondria, preserving cellular health [28]. Other genes involved in mitochondrial dynamics, such as *MFN1*, *MFN2* (*Mitofusins*), *OPA1* (*Optic Atrophy 1*), *DRP1* (*Dynamin-related protein 1*), *FIS1* (*Mitochondrial fission 1 protein*), and components of the MICOS Complex, are encoded by nDNA. These genes regulate critical aspects of mitochondrial function, including fusion, fission, and cristae formation, collectively contributing to the dynamic nature and structural integrity of mitochondria [29].

The SLC25 family of mitochondrial carriers, encoded by 53 distinct genes within the nuclear DNA (nDNA), facilitates the transport of a wide array of compounds—such as amino acids, carboxylic acids, fatty acids, cofactors, inorganic ions, and nucleotides—across the inner mitochondrial membrane. This transport is crucial as it provides essential building blocks necessary for numerous cellular processes [30]. Additionally, proteins responsible for managing ions, including iron (e.g., MFRN1, MFRN2) and calcium (e.g., VDAC1, VDAC2, VDAC3), play pivotal roles in various cellular processes and are part of the mitochondrial proteome [31,32]. These proteins oversee the levels and distribution of these ions, ensuring their optimal concentrations for proper cellular functioning. Collectively, a total of 1136 nDNA-encoded genes have been assigned to specific locations within distinct sub-compartments of the mitochondria. Additionally, these genes were assigned to 149 hierarchical ‘MitoPathways’, which were organised into seven main functional categories [33].

mtDNA inheritance is matrilinear, as the mitochondria from the sperm cell are eliminated by the egg shortly after fertilisation [34]. Mutations in mtDNA can be inherited maternally or can occur randomly during DNA replication or repair. Since every cell has a specific but large copy number (CN) of mtDNAs (mtDNA-CN), mutations can be observed in all (homoplasmy) or only in some copies (heteroplasmy). The ratio between mtDNA copies carrying mutations and mtDNA copies not carrying mutations is called the heteroplasmic fraction (HF). When HF exceeds a certain threshold, heteroplasmy may affect the expression and activity of the oxidative phosphorylation complexes and modulate the susceptibility to mitochondrial-based diseases. The number of mtDNA variants and HF are tissue- or cell-specific and tends to increase with age [35], and somatic heteroplasmic variants that arise during life could play a role in the development of diseases. Furthermore, mtDNA-CN, together with HF, may affect the cell’s overall function [36], but differently from HF, mtDNA-CN has been shown to negatively correlate with age [37]. Targeting mitochondrial dysfunction represents a promising clinical avenue for ALS since it has the potential to extend survival in preclinical models. However, the correct timing of the therapy appears to affect the improvement in survival, with the highest benefit for interventions given before disease onset [38]. Therefore, further insights are needed to elucidate early molecular mechanisms, genes, and variants involved in disease initiation and progression to guide and advise future therapeutic protocols. In this study, we applied a bioinformatics workflow for analysing whole exome/genome sequencing data across two distinct datasets to pinpoint variants potentially associated with ALS both in mtDNA- and nDNA-encoded mitochondrial genes. Since mtDNA heteroplasmy levels vary among cells and across tissues, we specifically focused on the spinal cord, which is known to exhibit dense clusters of abnormal mitochondria in ALS patients [39] accompanied by alterations in mitochondrial oxidative phosphorylation and represents the key location for disease activity [40,41]. We performed an unprecedented screening of this tissue to identify homoplasmic and somatic heteroplasmic variants acquired during life in the spinal cord of ALS patients, with the aim of determining whether this tissue hosts a specific pattern of variants compared to healthy tissues. Our analyses highlighted an increased accumulation of variants in the mitochondrial CR and in genes of the Sirtuin Signalling Pathway, in ALS patients, irrespective of the assay used (WES or WGS). These results not only support, at a molecular level, the beneficial effects of specific natural antioxidants, such as resveratrol, which act on this pathway in ALS preclinical models, but can also be exploited to identify other new potential therapeutic targets in ALS.

## 2. Materials and Methods

WES dataset. Frozen spinal cord samples from 3 male donors affected by sALS and 3 sex and ethnicity matched controls were collected. These samples were obtained from the NICHD Brain and Tissue Bank for Developmental Disorders at the University of Maryland and the Human Brain and Spinal Fluid Resource Center in Los Angeles, CA (Table 1, http://medschool.umaryland.edu/btbank/). DNA was purified using the DNeasy Blood and Tissue Kit (Qiagen, Hilden, Germany) according to the manufacturer’s instructions, quantified, and qualitatively checked on NanoDrop 2000c (ThermoFisher Scientific, Waltham, MA, USA). Exome capture was performed using the TruSeq Exome Enrichment Kit (Illumina, San Diego, CA, USA), according to the manufacturer’s instructions. Briefly, for each tissue, a DNA library, including inserts ranging in size from 200 to 400 bp approximately, was prepared using the TruSeq DNA Sample Prep kit (Illumina). Then, each library was hybridised with biotinylated probes targeting the exonic regions (about 200,000 exons, covering about 62 Mb of the human genome). After two steps of enrichment, sequencing was performed on an Illumina HiSeq 2000 machine. An average of 68 million 100 bp paired-end reads were obtained for every sample.

WGS dataset. We acquired WGS fastq files of spinal cord tissue samples from 8 human ALS donors and 4 control subjects (Table 1). This data was provided by the New York Genome Center (NYGC). WGS data can be requested through Target ALS’s website (https://www.targetals.org/resource/genomic-datasets/).

### 2.1. nDNA Alignment and nDNA Variant Calling

Quality control (QC) and variant calling were performed according to GATK best practices (available at https://software.broadinstitute.org/gatk/best-practices/; accessed 1 February 2021; software version: v4.2.0.0) for both WES and WGS [42]. Alignment of the paired-end reads to GRCh38 human reference build was performed by the Burrows Wheeler Aligner (BWA, version: 0.7.17), using the bwa-mem command [43]. Alignment files were converted from SAM to BAM format using the samtools view (Version: 1.12) [44]. BAM files were sorted and indexed. PCR duplicates were removed by sambamba (Version: 0.6.8) [45]. A recalibration of the quality scores was performed using the GATKs BaseRecalibrator ApplyBQSR. SNPs and indels were called by HaplotypeCaller in a joint genotyping mode (GenotypeGVCFs). Variant Quality Score Recalibration (VQSR) of genotypes was conducted separately on SNPs and Indels, using a truth sensitivity threshold of 99.0 for SNPs and 90.0 for indels, respectively.

The complete list of the 1136 nDNA nuclear-encoded mitochondrial genes according to the MitoCarta3.0 human inventory was retrieved (https://www.broadinstitute.org/mitocarta/mitocarta) [33]. The liftover tool [46] was used to convert the .bed files containing the genomic coordinates of the genes from the hg19 to the hg38 reference assembly of the human genome. Variants within 10 kbp upstream and downstream of nuclear-encoded mitochondrial genes were included in our analyses.

### 2.2. mtDNA Assembly and Variant Calling

Exome reads were initially aligned to the revised Cambridge Reference Sequence (rCRS with GenBank accession number NC_012920.1) of human mtDNA [47] using GSNAP (version 2021-02-22) [48] and, subsequently, against the GRCh38 reference assembly of the genome. Read mapping with equal quality to both the nDNA and mtDNA was excluded from further analysis to discard nuclear mitochondrial DNA sequences (NUMTs) [49]. The complete mapping procedure was automated by using an updated, previously published custom Python script, mapExome.py [50,51]. Conversion from SAM to BAM format was performed by samtools (Version: 1.12) [44]. Sambamba (Version: 0.6.8) was used to sort, index BAM files, and remove PCR duplicates [45]. BSQR was performed by the GATKs “base recalibrator” tool. Alignment metrics (Coverage Metrics, Mapping Metrics, Alignment Statistics) were obtained using Qualimap (v.2.2.2) [52]. The MToolBox was used to reconstruct mtDNA sequences from alignments in BAM format [51]; specifically, assembleMTgenome.py and mtVariantCaller.py were used for variant calling and heteroplasmy quantification, then bases with a mapping quality score less than 25 were removed. Only positions covered by at least 5 independent reads were considered; the consensus base was calculated using a minimum confidence level of 0.75. The VCF output.py utility was used to produce the VCF containing mtDNA variants, related heteroplasmy fraction and confidence interval. Variants with a heteroplasmic fraction (HF) of 0.95–1 were considered homoplasmic, while variants with an HF < 0.95 were considered heteroplasmic. Variants associated with homopolymeric tracts, as defined by Andrews et al. and others [47,53] (np 66–71, 300–316, 513–525, 5892, 3106–3107, 12,418–12,425, and 16,182–16,194), were disregarded from subsequent analyses. Additionally, we excluded variants from subsequent analysis if they lacked support from both forward and reverse DNA stands, as this could suggest sequencing artefacts or bias. Low-confidence variants with HF < 0.01 were also excluded, as these can often be hard to distinguish from technical errors [54]. A custom Python script was created to automate the data processing tasks (https://github.com/BioinfoUNIBA/MitoVarFilter; accessed on 1 September 2023). The VCFs were merged and normalised by bcftools (v.1.12) [55], and duplicated variants were dropped with vt [56]. Mitomap was used to assign mtDNA function locations (https://www.mitomap.org/foswiki/bin/view/MITOMAP/GenomeLoci; accessed on 1 September 2022) and functional units crucial for regulating mtDNA replication and transcription within the CR, such as LSP, HSP1, HSP2, CSB1, CSB2, CSB3, TAS1, TAS2, and the central domain, which were obtained from Sbisà et al. [25]

### 2.3. nDNA and mtDNA Variant Annotation and Filtering

VCF files were annotated by SnpEff (http://snpeff.sourceforge.net/download.htm; version 5.0) [57]. Variants were subsequently categorised into 4 distinct classes according to SnpEff severity grades: HIGH (disruptive impact), including frameshift variants, stop-gain or -loss variants, splice donor or acceptor variants, and initiator codon variants; MODERATE, including missense variants and in-frame insertions and deletions; LOW (assumed to be mostly harmless or unlikely to change protein behaviour), stop retained variants, including synonymous variants, incomplete terminal codon variants, and splice-region variants; and 4) MODIFIER (usually non-coding variants or variants affecting non-coding genes, where predictions are difficult or there is no evidence of impact), including intronic and intergenic variants, 5′ and 3′ UTR variants, regulatory region and transcription factor binding site variants, miRNA variants, and non-coding exon variants. SnpSift was used to handle Variant Call Format (vcf) files and provide additional annotations from a selection of databases [58]. Both nDNA and mtDNA variants were further annotated with (i) the latest version of dbSNP153 and (ii) frequency data in the general population as reported in the largest genomic database, gnomAD (https://gnomad.broadinstitute.org/ version v3.1.2) (iii) variant-disease associations from the ClinVar database (ftp://ftp.ncbi.nih.gov/pub/clinvar/vcf_GRCh38/clinvar_20220205.vcf.gz; downloaded 01/05/2022). The effects of variants in mitochondrial tRNA genes were annotated by PON-mt-tRNA and MitoTIP [59,60]. Coding mtDNA variants were assessed by MitImpact 3D [61], which incorporates PolyPhen [62], SIFT [63], and APOGEE 2 [64]); mtoolnote (https://github.com/mitoNGS/mtoolnote; accessed on 1 September 2023), which contains scores from MutPred [65], Panther [66], PhDSNP [67], SNPsGO [68], Polyphen2 HDIV, and Polyphen2 HVAR [69]; and HmtDB_Pathogenicity (score retrieved from https://mseqdr.org/mvtool.php accessed on 1 September 2023). The deleteriousness of nDNA variants were scored using CADD [70], FAVOR [71] (containing SIFT, PolyPhen, Polyphen2 HDIV, Polyphen2 HVAR, MutationAssessor, MetaSVM Score), and FATHMM_X [72].

### 2.4. Disease Association Analysis

The most recent version of DIsGeNET (http://www.disgenet.org, v.7.0, accessed on 1 September 2023), a database that consolidates human gene-disease associations (GDAs) from expert-curated databases and text-mining-derived associations, encompassing Mendelian, complex, and environmental diseases [73,74], was used to perform disease association analysis. The analysis involved evaluating gene-disease correlations and assigning a final score based on evidence levels ranging from 0 to 1. This score integrates information from multiple types of evidence, including genetic association studies, gene expression studies, protein-protein interactions, text mining of biomedical literature, animal model data, and pathway analysis. A cutoff of 0.1 was selected. This score considers factors such as the number and quality of sources (including curation levels and model organisms) and the quantity of publications supporting each association.

### 2.5. Structural Predictions

Secondary structures were predicted by the RNAfold web server (http://rna.tbi.univie.ac.at/cgi-bin/RNAWebSuite/RNAfold.cgi; accessed on 1 September 2023) using default parameters to assess the impact of variants on mtDNA CR single strands. The minimum free energy prediction and base pair probabilities were utilised to assess their structural and possibly functional impact.

### 2.6. Network Analysis of Genes Containing Candidate Variants

A network analysis of genes containing candidate nDNA and mtDNA variants was performed to provide valuable insights into the interactions and functional relationships among these genes. nDNA variants were first scored using the above-mentioned pathogenicity scoring systems. Notably, as these pathogenicity scoring systems can give discordant classifications [75,76], we simplified the scores by assigning a categorical value to each score for each variant. For every variant, we condensed the scores that indicated harmful effects (such as possibly damaging, probably damaging, deleterious, pathogenic, medium deleterious, and high deleterious) into a single categorical value of deleterious (D). In the same way, we simplified the scores that indicated a harmless effect (such as tolerated, benign, neutral, and likely benign) into a single categorical value of benign (B). We counted the occurrences of ‘B’ and ‘D’ classifications for each variant. If a variant was predicted as ‘D’ by at least 4 pathogenicity predictors, we assigned it a concordance score of +2. We assigned a concordance score of +1 when the prediction “D” was consistent across up to 3 pathogenicity predictors. We assigned a concordance score of 0 if it was not predicted by any pathogenicity predictors or if there were conflicting interpretations of pathogenicity. A concordance score of −1 was assigned if classified as “B” in up to 3 pathogenicity predictors and −2 when at least 4 pathogenicity predictors classified the variants as “B” in. For mtDNA variant scoring, we simplified 12 pathogenic scoring systems (Hmtdb, ClinVar, MutPred, Panther, PhDSNP, SNPsGO, Polyphen2 HDIV, Polyphen2 HVAR, PON-mt-tRNA, MitoTIP, SIFT, and APOGEE2) in the same way. This method was used to visually represent genes within the network. Various colours were used to distinguish genes containing concordant harmful variants (displayed in red), non-harmful variants (shown in green), and variants with conflicting or no assigned pathogenicity predictions (denoted in grey). STRING (https://string-db.org; version 11.576) was used to evaluate protein-protein interaction (PPI) between genes containing variants. Interaction Networks were constructed using a cut-off confidence score of 0.6 with the STRINGApp Cytoscape extension (Version: 3.10.1) [77,78]. The Cytoscape plug-in Molecular Complex Detection (MCODE, http://apps.cytoscape.org/apps/mcode, version 2.0.0) was used to identify the most important sub-modules of the network map [79]. The following criteria were applied: cut-off = 2, MCODE score > 6, max depth = 100, node score cut-off = 0.2, and k-score = 2. The Biological Networks Gene Ontology tool (BiNGO, http://apps.cytoscape.org/apps/bingo; version 3.0.3) was used to analyse and visualise the biological processes of identified sub-modules, and a Benjamini-Hochberg FDR-corrected *p*-value < 0.001 was considered statistically significant. We used the Ingenuity Pathway Analysis software (IPA, Ingenuity System, Redwood City, CA, USA, version 107193442) to identify the most significant pathways from the IPA library of canonical pathways for our input data set. IPA calculates the significance of the pathways using the right-tailed Fisher’s Exact Test, and to account for multiple canonical pathways tested by IPA, the Benjamini-Hochberg FDR option was used (FDR < 0.05).

### 2.7. mtDNA-CN Estimation

mtDNA copy number (mtDNA-CN) was determined for both datasets. mtDNA-CN was estimated by applying the fastMitoCalc software (v1.0) [80]. The estimated mitochondrial copy number is twice the ratio of the average mitochondrial sequencing depth to the average autosomal sequencing depth. The average coverage of mtDNA and autosomal is obtained using sequence alignment with SAMtools [44].

### 2.8. Statistical Analyses

All data were tested for normality using the Shapiro–Wilk test. Normally distributed variables were expressed as mean ± standard deviation (SD) unless otherwise stated. For normally distributed variables, the two-tailed Student’s *t*-test was used to assess differences between two groups. Qualitative variables were summarised as counts and percentages, and comparisons between independent groups were performed by χ^2^ or Fisher’s exact tests. A *p* value < 0.05 was considered statistically significant. Statistical analyses and graphs were generated with GraphPad Prism 8.0.2 (GraphPad Software, San Diego, CA, USA).

## 3. Results

**Figure 1:**
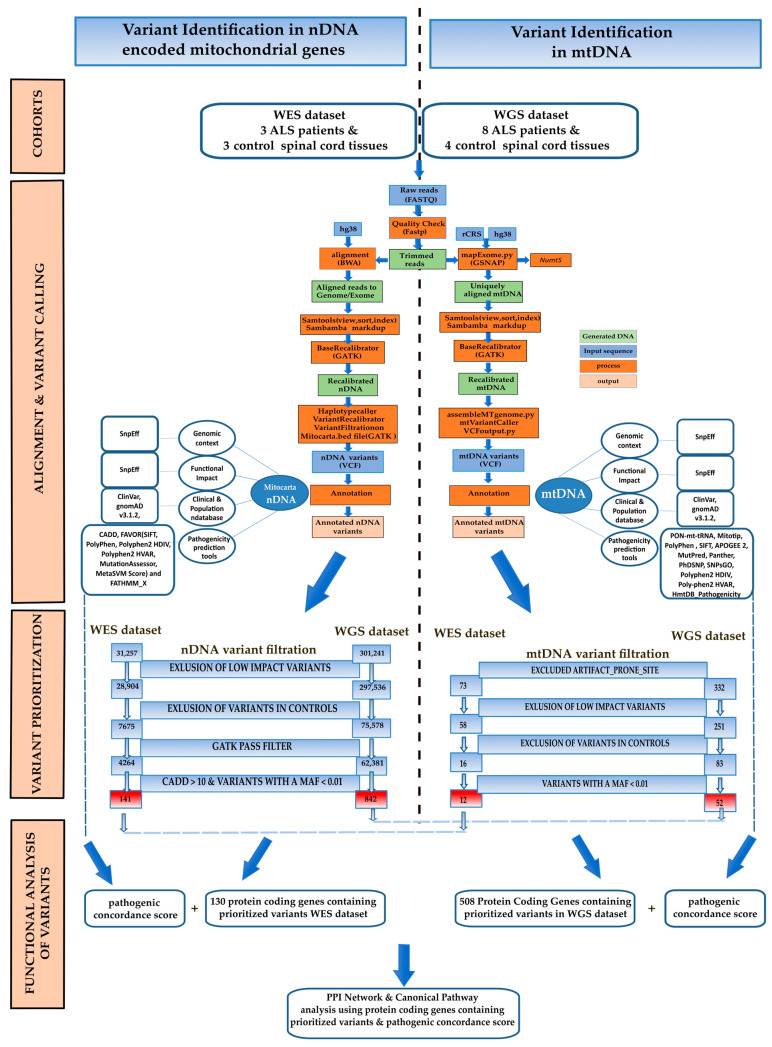
Overview of the analysis pipeline for identifying variants in nuclear DNA-encoded mitochondrial genes and mtDNA.

We applied the same pipeline for analysing whole WES and WGS sequencing data in two different datasets (Figure 1). The datasets were examined independently since they were obtained at different times and by different analytical protocols.

### 3.1. WES Dataset

#### 3.1.1. nDNA Alignment, Variant Calling Filtration, and Prioritisation

WES was performed on spinal cord samples from three sALS patients and three sex and ethnicity matched controls (Table 1). A total of 400 million 100 bp strand-specific paired-end reads were obtained; detailed statistics per sample are reported in Table S1; 99.72% of the reads were aligned to the reference human genome (GRCh38 assembly), with an average of 48 million pairs per sample. The target exome region showed an average depth of coverage at 53× and an average mapping quality of 58. On average, 86% were covered by at least 10 reads (Table S1). GATK Haplotypecaller identified a total of 664,642 variant sites in the subject’s nDNA, with an average of 241,416 variants per individual.

Of these, a total of 31,257 variants (average 11,976 variants per subject) were associated with nuclear-encoded mitochondrial genes, according to the MitoCarta3.0 human inventory [33] (Table S1). To focus on the variants that may affect the susceptibility to the disease, we excluded those with a LOW impact (N = 28,904) and those that were present in control samples. A total of N = 7675 variants were retained by these filters. We selected rare variants that had a MAF of less than 0.01, a CADD score of 10 or higher, and a GATK PASS filter. This selection process yielded a total of 171 variants specific to ALS patients. These potentially deleterious variants were identified at 141 distinct variant sites (Figure 1, Table S2), of which 32 were private variants and have never been reported in GnomAD or dbSNP. These variants were located within 128 genes or within 10 kb of them (Table S2). Of these, seven were in genes previously linked with ALS, according to the DisGeNET database (*DNM1L*, *SIRT3*, *ATP5F1A*, *OPA1*, *PARK7*, *HTRA2*, *IDI1*, Table S3). Our list includes *DNM1L*, also known as *Drp1*, which plays a significant role in cellular processes, particularly in mitochondrial dynamics [81]. Another gene found to be associated with ALS was *Sirtuin 3* (*SIRT3*). This gene functions as a mitochondrial deacetylase and maintains mitochondrial function and integrity. We found that two out of three patients carried the same rare missense variant c.785C>A (p.Pro262His; Table S2). This variant has a global MAF of 0.00981 according to GnomAD and was predicted to be deleterious by different pathogenicity predictors (SIFT, PolyPhen, Polyphen2-HDIV, Polyphen2-HVAR, MutationTaster) and a CADD score of 22 (a score that exceeds 20 indicates that the variant was predicted to be among the 1% most deleterious variants in the genome). Additionally, we investigated whether any of the genes identified by our analyses were listed in the ALS Online Genetic Database (ALSoD, http://alsod.iop.kcl.ac.uk/; accessed on 1 September 2023) [82]. Our findings indicated that only Parkinsonism Associated Deglycase (PARK7) and the 8-oxoguanine DNA glycosylase gene (OGG1) were previously identified as associated with ALS (Table 2 and Table S4). The *PARK7* (also known as *DJ-1*) gene is primarily expressed in the brain and spinal cord, responds to oxidative stress, and plays an important role in cellular defense mechanisms [83]. We observed a very rare g.1:7985265A>C transversion in the 3′ UTR region of the gene in two ALS patients. This mutation occurs within the experimentally validated target of miR-4639-5p, a miRNA known to regulate human *PARK7* gene expression [84]. Interestingly, in double transgenic *DJ-1 KO SOD1* mice, lack of *PARK7/DJ-1* genes led to accelerated damage within the CNS, accelerated disease progression, and reduced survival time, suggesting a protective role in the ALS disease model [83]. We found this mutation did not affect the perfect complementarity in the seed region (nucleotides 217–224 within the human *DJ-1* 3′UTR), but introduced an additional C-G pairing downstream using the RNAhybrid tool (Figure S1) [85]. This observation suggests an alteration of the miR-4639-5p regulatory pathway, potentially leading to an increased degradation of the target mRNA [86]. *OGG1*, another gene previously linked to ALS and listed in ALSoD (Table 2 and Table S4), carried a missense c.461G>A (p.Arg154His) variant in two out of the three ALS patients. This variant was predicted to be deleterious by all the methods for the evaluation of non-synonymous variants herein considered and had a very high CADD score of 31 (Table S2). *OGG1* encodes for a DNA glycosylase that specifically removes oxidised guanine (8-OHdG) from the DNA. Observations in the spinal motor neurons of sALS support a potential role for *OGG1* in ALS, since higher levels of 8-OHdG and lower mitochondrial OGG1 activity have been previously reported in sALS patients compared to healthy controls. This suggests that mtDNA may undergo oxidative damage and that the DNA repair mechanisms of *OGG1* may be compromised in ALS [87]. Finally, two ALS patients had a highly rare frameshift variant (c.313_314insA, p.Phe105fs; MAF: 1.315 × 10^−5^) caused by a single base insertion in exon 3 of the *Sideroflexin-2* (*SFXN2*) gene (Table S2). While *SFXN2* has not been previously linked to ALS and is not reported in DisGeNET or ALSoD (Table 2 and Table S4), its paralog, *SFXN3* gene, another member of the SFXN protein family, has been associated with neurodegenerative diseases [88].

#### 3.1.2. mtDNA Alignment, Variant Calling Filtration, and Proritisation

**Figure 2:**
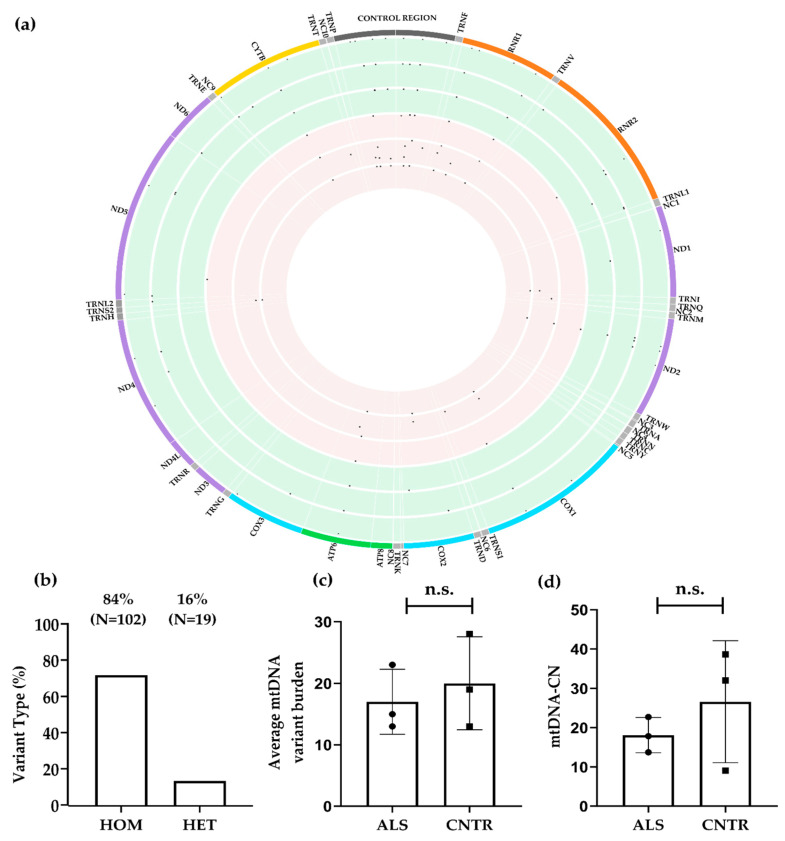
Descriptive statistics of mtDNA variants and mitochondrial DNA copy number (mtDNA-CN) in the WES dataset. (**a**). Circos plot showing the frequency of 121 mtDNA variants (HF ≥ 0.01) at 73 variable sites in the mtDNA. ALS subjects are represented in red (N = 3), and controls (N = 3) in green. Dots represent variants; relative height within each circle indicates the levels of heteroplasmy (≤95%) or homoplasmy (>95%). Coloured boxes represent functional regulatory elements and genes: control region (dark grey), transfer RNAs (light gray), ribosomal RNAs (orange), Complex I NADH dehydrogenase genes (purple), Complex III cytochrome c reductase gene (yellow), Complex IV cytochrome c oxidase genes (blue), and Complex V adenosine triphosphate synthase genes (green). (**b**) Percentage of homoplasmic (84%) and heteroplasmic variants (16%). (**c**) Overall mtDNA variant burden between cases (17.0 ± 5.92) and control tissues (20.0 ± 7.55). (**d**) mtDNA-CN in three ALS tissues (18.1 ± 4.5) and three control tissues (26.6 ± 15.51). n.s. = not statistically significant.

We used a previously published computational strategy to assemble the complete mitochondrial genome from off-target WES reads. First, we assessed the quality and coverage of the reconstructed mitochondrial genomes. After the removal of probable contaminating NUMTs, we uniquely identified a mean of 17,814 bona fide mitochondrial reads per sample (Table S1). A mean of 17% of the reads per sample were removed due to duplicates. On average, 107× of the mtDNA coverage was attained with an average mapping quality of 40 (Table S1). A high coverage level of 30× or higher of the complete mtDNA sequence was recovered for all the samples. Each sample exhibited 100% coverage of mtDNA with a minimum of 30 reads, except for CNTR_1, which displayed a lower coverage (100% mtDNA sequence covered by 5 or more reads). Cumulatively, a total of 121 variants at 73 distinct sites in the mtDNA were identified in the six samples (Figure 2a), including 72 Single Nucleotide Polymorphisms (SNPs) and one indel; with a mean of 17 homoplasmic and 3 heteroplasmic variants per sample (Table S1).

Of the 121 variations identified, 84% (N = 102) were homoplasmic and 16% (N = 19) were heteroplasmic (Figure 2b) and the overall variant burden did not differ significantly between cases (17.0 ± 5.92) and controls (20.0 ± 7.55, *p* = 0.603 *t*-test, Figure 2c). However, ALS samples demonstrated a statistically significant increase in the number of heteroplasmic variants compared to controls (*p* < 0.0001; Fisher’s exact test, Figure S2a, Table 3).

The proportion of variants in the coding and CR of the mitochondrial genome were similar in cases and controls. However, the variant load in the CR was higher in ALS cases (35% vs. 24%, Table 3), but the difference did not reach statistical significance (*p* = 0.225; Fisher’s exact test, Figure S2b, Table 3). The CR includes ‘hypervariable’ segments exhibiting a significant rate of variation among different ethnicities [89]. A specific analysis of the CR shows an increased number of variants within the Hyper-Variable regions (HV) 1 and 2 (Figure S2c, Table 3) and a significantly higher proportion of heteroplasmic mutations in cases (*p* = 0.0001; Fisher’s exact test, Figure S2d). Examining the control elements within the CR, such as CSB, ETAS, and LSP, we identified four variants across three ETAS sites in all three patients (m.16298T>C; m.16304T>C; m.16311T>C). Notably, these variants were exclusively observed in ALS cases (Table S5). Secondary structure predictions were performed to assess the potential impact on the stability of mitochondrial single-stranded (ss) mtDNA secondary structures. By considering the first 42 nt of the ETAS2 region as identified by Sbisà et al. [25], this region includes structure C, one of the 13 CR secondary structures (A-M) described by Pereira et al. [24]. In ALS_1, we detected a m.16298T>C variant, which however had limited structural or thermodynamic impact on secondary structure stability (Figure S3a,b); in ALS_2, we detected two variants in close proximity, m.16304T>C andm.16311T>C (Figure S3c); and ALS_3 only carried the m.16311T>C variant, which has already been reported as a potential risk factor for stroke [90]. These variants were predicted to induce a marked conformational change of the secondary structure C (Figure S3d), thereby reducing the stability of the ETAS2 region.

The examination of protein-coding genes shows a marginal and not statistically significant (*p* = 0.48; chi-square test, Table 3) over-representation in the number of variants in the *Mitochondrially Encoded Cytochrome C Oxidase I* (*MT-CO1*) and the *Mitochondrially Encoded ATP Synthase Membrane Subunit 6* (*MT-ATP6*) genes in ALS patients (Figure S2e). The proportion of heteroplasmy/homoplasmy in protein-coding genes did not differ significantly (*p* = 0.5059; Fisher’s exact test, Figure S2f) between ALS and controls, suggesting that the differences in heteroplasmy between cases and controls are limited exclusively to the D-loop region. Next, we assessed the number of mutations in tRNA and rRNA genes. Again, no statistically significant difference in the total number of variants was observed (*p* = 0.1052; chi-square test, Table 3; Figure S2g), However, ALS samples showed a statistically significant increase in the proportion of heteroplasmic variants compared to controls (*p* < 0.0001; Fisher’s exact test, Figure S2h).

All nucleotide substitutions were transitions, concordant with the extensively known high transition-biased nucleotide substitution patterns in mammalian mtDNA [91]. The difference in the distribution of the four types of transitions (A>G; C>T; G>A; T>C) between cases and controls did not reach statistical significance (*p* = 0.0527, chi-square test, Table 3).

We categorised variants according to their functional annotation (HIGH, MODERATE, LOW, and MODIFIER), but we did not observe any difference in the proportion between cases and controls (*p* = 0.2370; chi-square test, Figure S2i) or in the proportion of homoplasmic variants. However, the functional annotation distributions of heteroplasmic variants differed significantly between ALS compared to controls (*p* = 0.0001; chi-square test, Figure S2j), with a particular enrichment of low and modifier impact variants in patients. Comparing the identified variants with the dbSNP153 and gnomAD v3.1.2, we found that 72 out of 73 (98.63%) variants were previously reported.

Variant prioritisation was performed by excluding low-impact variants and those that were also called in control tissues, as well as variants with a MAF ≥ 0.01. A total of 16 rare variants at 12 variant sites passed these filters, none of these have been previously associated with ALS (Figure 1, Table S5). The prioritised variants were mostly heteroplasmic (75%, N = 12), except for m.1007G>A, m.4336T>C, m.7410C>T/H503Y, m8027G>A/A148T, 12235T>C, and 16240A>G, which were homoplasmic in some individuals and heteroplasmic in other individuals (Table S5). Of note, despite their rarity, four of these variants (m.1007G>A, m.8027G>A, m.12235T>C, and m.16240A>G) were detected in two out of the three ALS patients. Moreover, we detected a rare private heteroplasmic variant, m.7410C>T, in the MT-CO1 gene with a predicted damaging impact. Prioritised variants were preferentially localised in the CR (44%, N = 7) even though this region only accounts for 6.7% of total mtDNA length. Only one prioritised variant fell within the annotated CR functional elements. Specifically, m.16304T>C within ETAS2 Region [25]. The potential impact of prioritised variants on mtDNA secondary structures in the CR was investigated [24]. In ALS_1, m.195T>C and m198C>T (Table S5) appeared to induce a conformational rearrangement in the secondary structure element K (positions m.181–226), resulting in increased instability as compared to the wild-type (Figure S4a,b). Secondary structure K encompasses CSB1 and the H-strand replication site (O_H_). Furthermore, in ALS_2 m.152T>C, a rare variant is located close to (three nucleotides apart) the J secondary structure element (nt. 116–149) in the central domain. This variant was predicted to induce a major conformational rearrangement of the J secondary structure, resulting in increased instability as compared to the WT sequence (Figure S4c,d). The filtered variant m.16304T>C in ALS_2 has been previously documented in the ETAS2 region (Figure S3c).

### 3.2. WGS Dataset

#### 3.2.1. nDNA Alignment, Variant Calling Filtration, and Prioritisation

The same bioinformatics workflow used for WES was applied to the analysis of the WGS dataset. This dataset included a total of 11 billion 150 bp paired-end reads, with an average of 954M. A total of 301,241 variants were associated with MitoCarta3.0 genes, with an average of 107,510 variants per individual (Table S6). Variants with LOW impact and those shared by cases and controls were discarded (Figure 1). This reduced the number of variants to N = 75,578. We then selected rare variants with a MAF < 0.01, a CADD score > 10, and a GATK PASS filter. This selection yielded 880 rare, potentially deleterious variants at 842 sites in/or in the proximity of 506 nuclear-encoded mitochondrial genes (Table S7, Figure 1). The *SLC25A21* gene had the highest burden of mutations, with seven out of eight ALS subjects carrying at least one prioritised variant. None of these variants were associated with protein-coding exons (Table S7). *SLC25A21* has not been previously associated with ALS (Table 2 and Table S4). This gene encodes a mitochondrial inner membrane protein that plays a role in transporting dicarboxylates across the inner membranes of mitochondria by a counter-exchange mechanism [92]. Notably, two out of three ALS subjects in the WES dataset harboured rare, intronic variants with a CADD score > 15 in the same gene. Similarly, *DNAJC11* gene was enriched in prioritised variants in both the WES and WGS datasets (Table 2, Tables S2, S4 and S7). Also, this gene has never been linked to ALS. DNAJC11 protein plays an essential role in organising the mitochondrial inner membrane by associating with the MICOS complex and the mitochondrial outer membrane sorting assembly machinery (SAM). Mutations in this gene lead to motor neuron pathologies linked to cristae disorganisation [93]. Further, our variant prioritisation strategy identified some genes containing variants in both WES and WGS datasets i.e., *DNM1L*. Specifically, this gene harbours an intronic g.12:32705995T>C variant, which has been predicted to be pathogenic by FATHMM_XF. Additionally, this gene is associated with ALS, according to the DisGeNET database (Table S3). A total of 30 genes identified by our variant prioritisation analyses were associated with ALS, according to DisGeNET. Among these, *OPA1* gene is remarkable, since it carries at least two prioritised variants in both datasets (Table 2, Tables S2, S4 and S7). *OPA1* encodes a protein crucial in mitochondrial fusion and has been demonstrated to undergo deacetylation by SIRT3, which affects mitochondrial dynamics and maintenance [94].

An extremely rare (MAF = 6.58 × 10^−6^ according to gnomAD) variant with a high CADD score was also observed in the first intron of the *PARK7* gene (g.1:7962280G>A) (Table S7). Evidence of an involvement of PARK7 and PINK1 in mitochondrial dysfunction and muscle degeneration has been thoroughly investigated both in sALS patients and in the *SOD1*^G93A^ ALS mouse model [95]. Both genes contained variants in this dataset (Table S7).

#### 3.2.2. mtDNA Assembly, Variant Calling Filtration, and Prioritisation

**Figure 3:**
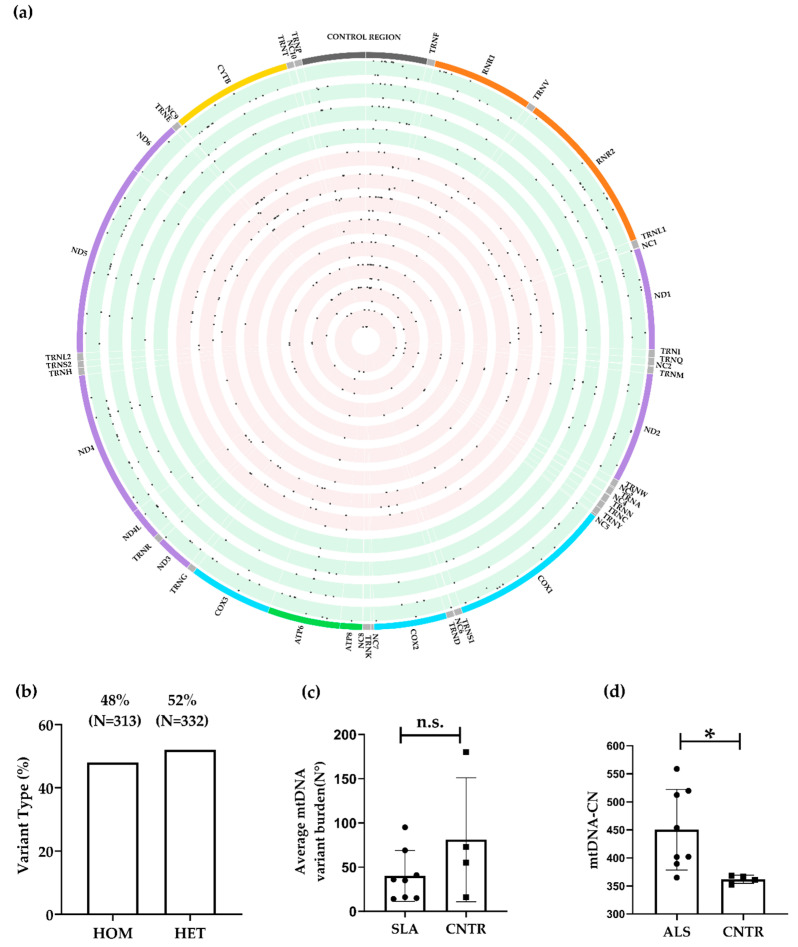
Descriptive statistics of mtDNA variants and mitochondrial DNA copy number (mtDNA-CN) in the WGS dataset. (**a**) Circos plot showing the frequency of 645 mtDNA variants (HF ≥ 0.01) within 332 variation sites across the entire mitochondrial genome. The red and green shaded circos plots represent cases (N = 8) and controls (N = 4), respectively. Dots represent variants; relative height within each circle indicates the levels of heteroplasmy (≤95%) or homoplasmy (>95%). Coloured boxes represent functional regulatory elements and genes: Control region (dark grey), transfer RNAs (light gray), ribosomal RNAs (orange), Complex I NADH dehydrogenase genes (purple), Complex III cytochrome c reductase gene (yellow), Complex IV cytochrome c oxidase genes (blue), and Complex V adenosine triphosphate synthase genes (green). (**b**) Pie chart displaying the percentage of homoplasmic (48%) and heteroplasmic variants (52%). (**c**) Overall mtDNA variant burden between cases (40.13 ± 28.75) and control tissues (81.0 ± 70.16). (**d**) mtDNA-CN in eight ALS tissues (450.3 ± 71.85) compared to four control tissues (362.0 ± 7.20). n.s. = not statistically significant; * statistically significant *p* < 0.05.

WGS data provided ultra-deep coverage of the mitogenome (mean 4,312,655 reads per sample, mean coverage depth = 25,725×, mean mapping quality = 39, Table S6). All samples had 100% mtDNA covered by at least 50 reads. When high-confidence variants were considered, we identified 645 variants within 332 variant sites (26 homoplasmic and 28 heteroplasmic variants per sample) in 12 spinal cord tissue samples (Figure 3a). Of the 645 variations identified, 48% (N = 313) were homoplasmic and 52% (N = 332) were heteroplasmic (Figure 3b). As expected, considering the higher levels of coverage that provided an increased resolution [53,96], a higher proportion of heteroplasmic variants was observed in the WGS compared to the WES dataset. Consistent with our observations on WES data, there was no significant difference in the overall variant burden between cases (40.13 ± 28.75) and controls (81.0 ± 70.16; *p* = 0.17; Figure 3c), nor in the number of homoplasmic or heteroplasmic variants between the two groups (*p* = 0.08, Fisher’s exact test, Figure S5a, Table 4). Variants in the coding and CR of the mtDNA showed a different proportion between cases and controls. Specifically, we observed a statistically significant increase (*p* = 0.0482, Fisher’s exact test, Figure S5b, Table 4) of variants within the CR among ALS cases compared to controls (23% vs. 17%). A closer examination of the CR revealed that ALS patients carried a slightly higher number of variants than controls in HV2, HV3 (Table 4), but differences were not statistically significant (*p* = 0.71, chi-square test, Figure S5c, Table 4). No statistically significant differences were observed in the proportion of heteroplasmy/homoplasmy (*p* = 0.3855, Fisher’s exact test, Figure S5d). Upon detailed analysis of the functional domains within the CR, we identified a higher proportion of variants in ALS cases compared to control tissues in the central domain (55% vs. 38%) and LSP (24% vs. 19%); however, this difference was not statistically significant (*p* = 0.3574, chi-square test, Table 4). An analysis of the protein coding region shows a slightly higher number of variants in *Mitochondrially Encoded Cytochrome C Oxidase II* (*MT-CO2*), *Mitochondrially Encoded ATP Synthase Membrane Subunit 8* (*MT-ATP8*), and *Mitochondrially Encoded Cytochrome B* (*MT-CYB*) in ALS subjects compared to controls, but differences in the number of variants (*p* = 0.6290, chi-square test, Figure S5e, Table 4) and proportions of heteroplasmic/homoplasmic variants were not significantly different between the two groups (*p* = 0.5684, Fisher’s exact test, Figure S5e,f). Mutation frequencies in the tRNA and rRNA genes were consistent with those observed in the WES dataset, with more variants in the 12S rRNA and tRNA genes (Figure S5g). However, no statistically significant difference in the number of variants was observed between ALS subjects and controls (*p* = 0.3456, chi-square test, Table 4) or in the proportion of homoplasmic/heteroplasmic mutations (*p* = 0.1193, Fisher’s exact test, Figure S5h). Most of the identified nucleotide substitutions were transitions in both ALS and control subjects [97]. A statistically significant increase of A>G substitutions was observed in ALS (30% vs. 17%, Fisher’s exact test; *p* = 0.002, Table 4).

This difference reflected in a higher proportion of homoplasmic A>G substitutions (51% vs. 32%; Fisher’s exact test; *p* = 0.002, Table 4) in all the regions of the mitogenome in ALS, while the percentage of heteroplasmic A>G substitutions remained similar (5% vs. 5%; Fisher’s exact test; *p* = 0.675, Table 4).

We categorised variants into four groups based on the predicted effect (HIGH, MODERATE, LOW, and MODIFIER) and compared their distribution between cases and controls. We found no significant differences in the overall proportion (*p* = 0.2331, chi-square test, Figure S5i), nor in the proportion of heteroplasmic variants (*p* = 0.8048, chi-square test) or homoplasmic variants (*p* = 0.1455, chi-square test, Figure S5j). However, we observed a higher number of homoplasmic moderate (N = 40 vs. N = 30) and modifier (N = 80 vs. N = 59) variants in ALS cases. Comparing the identified variants with the dbSNP153 and gnomAD v3.1.2, we found that 93.67% (N = 311) of variants were already included in databases of human genetic variation. We then proceeded with variant prioritisation, and excluded low impact variants, those that were also called in control tissues, and those with a MAF < 0.01 gnomAD v3.1.2, obtaining a list of 58 rare mtDNA variants in 52 variant sites in cases and not in controls (Figure 1, Table S8). As observed in the WES dataset, prioritised variants were prevalently heteroplasmic (79%, N = 46) and have never been associated with ALS. Of note, 16% (N = 9) of the variants prioritised in the mtDNA were in the CR, within or a few nucleotides away from the CR DNA secondary structures J (m.143G>A), K (m.183A>G, m.204T>C), L (m.247G>T), A (m.16084G>A), and F (m.16496G>A) predicted by Pereira et al. [24]. In silico conformational/structural stability analyses suggested that m.143G>A, m.183A>G and m.204T>C resulted in a considerably lower predicted minimum free energy and a consequent reduction in stability of the J and K elements, respectively (Figure S6a–k).

#### 3.2.3. mtDNA Copy Number

Conflicting results have been previously reported regarding mtDNA copy number in ALS patients [98,99,100] We estimated the mtDNA copy number (mtDNA-CN) in ALS and control tissues in our datasets. In the WES dataset, there was no significant difference in mtDNA-CN between three ALS tissues (18.1 ± 4.5) and three control tissues (26.6 ± 15.51, *p* = 0.413, Figure 2d). However, in the WGS dataset, mtDNA-CN was significantly higher in eight ALS tissues (450.3 ± 71.85) compared to four control tissues (362.0 ± 7.20, *p* = 0.0377, Figure 3d).

### 3.3. Functional Analysis of Genes Containing Variants

#### 3.3.1. Protein-Protein Interaction (PPI) Network Analysis

**Figure 4:**
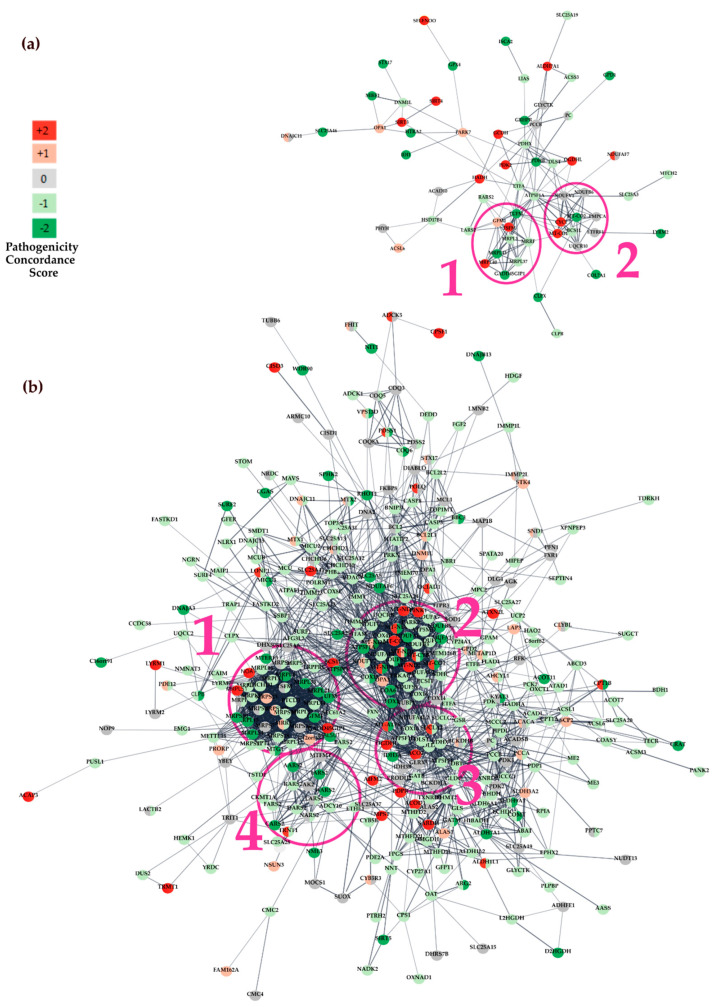
Protein-protein interaction (PPI) networks in genes containing prioritised variants. Red and green nodes represent the concordance score and show predicted deleterious and benign variants, respectively. We assigned a concordance score of +2 if predicted pathogenic by at least four pathogenicity predictors (red nodes); we assigned a concordance score of +1 if predicted pathogenic by up to three pathogenicity predictors (light red nodes). We assigned a concordance score of 0 if it was not predicted by any pathogenicity predictors or if there were conflicting interpretations of pathogenicity (grey nodes). A concordance score of -1 was assigned if classified as benign in up to three pathogenicity predictors (light green nodes) and -2 when all the predictors classified the variants as benign in at least four pathogenicity predictors (dark green nodes); edges are shown as grey. The most significant gene clusters within the network (MCODE score > 6) are marked in pink and numbered. (**a**) STRING Network constructed using 130 nDNA and mtDNA genes containing prioritised variants in the WES dataset. (**b**) STRING Network constructed using 508 nDNA and mtDNA protein-coding genes containing prioritised variants in the WGS dataset.

A network analysis was performed to detect functional interactions between genes containing prioritised variants. Protein-protein interaction (PPI) network analysis was constructed with STRING, which integrates both known and predicted PPIs and can be applied to predict functional interactions of proteins [78,101]. First, we considered nDNA and mtDNA coding genes containing variants that were prioritised in the WES dataset, and various colours were used to distinguish genes that were concordantly predicted to be harmful by pathogenicity scoring systems (displayed in red), concordantly predicted to be non-harmful variants (shown in green), and variants with conflicting or no assigned pathogenicity predictions (denoted in grey). The network was composed of 130 nodes and 167 interactions, with a minimum required interaction score > 0.6. Only query proteins were displayed (Figure 4a). The genes displaying strong interconnections within the network, suggesting functionally related clusters, were identified using MCODE [79].

The two most significant sub-modules of the network (MCODE score > 6) are highlighted in pink in Figure 4a. Functional enrichment analysis of Gene Ontology (GO) terms was performed with the BiNGO cystoscope plugin. The first sub-module defined by MCODE (subM1) contained nine highly interconnected genes and was significantly enriched in the following GO Biological Processes: *translation*, *cellular macromolecule biosynthetic process*, *macromolecule biosynthetic process*, *gene expression*, *cellular biosynthetic process*, *biosynthetic process*, *translational elongation*, *cellular protein metabolic process*, and *protein metabolic process* (FDR corrected *p*-value < 0.001; Table S9). While the second MCODE submodule (subM2), which included also two predicted deleterious variants in MT-CO1 and CYC1 (red shaded nodes), was significantly enriched in the *electron transport chain*, *ATP synthesis coupled electron transport*, *mitochondrial ATP synthesis coupled electron transport*, *respiratory electron transport chain*, *cellular respiration*, *oxidative phosphorylation*, *generation of precursor metabolites and energy*, *energy derivation by oxidation of organic compounds*, and *oxidation reduction* (FDR corrected *p*-value < 0.001; Table S9).

Subsequently, a protein interaction network was derived by considering genes that were prioritised in the WGS dataset. This network was composed of 508 nodes and 2444 interactions (Figure 4b) and contained four main submodules according to MCODE. The most interconnected submodule consisted of 32 genes and showed a coherent enrichment in GO Biological Processes with subM1 (*translation*, *cellular macromolecule biosynthetic process*, *gene expression*, *macromolecule biosynthetic process*, *cellular biosynthetic process*, *biosynthetic process*, *cellular protein metabolic process*, and *protein metabolic process*, Table S10). Interestingly, the second most significant submodule identified by MCODE in the PPI network based on WGS prioritised genes had GO terms functional enrichment patterns that closely mimicked those observed in subM2 (*electron transport chain*, *ATP synthesis coupled electron transport*, *mitochondrial ATP synthesis coupled electron transport*, *respiratory electron transport chain*, *cellular respiration*, *oxidative phosphorylation*, *generation of precursor metabolites and energy*, *energy derivation by oxidation of organic compounds*, and *oxidation reduction)*.

#### 3.3.2. Canonical Pathway Analysis with Ingenuity

**Figure 5:**
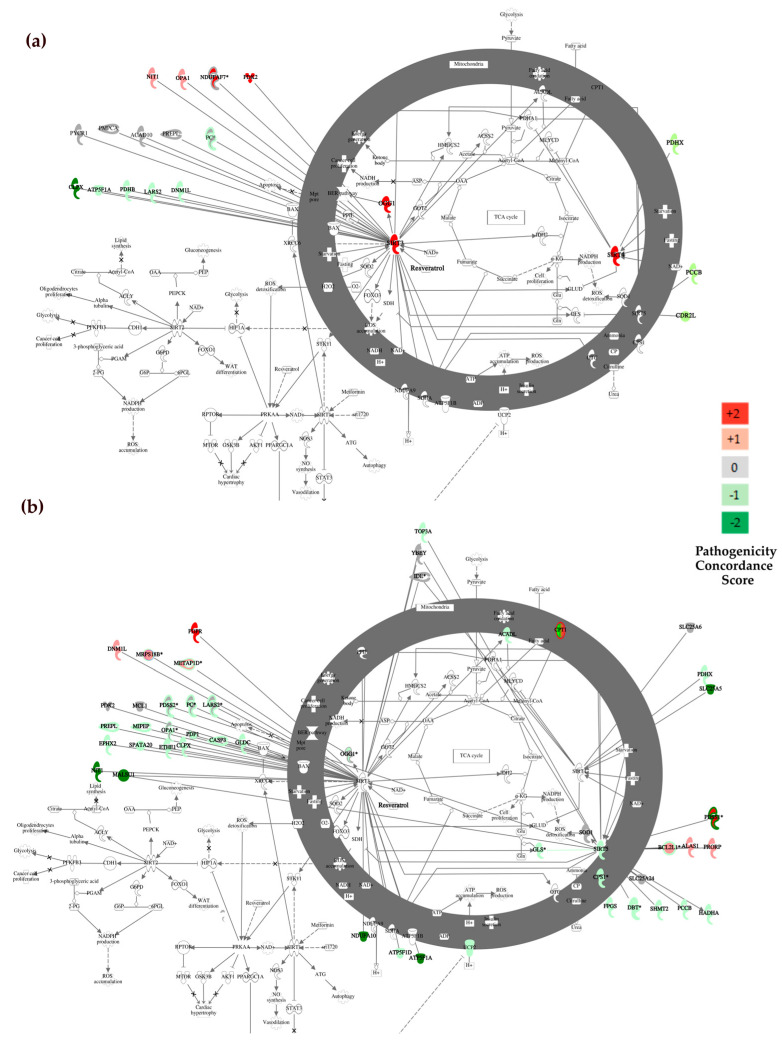
IPA canonical pathway analysis in genes containing prioritised variants. Red and green nodes represent the concordance score and show predicted deleterious and benign variants, respectively. We assigned a concordance score of +2 if predicted pathogenic by at least four pathogenicity predictors (red nodes); we assigned a concordance score of +1 if predicted pathogenic by up to three pathogenicity predictors (light red nodes). We assigned a concordance score of 0 if it was not predicted by any pathogenicity predictors or if there were conflicting interpretations of pathogenicity (grey nodes). A concordance score of -1 was assigned if classified as benign in up to three pathogenicity predictors (light green nodes) and -2 when all the predictors classified the variants as benign in at least four pathogenicity predictors (dark green nodes); edges are shown as grey. Sirtuin Signalling Pathway in the (**a**) WES dataset and in the (**b**) WGS dataset. Some genes containing variants were computed by IPA to be part of the Sirtuin Signalling Pathway other genes containing variants were not part of the pathway but were linked to it by direct interactions with the three mitochondrial sirtuins (SIRT3, SIRT4, and SIRT5). We used IPA’s “grow” functionality to add these genes to the pathway analysis. Some genes have been filled with more than one colour because they have different variants with different scores of pathogenicity concordance. * Genes containing more than one variant.

We performed a canonical pathway analysis with IPA by uploading genes containing variants along with their concordance score. As expected, strong and significant enrichments in mitochondrial-associated/related pathways were observed for both the WGS and WES gene sets. These included *mitochondrial dysfunction (p* = 1.01 × 10^−7^, *p* = 2.85 × 10^−32^, respectively, Table 5), *Oxidative Phosphorilation (p* = 9.72 × 10^−5^ and *p* = 1.98 × 10^−22^, respectively). We found that the *Sirtuin Signalling Pathway* was among the five most significant canonical pathways in both the WES (*p* = 5.83 × 10^−3^, Table 5, Figure 5a) and WGS (*p* = 1.98 × 10^−14^, Table 5, Figure 5b) datasets, meaning that genes containing variants were significantly enriched in this biological pathway. Within the Sirtuin pathway, deleterious mutations were identified in *SIRT3* and *OPA1* genes (Figure 5a red-shaded nodes). These genes play fundamental roles in mitochondrial function and regulation.

Mutations affecting these critical components could potentially disrupt mitochondrial dynamics and function, contributing to the pathology observed in ALS. In the Sirtuin pathway, the WGS dataset revealed mutations in genes that are associated with ALS pathology: *SOD1*, the gene most frequently presenting variants in ALS patients, and *SIRT5*, which modulates *SOD1* activity. The interaction between Sirtuin (SIRT5) and SOD1 proteins occurs in the mitochondria. In usual physiological conditions, SIRT5 activates SOD1, initiating the process of detoxifying reactive oxygen species [102]. Other variants have been found in *PDSS1* gene, also known as CoQ10, a component of the electron transport chain in mitochondria. Some studies have suggested a potential link between CoQ10 deficiency and mitochondrial dysfunction, supporting some clinical trials with CoQ10 supplements in ALS patients [103]. Interestingly, thirteen genes contain prioritised variants in both the WES and WGS datasets (*ATP5F1A, CLPX, DNM1L, LARS2, NIT1, OGG1, OPA1, OXPHOS, PC, PCCB, PDHX, PDK2,* and *PREPL*, Figure 5a,b) encoded proteins directly interact with the three mitochondrial sirtuins (SIRT3, SIRT4, and SIRT5), highlighting gene variants that were enriched in specific genes in ALS patients’ tissues. Resveratrol plays a central role in regulating the Sirtuin Signalling Pathway (Figure 5a,b) and may explain how this natural antioxidant exerts its beneficial effects in ALS preclinical models and patients [104,105].

## 4. Discussion

In this study, we simultaneously analysed mtDNA and nDNA variants affecting mitochondrial proteins. By harnessing whole exome/genome sequencing, we report, for the first time, an in-depth characterisation of somatic mtDNA and germline variants in spinal cord tissue samples obtained from ALS patients. Abnormal mitochondrial clusters and altered mitochondrial oxidative phosphorylation gene expression have been previously reported in ALS patients [40,106]. Therefore, we undertook a ground-breaking assessment of somatic heteroplasmic variants within this tissue, well-known for their implication in ALS activity. However, obtaining frozen spinal cord samples presents challenges and poses limitations for our study. Firstly, the lack of additional spinal cord samples impedes molecular analysis. Secondly, it explains the restricted sample size in this study: 6 samples for the WES dataset and 12 samples for the WGS dataset. They were acquired at distinct times, underwent different library preparation methods, and most importantly, derive from distinct spinal cord districts (lumbar for WES, and mixed lumbar/cervical for WGS). Furthermore, the WES dataset consisted of well-phenotyped sALS patients. In contrast, sequencing data from the WGS, obtained from ALS spinal cord tissues, were not classified as sporadic or familial due to the unavailability of family history, even if the lack of known ALS variants in *SOD1* gene would support a sporadic ALS.

An excess of heteroplasmic variants was identified in the WGS dataset (Figure 3b) compared to the WES dataset (Figure 2b). However, this discrepancy was mostly due to technical factors, specifically a higher coverage and sequencing depth, which enhanced the detection of heteroplasmic variants with lower frequencies [52,95]. Indeed, it must be emphasised that variant identification from WGS data is more reliable, unbiased, and exhibits higher sensitivity [107], whereas the efficiency of variant identification from WES data can vary significantly depending on the specific WES kit used. Interestingly, we observed a statistically significant increase in heteroplasmy levels in ALS cases compared to controls, but this difference was observed only in the WES dataset and not in the WGS dataset. We note that the difference in heteroplasmy in the WES dataset was primarily driven by ALS_2, which exhibited a higher number of heteroplasmic variants compared to all subjects in this dataset (Table S1). The high mtDNA-CN and the potential existence of multiple alternative alleles at a single DNA nucleotide position significantly complicate the task of detecting allelic variations in mtDNA. Moreover, additional challenges in variant calling with short-read sequencing data can be attributed to the presence of regulatory homopolymeric regions, such as CSB2 (nt.299–315), which plays a role in the formation of a hybrid G-quadruplex leading to premature termination [23]. Despite their functional importance, information on these areas is not available here or in most articles based on short read sequencing, as these regions are defined as “artifact_ prone_sites” [97]. However, these limitations could be overcome through the application of long-read sequencing techniques [108].

Moreover, we observed a consistent accumulation of variants in the mitochondrial CR in ALS patients across both datasets. This enrichment was statistically significant in the WGS dataset (23% vs. 17%, Table 4). In the WES dataset, although the variant load was higher in ALS cases in the CR (35% vs. 24%, Table 3), statistical significance was not achieved, possibly due to the limited number of cases. However, after variant prioritisation, we found that prioritised variants were also preferentially localised in the CR (44%, N = 7), in the WES dataset, and in the WGS dataset (16%, N = 9), despite this region comprising only 6.7% of the total mtDNA length. In addition to presenting a comprehensive list of mtDNA variants from both datasets, we have also explored the crucial aspect of how some rare prioritised variants characterising ALS patients might induce conformational changes in known secondary structure elements within the CR [24]. This region is particularly prone to the formation of these structures since extensive single-stranded DNA stretches are formed during mtDNA replication and transcription or during the formation of the three-stranded D-loop structure. CR variants may influence mtDNA molecular processes as this region contains the primary regulatory sequences for replication initiation and transcription [109]. Replication from the O_H_ is primed by transcription from the LSP by mitochondrial RNA polymerase (POLRMT), creating an intimate relationship between transcription and replication of mtDNA [110]. These molecular processes can be partially regulated by the formation of DNA secondary structures. Pereira et al. have identified 13 potential structures (A-M), most of which coincide with functional regulatory and conserved regions of the CR [24]. According to the classic strand displacement model, replication events initiated at the O_H_ are terminated in 95% of the cases, precisely after about 650 nt at the TAS regions, creating 7S DNA [111]. The secondary structures that are formed either at the 5′ end or the nascent 3′ end of the D-loop may serve as a recognition site for molecules that respectively prime or prematurely arrest H strand elongation [23,112]. Many speculate that the triple-stranded D-Loop serves as a switch between abortive and genome-wide mtDNA replication. The regulation of mtDNA replication is suggested to occur at the pre-termination level rather than initiation, acting as a molecular switch to control mtDNA-CN in response to cellular needs [24]. In both our datasets, we observed a higher proportion of variants within the CR in ALS cases compared to controls; statistically significant differences were observed in the WGS dataset. We further prioritised mtDNA variants based on their putative impact, frequency, and exclusive presence in ALS tissues. We observed that these prioritised variants in both datasets were predominantly heteroplasmic: 75% in the WES dataset and 79% in the WGS dataset. Since CR showed an enrichment of variants in ALS tissues, we focused our attention on prioritised variants within this region. Conformational analysis of mutated sequences indicated that secondary structures J and K showed an evident increase in predicted minimum free energy, suggesting increased instability as compared to the WT sequences. This trend was observed in both the WES dataset (Figure S4b,d) and the WGS dataset (Figure S6b,d,e), indicating lower stability of the mutated secondary structure. The importance of these foldable regions is dictated by the fact that K secondary structures contain O_H_ and CSB1, which are fundamental for both replication and transcriptional processes. Therefore, mutations occurring in proximity to these conserved motifs could potentially lead to changes in mtDNA replication and transcription. All three ALS tissues within the WES dataset carried variants in the conserved region of ETAS2, as identified by Sbisà et al. (nt 16294–16357). Specifically, the variants m.16298T>C, m.16304T>C, and m.16311T>C were detected. Notably, the latter two variants were predicted to induce a conformational change of the secondary structure C (Figure S3), thereby reducing its stability. These variants may impact both replication and transcriptional regulation, as the heavy-strand transcription termination was identified between positions nt. 16076–195 [26,113].

The analysis of nDNA variants affecting mitochondrial proteins revealed several genes previously linked to ALS, with the most involved in mitochondrial dynamics. Specifically, in both datasets, we identified *DNM1L/Drp1*, a gene playing a significant role in this cellular process. Previous studies have reported that inhibiting Drp1 function attenuates mitochondrial dysfunction and neurotoxicity in Parkinson’s Disease (PD) cell culture, providing preliminary evidence for future studies assessing the effect of blocking Drp1 in ALS [114]. Another gene associated with ALS and detected in both datasets is *OGG1*, which encodes a DNA glycosylase that removes 8-OHdG from the DNA. A role for OGG1 in ALS pathogenesis is supported by observations in spinal motor neurons of sALS, which exhibit higher levels of 8-OHdG and lower mitochondrial OGG1 activity compared to healthy controls [86]. A restoration of OGG1 has been proposed in high-oxidative stress conditions, including neurological diseases such as Alzheimer’s disease [115,116]. Interestingly, small molecule activators of OGG1 such as TH10785 have been demonstrated to increase repair of oxidative DNA lesions [117]. *OPA1* is another gene carrying at least two prioritised variants in both datasets. This gene encodes a protein crucial for mitochondrial fusion and affects mitochondrial dynamics and maintenance [94]. OPA1 levels are reduced in ALS animal models, leading to a fragmented mitochondrial network even before clinical symptoms manifest [118,119,120]. Some authors suggest that targeting OPA1 could be a potential therapeutic approach in ALS [121]. The different therapeutic options proposed to treat OPA1-associated neurodegenerative disorders include gene therapy and drugs potentially able to complement OPA1 defective function. Among them are antioxidants such as vitamins C, E, B2, B3, B12, lipoic acid, and folic acid, as well as drugs modifying mitochondrial biogenesis and mitophagy (i.e., bezafibrate, rosiglitazone, resveratrol) [122]. Various clinical trials assessing the effect of antioxidant compounds in ALS have been conducted or are still ongoing (i.e., NCT04244630, NCT02588807, and NCT04140136). Another important ALS-related gene identified by our analyses, as reported by DisGeNET or ALSoD, is the Parkinson disease-related *PARK7* gene. The co-occurrence of motor neuron disease and PD [123] implies the close link behind the aetiology of these two diseases. A case of parkinsonism-ALS-dementia complex has been found in an Italian family, in which three patients carried homozygous E163K mutations in *PARK7* exon 7 and a homozygous g.168_185dup mutation in the *PARK7* promoter [124]. Here, we detected a very rare g.1:7985265A>C transversion in the 3′ UTR region of the gene in two ALS patients. This mutation occurs within the target region of the human *PARK7* gene, which has been experimentally validated as a binding site for hsa-miR-4639-5p, a miRNA known to regulate human PARK7 expression. Another very rare variant was detected in the first intron of the *PARK7* gene (g.1:7962280G>A). Notably, one of the recently FDA-approved compounds, such as sodium phenylbutyrate (AMX0035), has demonstrated a neuroprotective effect in ALS and in both cellular and animal models of PD by up-regulating the PARK7/DJ-1 protein [125,126]. Furthermore, the effects of additional neuroprotective DJ-1 promoting compounds have been investigated in preclinical and clinical studies of PD [127].

Our analysis of nDNA variants also identified other genes containing variants that have never been associated with ALS (Table 2 and Table S4), and these were detected in both datasets. The *SLC25A21* gene showed the highest mutation burden, with variants in seven out of eight ALS subjects in the WGS dataset and in two out of three ALS subjects in the WES dataset. Despite no previous association with ALS, the *SLC25A21* gene encodes a mitochondrial inner membrane protein involved in transporting dicarboxylates within mitochondria [92]. Highlighting its significance in the context of ALS, a homozygous pathogenic c.695A>G; p.(Lys232Arg) variant within this gene led to mitochondrial dysfunction in a patient with a phenotype closely associated with ALS. This phenotype included toxicity in spinal motor neurons, ultimately leading to a disease resembling spinal muscular atrophy [128]. Future studies will need to assess the potential role of this gene in ALS. Additionally, *DNAJC11* emerged as a gene enriched with prioritised variants in both datasets. Importantly, this gene was not previously linked with ALS. DNAJC11’s organises the mitochondrial inner membrane through association with the MICOS complex and the mitochondrial outer membrane sorting assembly machinery (SAM). Mutations in DNAJC11 are associated with motor neuron pathologies linked to cristae disorganisation [93].

We then performed a PPI network analysis to explore potential protein-protein interactions between genes carrying prioritised variants. We considered both nDNA and mtDNA coding genes from both datasets independently. This analysis identified two significantly interconnected submodules. The first submodule, subM1, was enriched with Gene Ontology (GO) associated with translation, cellular macromolecule biosynthetic processes, gene expression, and other biosynthetic activities. Meanwhile, the second submodule, subM2, was enriched in genes related to the electron transport chain, ATP synthesis, and other mitochondrial processes. Notably, both datasets yielded very similar submodules in the analysis, providing consistent results. Canonical pathway analysis conducted with IPA identified the Sirtuin Signalling Pathway as one of the top five most significant canonical pathways associated with the sets of genes identified in both datasets, thereby suggesting a potential role of this pathway in ALS. This result, although potentially biased by the Mitocarta gene filtering, is in line with the reports that link Sirtuin alterations with ALS. Notably, ALS motor neurons exhibit hallmark metabolic defects that are rescued by SIRT3 activation, and a loss of SIRT3 or aberrant protein has been demonstrated to mimic ALS phenotypes [129]. Therefore, potential therapeutic strategies targeting this pathway, such as resveratrol, may exert beneficial effects, especially in the early stages of the disease, as demonstrated in mouse models of ALS [130,131]. Additionally, a Phase II study aims to assess the combination of liposome-delivered polyphenols resveratrol and curcumin with the drug G04CB02 in ALS patients (NCT04654689).

Growing evidence suggests that changes in epigenetic processes may contribute to ALS pathogenesis and its progression [132]. We also investigated mtDNA-CN, for which conflicting results have been reported so far in ALS patients [98,99,100]. We observed a slightly significant CN-mtDNA enrichment in ALS in the WGS dataset, for which the mtDNA-CN estimation is more reliable [133].

## 5. Conclusions

This study represents the first comprehensive report detailing a systematic screening revealing a consistent accumulation of somatic variant-specific genes and regions of mtDNA and mitochondrial genes encoded by nDNA in ALS spinal cord tissue. Several of these genes are directly associated with oxidative stress, mitochondrial dynamics, or the Sirtuin pathway genes. These results align with numerous studies on ALS animal models, indicating potential disruptions in these pathways [134,135,136,137]. Anomalous mitochondrial structure and cristae formation have been detected in both *SOD1* gene mutated mice and samples from ALS patients, particularly prevalent in the spinal cord [138]. Additionally, the Sirtuin pathway genes were significantly enriched in both datasets, supporting the beneficial effects of antioxidants such as resveratrol, which target this pathway, in ALS preclinical models and patients [104,105]. Others suggest OPA1 as a therapeutic target, which is known to cause mitochondrial ultrastructure alterations prior to the onset of clinical symptoms [121]. The findings here significantly show their translational relevance, emphasising their potential as targets for therapeutic interventions in ALS. Indeed, based on findings that some of the genes/pathways significantly dysregulated in ALS datasets are shared and already targeted in other neurodegenerative diseases such as Alzheimer’s and PD, this study provides useful evidence to guide drug repurposing strategies in ALS patients.

## Figures and Tables

**Table 1:** Amyotrophic lateral sclerosis (ALS) and Control (CNTR) samples.

	Origin	SAMPLE ID	Age	Sex	Tissue	Diagnosis
WES dataset	NICHD	SAMN02768876	61	Male	Spinal Cord Lumbar	ALS_1
	NICHD	SAMN02768878	54	Male	Spinal Cord Lumbar	ALS_2
	NICHD	SAMN02768879	59	Male	Spinal Cord Lumbar	ALS_3
	NICHD	SAMN02768883	56	Male	Spinal Cord Lumbar	CNTR_1
	NICHD	SAMN02768872	/	Male	Spinal Cord Lumbar	CNTR_2
	NICHD	SAMN02768875	/	Male	Spinal Cord Lumbar	CNTR_3
WGS dataset	NYGS	CGND-HDA-00497	60	Female	Spinal Cord Cervical	ALS1_497
	NYGS	CGND-HDA-00503	76	Male	Spinal Cord Cervical	ALS2_503
	NYGS	CGND-HDA-00536	58	Female	Spinal Cord Cervical	ALS3_536
	NYGS	CGND-HDA-00539	80	Male	Spinal Cord Cervical	ALS4_539
	NYGS	CGND-HDA-05608	/	Male	Spinal Cord Lumbar	ALS5_5608
	NYGS	CGND-HDA-05609	/	Male	Spinal Cord Lumbar	ALS6_5609
	NYGS	CGND-HDA-05610	/	Female	Spinal Cord Lumbar	ALS7_5610
	NYGS	CGND-HDA-05611	75	Female	Spinal Cord Lumbar	ALS8_5611
	NYGS	CGND-HDA-00596	65	Female	Spinal Cord Cervical	CNTR1_596
	NYGS	CGND-HDA-00597	50	Female	Spinal Cord Cervical	CNTR2_597
	NYGS	CGND-HDA-00598	16	Female	Spinal Cord Cervical	CNTR3_598
	NYGS	CGND-HDA-00599	61	Male	Spinal Cord Cervical	CNTR4_599

**Table 2:** Genes containing at least three variants that have passed variant prioritisation filters in Amyotrophic Lateral Sclerosis (ALS) patients in the Whole Exome Sequencing (WES) and Whole Genome Sequencing (WGS) datasets. The alphabetically ordered table can be found in Table S4. The full list of genes with prioritised variants can be found in Tables S2 and S7.

Official Gene Symbol	Gene Name	N° Variants WGS (WES)	WGS	WES	DisGeNET	ALSoD
*FHIT*	fragile histidine triad diadenosine triphosphatase	26 (0)	X			
*MSRA*	methionine sulfoxide reductase A	20 (0)	X			
*SLC25A21*	solute carrier family 25 member 21	15 (3)	X	X		
*CLYBL*	citramalyl-CoA lyase	11 (0)	X			
*IMMP2L*	inner mitochondrial membrane peptidase subunit 2	11 (0)	X			
*SND1*	staphylococcal nuclease and tudor domain containing 1	11 (0)	X			
*CHCHD3*	coiled-coil-helix-coiled-coil-helix domain containing 3	10 (0)	X			
*CHCHD6*	coiled-coil-helix-coiled-coil-helix domain containing 6	7 (0)	X			
*GPD2*	glycerol-3-phosphate dehydrogenase 2	7 (0)	X			
*OXR1*	oxidation resistance 1	7 (0)	X		X	
*SUGCT*	succinyl-CoA:glutarate-CoA transferase	7 (0)	X			
*AFG1L*	AFG1 like ATPase	6 (0)	X			
*BCKDHB*	branched chain keto acid dehydrogenase E1 subunit beta	6 (0)	X			
*DNAJC11*	DnaJ heat shock protein family	4 (2)	X	X		
*MRRF*	mitochondrial ribosome recycling factor	5 (1)	X	X		
*PCCA*	propionyl-CoA carboxylase subunit alpha	6 (0)	X			
*PDSS2*	decaprenyl diphosphate synthase subunit 2	6 (0)	X			
*VPS13D*	vacuolar protein sorting 13 homolog D	1 (5)	X	X		
*ETFA*	electron transfer flavoprotein subunit alpha	5 (1)	X	X		
*IDE*	insulin degrading enzyme	5 (0)	X			
*METAP1D*	methionyl aminopeptidase type 1D, mitochondrial	5 (0)	X			
*MRPS6*	mitochondrial ribosomal protein S6	5 (0)	X			
*MTX2*	metaxin 2	5 (0)	X			
*PC*	pyruvate carboxylase	2 (3)	X	X		
*PRELID2*	PRELI domain containing 2	1 (4)	X	X		
*SFXN5*	sideroflexin 5	5 (0)	X			
*SLC25A26*	solute carrier family 25 member 26	5 (0)	X			
*STX17*	syntaxin 17	3 (2)	X	X		
*VWA8*	von Willebrand factor A domain containing 8	4 (1)	X	X		
*ACACA*	acetyl-CoA carboxylase alpha	4 (0)	X			
*ACSM3*	acyl-CoA synthetase medium chain family member 3	2 (2)	X	X		
*BCL2*	BCL2 apoptosis regulator	4 (0)	X			
*GFER*	growth factor, augmenter of liver regeneration	2 (2)	X	X		
*HIBADH*	3-hydroxyisobutyrate dehydrogenase	4 (0)	X			
*METTL8*	methyltransferase 8, tRNA N3-cytidine	4 (0)	X			
*NARS2*	asparaginyl-tRNA synthetase 2, mitochondrial	4 (0)	X			
*NUBPL*	NUBP iron-sulfur cluster assembly factor, mitochondrial	4 (0)	X			
*OGG1*	8-oxoguanine DNA glycosylase	2 (2)	X	X		X
*OPA1*	OPA1 mitochondrial dynamin like GTPase	2 (2)	X	X	X	
*PDE2A*	phosphodiesterase 2A	4 (0)	X			
*SFXN2*	sideroflexin 2	0 (4)		X		
*SPHKAP*	SPHK1 interactor, AKAP domain containing	4 (0)	X			
*NDUFV2*	NADH:ubiquinone oxidoreductase core subunit V2	4 (0)	X			
*SLC25A35*	solute carrier family 25 member 35	4 (0)	X			
*TSFM*	Ts translation elongation factor, mitochondrial	2 (1)	X	X		
*ALDH1L1*	aldehyde dehydrogenase 1 family member L1	3 (0)	X			
*ARG2*	arginase 2	3 (0)	X			
*ATP5F1D*	ATP synthase F1 subunit delta	3 (0)	X			
*BCL2L1*	BCL2 like 1	3 (0)	X			
*CCDC51*	coiled-coil domain containing 51	3 (0)	X			
*CLPB*	ClpB family mitochondrial disaggregase	1 (2)	X	X		
*COMT*	catechol-O-methyltransferase	3 (0)	X			
*CRLS1*	cardiolipin synthase 1	2 (1)	X	X		
*CYB5R3*	cytochrome b5 reductase 3	3 (0)	X			
*DBT*	dihydrolipoamide branched chain transacylase E2	3 (0)	X			
*DELE1*	DAP3 binding cell death enhancer 1	2 (1)	X	X		
*DMGDH*	dimethylglycine dehydrogenase	3 (0)	X			
*DMPK*	DM1 protein kinase	1 (2)	X	X		
*GADD45GIP1*	GADD45G interacting protein 1	1 (2)	X	X		
*GATM*	glycine amidinotransferase	3 (0)	X			
*GLDC*	glycine decarboxylase	3 (0)	X			
*GLS*	glutaminase	3 (0)	X		X	
*LARS2*	leucyl-tRNA synthetase 2, mitochondrial	2 (1)	X	X		
*LYRM2*	LYR motif containing 2	1 (2)	X	X		
*MCU*	mitochondrial calcium uniporter	3 (0)	X		X	
*MICU2*	mitochondrial calcium uptake 2	3 (0)	X			
*MRPL1*	mitochondrial ribosomal protein L1	3 (0)	X			
*MRPS27*	mitochondrial ribosomal protein S27	3 (0)	X			
*MTHFD1L*	methylenetetrahydrofolate dehydrogenase	3 (0)	X			
*NBR1*	NBR1 autophagy cargo receptor	2 (1)	X	X		
*SHMT2*	serine hydroxymethyltransferase 2	4 (0)	X			
*NDUFS2*	NADH:ubiquinone oxidoreductase core subunit S2	3 (0)	X			
*NRDC*	nardilysin convertase	2 (1)	X	X		
*OCIAD1*	OCIA domain containing 1	2 (1)	X	X		
*OSBPL1A*	oxysterol binding protein like 1A	3 (0)	X			
*OXCT1*	3-oxoacid CoA-transferase 1	3 (0)	X			
*PARK7*	Parkinsonism associated deglycase	1 (2)	X	X	X	X
*PDHX*	pyruvate dehydrogenase complex component X	2 (1)	X	X		
*PDSS1*	decaprenyl diphosphate synthase subunit 1	3 (0)	X		X	
*PNKD*	PNKD metallo-beta-lactamase domain containing	3 (0)	X			
*PNPLA8*	patatin like phospholipase domain containing 8	3 (0)	X			
*POLQ*	DNA polymerase theta	2 (1)	X	X		
*PREPL*	prolyl endopeptidase like	1 (2)	X	X		
*RARS2*	arginyl-tRNA synthetase 2, mitochondrial	2 (1)	X	X		
*RTN4IP1*	reticulon 4 interacting protein 1	1 (2)	X	X		
*MTHFD2L*	methylenetetrahydrofolate dehydrogenase	5 (0)	X			X
*SLC25A3*	solute carrier family 25 member 3	0 (3)		X		
*SPIRE1*	spire type actin nucleation factor 1	3 (0)	X			
*SUOX*	sulfite oxidase	1 (2)	X	X		
*TMEM65*	transmembrane protein 65	2 (1)	X	X		

**Table 3:** Descriptive statistics of mtDNA variants found in the WES datasets.

	ALS	CNTR	*p* Value
Type of variant			0.0001 *
Het	17/51 (33%)	2/70 (3%)	
Hom	34/51 (67%)	68/70 (97%)	
Distribution of variants			0.2250 *
Control Region	18/51 (35%)	17/70 (24%)	
Coding Region	33/51 (65%)	53/70 (76%)	
CR sites			0.8061
CR HV1	6/18 (52%)	5/17 (29%)	
CR HV2	9/18 (48%)	7/17 (41%)	
CR HV3	1/18 (6%)	1/17 (6%)	
CR no-HV	2/18 (11%)	4/17 (24%)	
CR Functional Domains			
CENTRAL DOMAIN	3/7 (43%)	6/6 (100%)	
CSB	0/7 (0%)	0/6 (0%)	
ETAS	4/7 (57%)	0/6 (0%)	
LSP	0/7 (0%)	0/6 (0%)	
Coding regions			0.4854
MT-ND1	2/20 (10%)	2/36 (6%)	
MT-ND2	3/20 (15%)	7/36 (19%)	
MT-CO1	4/20 (20%)	3/36 (8%)	
MT-CO2	2/20 (10%)	2/36 (6%)	
MT-ATP6	4/20 (20%)	3/36 (8%)	
MT-CO3	0/20 (0%)	2/36 (6%)	
MT-ND4	0/20 (0%)	4/36 (11%)	
MT-ND5	1/20 (5%)	5/36 (14%)	
MT-ND6	0/20 (0%)	1/36 (3%)	
MT-CYB	4/20 (20%)	7/36 (19%)	
rRNA& tRNA genes			0.1052
12s rRNA	9/13 (69%)	8/17 (47%)	
16s rRNA	1/13 (8%)	7/17 (41%)	
tRNA	3/13 (23%)	1/17 (6%)	
Non-coding nt	0/13 (0%)	1/17 (6%)	
Type of substitution			0.0527
A>G	21/51 (41%)	34/69 (49%)	
C>T	6/51 (12%)	13/69 (19%)	
G>A	4/51 (8%)	10/69 (15%)	
T>C	20/51 (39%)	12/69 (17%)	

Data are expressed as n/N (%). *p* values were calculated using χ^2^ or Fisher’s exact test *; Het = heteroplasmic; Hom = homoplasmic.

**Table 4:** Descriptive statistics of mtDNA variants found in the WGS datasets.

	ALS	CNTR	*p* Value
Type of variant			0.0833 *
Het	154/321 (48%)	178/324 (55%)	
Hom	167/321 (52%)	146/324 (45%)	
Distribution of variants			0.0482 *
Control Region	74/321 (23%)	54/324 (17%)	
Coding Region	247/321 (77%)	270/324 (83%)	
CR sites			0.7145
CR HV1	18/74 (24%)	17/54 (29%)	
CR HV2	32/74 (43%)	23/54 (41%)	
CR HV3	8/74 (11%)	6/54 (6%)	
CR no-HV	16/74 (22%)	8/54 (24%)	
CR Functional Domains			0.3574
CENTRAL DOMAIN	16/29 (55%)	10/26 (38%)	
CSB	1/29 (3%)	1/26 (4%)	
ETAS	5/29 (17%)	10/26 (38%)	
LSP	7/29 (24%)	5/26 (19%)	
Coding regions			0.6290
MT-ND1	15/190 (8%)	14/211 (7%)	
MT-ND2	19/190 (10%)	27/211 (13%)	
MT-CO1	12/190 (6%)	21/211 (10%)	
MT-CO2	9/190 (5%)	6/211 (3%)	
MT-ATP8	2/190 (1%)	1/211 (0%)	
MT-ATP6	16/190 (8%)	19/211 (9%)	
MT-CO3	8/190 (4%)	11/211 (5%)	
MT-ND3	4/190 (2%)	7/211 (3%)	
MT-ND4L	6/190 (3%)	8/211 (4%)	
MT-ND4	25/190 (13%)	29/211 (14%)	
MT-ND5	28/190 (15%)	33/211 (16%)	
MT-ND6	6/190 (3%)	9/211 (4%)	
MT-CYB	40/190 (21%)	26/211 (12%)	
rRNA & tRNA genes			0.3456
12s rRNA	24/57 (42%)	21/59 (36%)	
16s rRNA	22/57 (39%)	28/59 (47%)	
tRNA	9/57 (16%)	5/59 (8%)	
Non-coding nt	2/57 (3%)	5/59 (8%)	
Type of substitution			0.002
A>G	92/303 (30%)	54/308 (17%)	
C>T	46/303 (15%)	64/308 (21%)	
G>A	129/303 (43%)	144/308 (46%)	
T>C	36/303 (12%)	46/308 (15%)	
Type of substitution (Het)			0.675
A>G	8/139 (5%)	8/166 (5%)	
C>T	21/139 (15%)	33/166 (20%)	
G>A	105/139 (76%)	121/166 (73%)	
T>C	5/139 (4%)	4/166 (2%)	
Type of substitution (Hom)			0.007
A>G	84/164 (51%)	46/142 (32%)	
C>T	25/164 (15%)	31/142 (22%)	
G>A	24/164 (15%)	23/142 (16%)	
T>C	31/164 (19%)	42/142 (30%)	

Data are expressed as n/N (%). *p*-values were calculated using χ^2^ or Fisher’s exact test *; Het = heteroplasmic; Hom = homoplasmic.

**Table 5:** Most representative pathways identified by IPA with genes containing variants in the ALS WES and WGS datasets.

Dataset	IPA Category	Pathway *p*-Value ^a^	Ratio ^b^	Gene Symbol
WES dataset	*Mitochondrial Dysfunction*	1.01 × 10^−7^	15/345	ATP5F1A, CYC1, MT-CO1, MT-CO2, DNM1L, GPX4, HTRA2, NDUFV1, NDUFB6, OPA1, PARK7, PDHX, PDHB, SIRT3, UQCR10
	*Tryptophan Degradation III*	9.83 × 10^−6^	5/23	GCDH, HADH, HSD17B4, KMO, PARK7
	*Glutaryl-CoA Degradation*	6.9 × 10^−5^	4/16	GCDH, HADH, HSD17B4, PARK7
	*Oxidative Phosphorylation*	9.72 × 10^−5^	7/112	ATP5F1A, CYC1, MT-CO1, MT-CO2, NDUFV1, NDUFB6, UQCR10,
	*Sirtuin signaling Pathway*	5.83 × 10^−3^	8/292	ATP5F1A, BCL2L11, CYC1, NDUFB6, NDUFV1, OGG1, SIRT3, SIRT4
WGS dataset	*Mitochondrial Dysfunction*	2.85 × 10^−32^	58/345	ACADL, ACO2, ADCY10, ARG2, ATP5F1A, ATP5F1D, ATP5PD, ATP5PO, ATPAF1, BBC3, BCL2, CACNA1G, CASP3, COX10, COX15, COX4I1, COX8C, CYB5B, CYB5R3, DLD, DNM1L, GPD2, GSR, ITPR3, MCU, MT-ATP6, MT-CO1, MT-CO2, MT-CYB, MT-ND1, MT-ND2, MT-ND4, MT-ND5, MT-ND6, MT-ND4L, NDUFA6, NDUFA7, NDUFA10, NDUFA4L2, NDUFB3, NDUFB5, NDUFS2, NDUFS5, NDUFV2, OPA1, PARK7, PDHX, PINK1, PRKACA, PRKN, RHOT1, SDHC, SOD1, SURF1, TFAM, UCP2, UQCRB, VDAC1
	*Oxidative Phosphorylation*	1.98 × 10^−22^	30/112	ATP5F1A, ATP5F1D, ATP5PD, ATP5PO, ATPAF1, COX10, COX15, COX4I1, COX8C, CYB5B, MT-ATP6, MT-CO1, MT-CO2, MT-CYB, MT-ND1, MT-ND2, MT-ND4, MT-ND5, MT-ND4L, NDUFA6, NDUFA7, NDUFA10, NDUFB3, NDUFB5, NDUFS2, NDUFS5, NDUFV2, SDHC, SURF1, UQCRB,
	*Sirtuin signaling Pathway*	1.98 × 10^−14^	35/292	ACADL, ARG2, ATP5F1A, ATP5F1D, CPS1, CPT1A, CPT1B, GLS, MT-ATP6, MT-CYB, MT-ND1, MT-ND2, MT-ND4, MT-ND5, MT-ND6, MT-ND4L, NDUFA6, NDUFA7, NDUFA10, NDUFA4L2, NDUFB3, NDUFB5, NDUFS2, NDUFS5, NDUFV2, OGG1, PCK2, SDHC, SIRT5, SLC25A5, SLC25A6, SOD1, TIMM23, UCP2, VDAC1
	*Granzyme A signalling*	4.22 × 10^−10^	7/112	LMNB2, MT-ND1, MT-ND2, MT-ND4, MT-ND5, MT-ND6, MT-ND4L, NDUFA6, NDUFA7, NDUFA10, NDUFA4L2, NDUFB3, NDUFB5, NDUFS2, NDUFS5, NDUFV2
	*Valine Degradation I*	1.04 × 10^−8^	9/20	ABAT, ACADSB, ALDH6A1, BCKDHA, BCKDHB, DBT, DLD, HADHA, HIBADH

^a^ Statistically significantly enriched pathway after Benjamini Hochberg correction for the False Discovery Rate (FDR < 0.05). ^b^ The ratio indicates the number of genes in the gene sets compared with the total number of genes in the pathway.

## Data Availability

WES data presented in this study are available in the publicly accessible repository dbGaP with Study Accession n° phs000747.v2.p1. WGS data can be requested through Target ALS’s website (https://www.targetals.org/resource/genomic-datasets/, accessed on 1 September 2022).

## Supplementary Materials

The following supporting information can be downloaded at: https://www.mdpi.com/article/10.3390/biom14040411/s1, Table S1: Statistics related to the Whole Exome Sequencing Dataset; Table S2: List of 141 prioritised nDNA variants in the WES cohort; Table S3: nDNA genes containing variants in the WES/WGS datasets associated with Amyotrophic Lateral Sclerosis as identified by DisGeNet; Table S4: Genes alphabetically ordered containing at least three variants that have passed variant prioritisation filters in Amyotrophic Lateral Sclerosis (ALS) patients in the Whole Exome Sequencing (WES) and Whole Genome Sequencing (WGS) datasets; Table S5: List of 12 prioritised mtDNA variant sites in the WES cohort; Table S6: Statistics related to the Whole Genome Sequencing Dataset; Table S7: List of 842 nDNA variants ranked by priority in the WGS dataset; Table S8: Fifty-two prioritised mtDNA variant sites in the WGS cohort; Table S9: Clusters of highly interconnected genes as identified by MCODE in the network generated with genes containing variants in the WES cohort; Table S10: Clusters of highly interconnected genes as identified by MCODE in the network generated with genes containing variants in the WGS cohort; Figure S1: RNA duplex formation predicted by the RNA hybrid of hsa-miR-4639-5p; Figure S2: mtDNA variant counts and proportions in WES dataset; Figure S3: Structural or thermodynamic differences between wild-type Cambridge Reference Sequence (rCRS) and mutated forms in ALS of the secondary structure C; Figure S4: Structural or thermodynamic differences of variant sites in mtDNA that passed the filtering and prioritisation steps in the WES dataset; Figure S5: mtDNA variant counts and proportions in the WGS dataset; Figure S6: Structural or thermodynamic differences of variant sites in mtDNA that passed the filtering and prioritisation steps in the WGS dataset.
