# Supplementary material for: Mitochondrial and Nuclear DNA Variants in Amyotrophic Lateral Sclerosis: Enrichment in the Mitochondrial Control Region and Sirtuin Pathway Genes in Spinal Cord Tissue
Source: Biomolecules. 2024 Mar 28;14(4):411. doi: 10.3390/biom14040411 (PMC11048214; doi:10.3390/biom14040411)

Table S1. Statistics related to the Whole Exome Sequencing Dataset

STATUS	ALS_1	ALS_2	ALS_3	CNTR_1	CNTR_2	CNTR_3	Mean
FASTQ FILES							
**read count r1**	21982097	74630914	24865095	26961680	22593307	26161247	32865723
**read count r2**	21982097	74630914	24865095	26961680	22593307	26161247	32865723
**total read count**	43964194	149261828	49730190	53923360	45186614	52322494	65731447
SEQUENCING & MAPPING DATA							
**n°Reads**	43943978	149198658	49709380	53861791	45166169	52300071	65696675
**% mapped reads to Reference Genome**	99.95	99.96	98.59	99.89	99.95	99.96	99.72
**properly paired reads**	43797642	148096362	49549044	50696974	44953150	52040418	64855598
**% properly paired**	99.62	99.22	99.64	94.02	99.48	99.46	98.6
EXOME CAPTURE (62Mbp) n°Reads							
**n°Reads to target region**	23775956	88493265	27227554	62051995	24682054	62051995	48047137
**% mapped reads to target region**	54.08	59.29	54.75	59.7	54.62	54.97	56.24
**mean coverage target region**	32.74	117.99	37.43	41.12	47.21	39.44	53
**% target region covered by 10 reads**	87	93	88	74	87	89	86
**mean mapping quality**	57.84	57.64	57.87	57.19	57.82	57.81	58
**duplicate reads**	611580	47483535	736284	24617761	693152	822361	12494112
**% duplicate reads**	2.57	53.66	2.7	76.47	2.81	2.86	24
**GC Percentage**	47.8	44.77	47.17	41.24	47.21	47.02	46
nDNA Variant Calling							
**n.**	228816	316724	231748	212826	224905	233475	241416
**Ts/Tv ratio**	2.22	2.16	2.21	2.20	2.21	2.20	2.2
nDNA Variant in Mitocarta genes							
**n.**	11625	14743	11697	10650	11154	11986	11976
MAPPING DATA to mtDNA							
***n°Reads**	18987	17686	17005	3956	29403	19844	17814
**% mapped reads to chrM**	100	100	100	100	100	100	100
**mean coverage target region**	115	106	103	20	178	119	**107**
**mean mapping quality**	39.32	39.84	39	39.94	39.3	39.38	40
**duplicate reads**	508	6794	464	7310	946	431	2742
**% duplicate reads**	3	28	3	65	3	2	17
**GC Percentage**	44.5	44.3	44.6	44.18	44.50	44.45	44
mtDNA Variant Calling							
**n.variants**	13	23	15	28	29	13	20
**homoplasmic**	13	6	15	25	29	13	17
**heteroplasmic**	0	17	0	3	0	0	3
**median depth**	77	82	68	14	127	76	74

Statistics were generated with Qualimap (v.2.2.2) on recalibrated bam file

Table S2. List of 141 Prioritized nDNA variant sites in the WES cohort

Variant Position	ID	N° Cases	GeneName	AF	SIFT/PolyPhen/FATHMM_score/CLNSIG/Polyphen2 HDIV/Polyphen2 HVAR/MutationTaster/Mutatio.ssessor/MetaSVM Score/CADD_PHRED Score	CLNDN
1:1468408G>A	rs182168962	ALS_1	ATAD3B	0.0021374	deleterious/benign/pathogenic/./0.515/0.117/1/3.04/T/22.6	.
1:6635588C>T	rs996736795	ALS_2	DNAJC11	0.000138	./../pathogenic/./././././NA/15.79	.
1:6667742TTC>T	rs374290353	ALS_2	DNAJC11	1.323E-05	./././././././NA/27.0	.
1:7985265A>C	rs746552212	ALS_2; ALS_3	PARK7	6.569E-05	./../pathogenic/./././././NA/11.76	.
1:12330118T>C	rs1286213511	ALS_3	VPS13D	6.573E-06	./../benign/./././././NA/12.15	.
1:51836567A>C	.	ALS_2	NRDC	.	./../benign/./././././NA/15.91	.
1:54200218G>A	rs199576695	ALS_2; ALS_3	MRPL37	0.0003809	./../benign/./././././NA/19.46	.
1:161121742A>G	rs998280227	ALS_3	NIT1	9.198E-05	./../pathogenic/./././././NA/22.1	.
1:212993539A>G	.	ALS_2	ANGEL2	.	./../benign/./././././NA/15.85	.
1:220791492T>G	rs771670966	ALS_2	MARC1	.	tolerated/probably_damaging/pathogenic/./0.876/0.713/0.918/2.66/T/26.3	.
1:241568656T>A	rs187112513	ALS_2	KMO	0.0023641	./../pathogenic/Benign/./././././NA/16.68	not_provided
2:37248148A>T	.	ALS_2	NDUFAF7	.	deleterious/benign/pathogenic/./0.021/0.028/0.985/2.295/T/24.5	.
2:37248187G>T	.	ALS_2	NDUFAF7	.	deleterious/probably_damaging/pathogenic/./0.991/0.924/1/3.155/D/27.7	.
2:44313844C>T	rs201143887	ALS_2; ALS_3	PREPL	8.541E-05	tolerated/benign/benign/Uncertain_significance/0.837/0.224/1/0.805/D/14.95	Cystinuria|not_provided
2:74527043G>A	rs201648655	ALS_1	HTRA2	0.0003942	tolerated/benign/benign/./0/0.001/0.946/0.55/T/17.04	.
2:96186333C>G	rs1020052	ALS_2; ALS_3	STARD7	0.0008544	./../benign/./././././NA/13.46	.
2:111166873G>A	rs1052112217	ALS_2; ALS_3	BCL2L11	6.574E-06	./../benign/./././././NA/12.58	.
2:172088063G>C	.	ALS_1	DLX1	.	tolerated/benign/pathogenic/./0.018/0.017/1/./T/22.8	.
2:197499916T>C	.	ALS_2	HSPD0	6.58E-06	./../benign/./././././NA/10.63	.
2:218665409G>T	.	ALS_2	BCS1L	.	./../benign/./././././NA/10.54	.

3:9751845G>A	rs56053615	ALS_2; ALS_3	OGG1	0.0002367	deleterious/probably_damaging/pathogenic/./1/1/1/3.145/D/31	.
3:42773260C>T	rs184880436	ALS_2	CCDC13	0.0032781	./././pathogenic/./././././NA/14.41	.
3:45394306A>C	.	ALS_2	LARS2	.	././benign/./././././NA/13.10	.
3:48593192C>T	rs147177277	ALS_1	COL7A1	0.0004275	tolerated/benign/benign/Conflicting_interpretations_of_pathogenicity/0.428/0.047/0.51/1.655/T/16.83	Epidermolysis_bullosa_dystrophica|not_provided
3:52290966G>C	rs150662353	ALS_3	GLYCTK	0.0009	deleterious/benign/pathogenic/Conflicting_interpretations_of_pathogenicity/0.11/0.011/1/1.89/T/23.2	D-Glyceric_aciduria|not_provided
3:52536876A>G	rs202198307	ALS_2	NT5DC2	0.0007232	tolerated/benign/pathogenic/./0.717/0.352/1/./T/23.1	.
3:53853161G>A	rs922324904	ALS_1	IL17RB	7.884E-05	././benign/./././././NA/10.45	.
3:58433800C>T	rs201470762	ALS_3	PDHB	9.852E-05	tolerated/benign/benign/Uncertain_significance/0.435/0.089/1/0/D/16.89	Seizures
3:121488236C>A	rs3218639	ALS_2	POLQ	0.0047364	tolerated/probably_damaging/benign/Benign/0.998/0.941/0.969/2.515/T/19.12	not_provided
3:131489865A>T	.	ALS_2	MRPL3	.	././benign/./././././NA/13.99	.
3:136328849C>T	rs142403318	ALS_1	PCCB	0.0016094	deleterious/benign/pathogenic/Benign/0.124/0.311/1/3.5/D/24.9	Propionic_acidemia|not_provided
3:158646775G>GCCCT	rs1174797887	ALS_2	GFM1	.	./././Pathogenic/./././././NA/28.8	not_provided
3:193625992C>T	rs184115665	ALS_2; ALS_3	OPA1	0.0038709	././pathogenic/./././././NA/15.02	.
4:2064573C>T	rs931445244	ALS_2	NAT8L	5.915E-05	././benign/./././././NA/10.68	.
4:39461023T>A	.	ALS_2	LIAS	.	././benign/./././././NA/17.80	.
4:48857303C>T	rs112327139	ALS_2	OCIAD1	0.003373	deleterious/benign/pathogenic/./0.162/0.045/1/0/T/23.3	.
4:108014444T>G	rs61735992	ALS_2; ALS_3	HADH	0.0051491	deleterious/probably_damaging/pathogenic/Conflicting_interpretations_of_pathogenicity/1/0.989/1/3.415/D/29.0	Hyperinsulinemic_hypoglycemia,_familial,_4|Monogenic_diabetes|Deficiency_of_3-hydroxyacyl-CoA_dehydrogenase|not_specified|not_provided
4:122929020T>A	.	ALS_2	SPATA5	.	././benign/./././././NA/13.84	.
5:195184G>A	rs190870417	ALS_2; ALS_3	CCDC127	0.0005914	tolerated/benign/benign/./0.006/0.003/1/0.705/T/12.28	.

5:110756712G>A	rs114859074	ALS_2; ALS_3	SLC25A46	0.0020151	tolerated/benign/benign/Likely_benign/0.022/0.026/0.998/1.085/T/16.02	Neuropathy,_hereditary_motor_and_sensory,_type_6B|not_specified|not_provided
5:119542077T>G	rs181310520	ALS_1	HSD17B4	0.0019912	./././benign/Likely_benign/././././NA/11.44	Perrault_syndrome_1|Bifunctional_peroxisomal_enzyme_deficiency
5:126545018T>C	rs61757684	ALS_1	ALDH7A1	0.0039483	deleterious/probably_damaging/pathogenic/Benign/Likely_benign/0.999/0.991/1/1.01/D/31	Seizures|Pyridoxine-dependent_epilepsy|not_specified|not_provided
5:131954186T>C	rs78943578	ALS_1	ACSL6	0.0044668	./pathogenic/././././NA/12.27	.
5:141929564C>A	rs201973012	ALS_1	DELE1	7.228E-05	././benign/././././NA/12.93	.
5:145760752T>G	.	ALS_2	PRELID2	.	././benign/././././NA/16.94	.
5:157734595G>A	rs146534011	ALS_2	THG1L	0.0005062	tolerated/benign/pathogenic/./0.009/0.013/0.993/1.015/T/22.3	.
6:33572563C>T	rs80065855	ALS_1	BAK1	0.0058992	././pathogenic/././././NA/15.04	.
6:87519363A>G	.	ALS_2	RARS2	.	././benign/././././NA/10.54	.
6:89637797T>A	rs151021837	ALS_2; ALS_3	LYRM2	0.0062935	tolerated/benign/pathogenic/./0.01/0.031/0.988/./T/22.2	.
6:99400159T>TA	rs879485823	ALS_3	PNISR	0.0005636	./././././././NA/12.20	.
6:106570848G>T	.	ALS_2	RTN4IP1	.	././benign/././././NA/10.07	.
6:106572113A>G	rs747032626	ALS_2	RTN4IP1	1.313E-05	././pathogenic/././././NA/22.6	.
6:159806021A>C	rs146392472	ALS_2; ALS_3	PNLDC1	0.0045154	deleterious/benign/benign/./0.328/0.085/1/2.595/T/20.6	.
8:38408237C>T	rs150451779	ALS_2	LETM2	0.0017153	deleterious/benign/benign/./0.918/0.209/1/1.1/T/18.20	.
8:80035750G>A	rs1412844732	ALS_2	TPD52	1.315E-05	././benign/././././NA/12.40	.
8:86488416A>T	.	ALS_2	RMDN1	.	././benign/././././NA/14.66	.
8:124311581CTAATA>C	rs751594860	ALS_2	TMEM65	0.0021382	./././././././NA/10.63	.
8:124313838A>G	rs189626815	ALS_1	TMEM65	0.0050806	././pathogenic/././././NA/16.79	.
8:144098948C>T	rs112552278	ALS_2	CYC1	0.0048576	deleterious/probably_damaging/pathogenic/Benign/1/0.999/1/3.055/T/26.0	not_provided
9:5361276T>C	rs1386251449	ALS_2; ALS_3	PLGRKT	3.286E-05	././benign/././././NA/11.44	.

9:32573056G>A	rs138358975	ALS_2; ALS_3	NDUFB6	1.314E-05	deleterious/probably_damaging/benign/./0.476/0.058/0.991/1.15/T/22.0	.
9:37440540T>C	rs149814405	ALS_1	GRHPR	3.941E-05	tolerated/benign/benign/./0.069/0.032/0.996/0.21/T/16.75	.
9:99915348A>G	rs748696472	ALS_2; ALS_3	STX17	3.285E-05	tolerated/benign/benign/./0.044/0.009/0.563/2.2/T/21.3	.
9:104768871G>A	.	ALS_1	NIPSNAP3B	.	deleterious/possibly_damaging/benign/./0.986/0.886/0.001/2.83/T/25.1	.
9:122265028C>T	rs190847701	ALS_3	MRRF	0.004322	./benign/./././././NA/15.38	.
9:128321713G>T	rs150599940	ALS_2; ALS_3	TRUB2	0.0005847	tolerated/benign/benign/./0.611/0.118/1/2.725/T/16.72	.
9:133364854C>T	rs781900544	ALS_1	SURF4	2.63E-05	tolerated/benign/benign/./0.003/0.003/0.815/1.355/T/19.83	.
9:136418886C>G	rs1156402838	ALS_2	PMPCA	.	deleterious/benign/pathogenic/./0.118/0.101/1/1.245/T/21.1	.
10:1048948C>A	.	ALS_2	IDI1	.	deleterious/benign/benign/./0.061/0.026/1/./T/12.76	.
10:13278305A>AGAT	rs566116760	ALS_1	PHYH	0.0015965	./././Conflicting_interpretations_of_pathogenicity/./././././NA/15.36	Nonsyndromic_cleft_lip_palate|not_provided
10:49739779A>G	rs143105288	ALS_2	OGDHL	0.0062629	deleterious/probably_damaging/pathogenic/Likely_benign/0.996/0.954/1/4.385/D/31	Inborn_genetic_diseases
10:73136897T>G	rs147899211	ALS_3	ECD	0.0005066	deleterious/probably_damaging/benign/./1/1/1/2.69/T/25.9	.
10:100978532G>A	rs769114862	ALS_3	MRPL43	9.202E-05	tolerated/benign/pathogenic/./0.503/0.077/1/0.715/T/21.4	.
10:102727138T>TA	rs1212464229	ALS_2; ALS_3	SFXN2	1.315E-05	./././././././././NA/32	.
10:102727140C>G	rs1017663995	ALS_2; ALS_3	SFXN2	1.315E-05	tolerated/benign/pathogenic/./0.001/0.004/1/0.19/T/18.84	.
10:133381501C>T	rs150446594	ALS_2	PAOX	0.0005189	deleterious/probably_damaging/pathogenic/./1/0.994/1/3.295/D/23.8	.
11:230474G>T	rs61748606	ALS_2; ALS_3	SIRT3	0.0098196	deleterious/possibly_damaging/benign/Benign/0.971/0.838/1/1.12/T/22.7	not_provided
11:34966542T>G	rs147215008	ALS_2	PDHX	0.005184	./benign/./././././NA/10.57	.
11:47617440T>C	.	ALS_2	MTCH2	.	./benign/./././././NA/16.18	.
11:66852917G>A	rs780941915	ALS_2	PC	0.0006375	./benign/./././././NA/11.12	.

11:66871069C>A	rs147945506	ALS_2	PC	0.0016429	deleterious/benign/pathogenic/Conflicting_interpretations_of_pathogenicity/0.603/0.389/1/1.48/D/23.3	Pyruvate_carboxylase_deficiency|not_provided
11:67606954CCTCAGTGCT>C	.	ALS_2	NDUFV1	.	./././././././././10.18	.
11:67606965TGAAGGTGACAG>T	.	ALS_2	NDUFV1	.	//././/////20.9	.
11:72356486G>T	.	ALS_2	CLPB	.	././benign/./././././NA/19.88	.
12:25204247T>TA	rs1323271219	ALS_1	ETFRF1	6.569E-06	././././././././NA/34	.
12:32730865A>T	.	ALS_2	DNM1L	.	././benign/./././././NA/10.09	.
12:50106297G>A	rs34783513	ALS_2; ALS_3	GPD1	0.0013671	tolerated/benign/pathogenic/Benign/Likely_benign/0.005/0.004/1/1.975/T/19.70	not_provided
12:51052368T>A	.	ALS_2	LETMD1	.	././benign/./././././NA/11.94	.
12:55992611GAGC>G	rs772313510	ALS_2; ALS_3	SUOX	0.0002433	././././././././NA/11.78	.
12:56268861T>G	.	ALS_2	COQ10A	.	././benign/./././././NA/17.49	.
12:57765659C>T	rs35569378	ALS_3	CYP27B1	0.0059116	././benign/Likely_benign/././././NA/10.14	not_provided
12:57772807C>T	rs34913183	ALS_2	CYP27B1	0.0086529	tolerated/benign/benign/./0.196/0.016/1/0.895/T/22.5	.
12:57780492T>C	rs111299874	ALS_2	TSFM	0.0060924	deleterious/possibly_damaging/benign/./0.818/0.559/0.973/2.395/T/24.8	.
12:81143432T>C	.	ALS_2	ACSS3	.	././benign/./././././NA/11.85	.
12:98593876G>C	rs976686538	ALS_2; ALS_3	SLC25A3	6.571E-05	././benign/./././././NA/17.49	.
12:98595256C>A	.	ALS_2	SLC25A3	.	././benign/./././././NA/10.93	.
12:105052736A>T	.	ALS_2	ALDH1L2	.	././benign/./././././NA/12.85	.
12:111748449C>T	rs150643910	ALS_2	ACAD10	6.571E-05	tolerated/benign/benign/./0.904/0.328/0.988/2.16/D/13.91	.
12:113291233C>A	rs1956256422	ALS_2	TPCN1	6.571E-06	././benign/./././././NA/11.56	.
12:120312595C>T	rs138164230	ALS_1	SIRT4	0.0035229	deleterious/possibly_damaging/benign/./0.922/0.67/0.753/2.36/T/23.3	.
13:41886121T>A	.	ALS_2	VWA8	.	././benign/./././././NA/12.35	.
14:24306420G>A	rs148872765	ALS_1	NOP9	0.000611	deleterious/probably_damaging/pathogenic/./1/0.982/1/3.435/T/27.2	.

14:36729308T>A	.	ALS_2	SLC25A21	.	./.benign/./././././NA/16.07	.
14:36729648A>G	rs1884743435	ALS_2	SLC25A21	.	./.benign/./././././NA/18.05	.
14:36874765G>A	rs775098279	ALS_1	SLC25A21	0.0002563	./.pathogenic/./././././NA/14.84	.
14:74500948C>T	rs143010135	ALS_2	ISCA2	0.0003417	tolerated/benign/benign/Uncertain_significance/0.309/0.053/0.955/1.35/T/23.1	Glaucoma_3,_primary_congenital,_d|Weill-Marchesani_syndrome|not_provided
14:74881864G>A	rs991279742	ALS_2	DLST	0.0001183	./.benign/./././././NA/11.32	.
15:65155732T>C	rs147893822	ALS_2	CLPX	0.0001051	tolerated/benign/benign/./0.623/0.401/1/1.325/T/21.4	.
15:76274185T>C	rs139097487	ALS_3	ETFA	0.0069497	./.benign/Likely_benign/./././././NA/11.72	not_provided
16:683424C>G	rs72773413	ALS_1	JMJD8	0.0040884	deleterious/probably_damaging/pathogenic/./1/1/1/3.07/T/28.9	.
16:1414080C>G	rs201417307	ALS_3	UNKL	0.0027859	./.benign/./././././NA/10.29	.
16:1984584C>A	rs550296574	ALS_2; ALS_3	GFER	0.0021418	./.benign/./././././NA/10.08	.
16:1993259C>T	rs958228085	ALS_2	SYNGR3	3.284E-05	./.benign/./././././NA/14.19	.
16:20418224C>T	rs144548629	ALS_2	ACSM5	0.0080265	deleterious/probably_damaging/pathogenic/./1/0.999/1/4.305/T/21.6	.
16:20777354T>A	.	ALS_2	ACSM3	.	./.pathogenic/./././././NA/15.15	.
16:20790494T>C	rs1208479326	ALS_2	ACSM3	6.57E-06	./.benign/./././././NA/12.96	.
16:28846253G>A	rs1389713655	ALS_2	TUFM	1.315E-05	tolerated/benign/benign/./0/0.001/0.985/0/T/14.37	.
16:68078931A>G	rs140584572	ALS_2	DUS2	3.941E-05	deleterious/benign/benign/./0.024/0.012/0.547/1.04/T/23.3	.
17:43175696C>T	rs117439494	ALS_2	NBR1	0.0046485	./.benign/./././././NA/11.57	.
17:50112976C>T	rs201142482	ALS_1	PDK2	0.0002168	deleterious/possibly_damaging/pathogenic/./0.94/0.197/0.999/2.05/T/25.8	.
17:64506909G>A	rs149693682	ALS_3	CEP95	0.0037114	./.pathogenic/./././././NA/16.62	.
17:75003577G>T	rs187215004	ALS_3	CDR2L	0.0005715	tolerated/possibly_damaging/benign/./0.995/0.83/1/1.845/T/22.9	.
17:75289560T>A	rs182532666	ALS_2	SLC25A19	0.0020367	./.benign/./././././NA/16.07	.
17:81941891GCTGT>G	rs200797941	ALS_2; ALS_3	PYCR1	0.0073977	./././././././././NA/13.94	.
18:46098232C>T	rs546539852	ALS_1	ATP5F1A	0.0002234	./.benign/./././././NA/22.0	.
19:1105691G>A	rs76201145	ALS_1	GPX4	0.0006963	tolerated/benign/pathogenic/./0.063/0.069/1/1.635/T/23.2	.

19:12904533G>A	rs189296436	ALS_2; ALS_3	GCDH	0.0026557	tolerated/probably_damaging/pathogenic/./0.996/0.937/0.904/1.87/D/22.8	.
19:12948812C>T	rs4987202	ALS_2; ALS_3	GADD45GIP1	0.0072724	tolerated/benign/benign/./0.007/0.009/0.992/1.1/T/22.5	.
19:19535632C>G	rs146733860	ALS_3	YJEFN3	0.0052825	tolerated/probably_damaging/benign/./1/0.992/1/1.83/T/23.6	.
19:45768141G>A	rs1014350413	ALS_2; ALS_3	DMPK	6.568E-05	deleterious/possibly_damaging/pathogenic/./0.99/0.862/1/3.295/D/29.1	.
19:48613266T>G	rs1205554355	ALS_2	FAM83E	.	deleterious/probably_damaging/pathogenic/./1/0.999/1/3.32/T/25.0	.
19:48636843G>T	rs150228428	ALS_1	CA11	0.0086136	./benign/./././././NA/16.35	.
20:6041600A>T	rs145844426	ALS_1	CRLS1	0.0038832	deleterious/probably_damaging/pathogenic/./0.995/0.922/1/2.9/D/26.9	.
20:17970372T>A	.	ALS_2	MGME1	.	././pathogenic/./././1/./NA/34	.
22:17554179G>T	.	ALS_2	CECR2	.	././benign/./././././NA/14.62	.
22:19435831C>T	rs138093372	ALS_3	MRPL40	0.000184	tolerated/possibly_damaging/pathogenic/./0.999/0.867/1/2.89/T/22.6	.
22:29767548G>GAA	rs749795682	ALS_2	UQCR10	2.634E-05	././././././././NA/33	.
22:29767549G>GCTGTGGAAACACATCAAGCACAAGTA	rs760862598	ALS_2	UQCR10	2.17E-05	././././././././NA/25.8	.
22:30416398G>A	rs191341134	ALS_3	SEC14L2	3.942E-05	deleterious/probably_damaging/pathogenic/./1/1/1/3.795/D/28.8	.
22:39513721G>A	rs2232091	ALS_2	MIEF1	0.0061948	tolerated/benign/pathogenic/./0.077/0.058/1/-0.11/T/23.5	.
22:50217114G>A	rs34163881	ALS_2; ALS_3	SELENOO	0.0074234	deleterious/probably_damaging/pathogenic/./0.998/0.931/1/3.365/T/28.1	.

Table S3. nDNA genes containing variants in the WES/WGS datasets associated with Amyotrophic Lateral Sclerosis as identified by DisGeNet

	Gene	Gene Name	* gda Score
WES dataset	*DNM1L	dynamin 1 like	0.05
	SIRT3	sirtuin 3	0.02
	*ATP5F1A	ATP synthase F1 subunit alpha	0.01
	*OPA1	OPA1 mitochondrial dynamin like GTPase	0.01
	*PARK7	Parkinsonism associated deglycase	0.01
	HTRA2	HtrA serine peptidase 2	0.01
	IDI1	isopentenyl-diphosphate delta isomerase 1	0.01
WGS dataset	GSR	glutathione-disulfide reductase	0.32
	CASP3	caspase 3	0.29
	SLC25A12	solute carrier family 25 member 12	0.1
	PINK1	PTEN induced kinase 1	0.06
	FGF2	fibroblast growth factor 2	0.05
	OXR1	oxidation resistance 1	0.04
	VDAC1	voltage dependent anion channel 1	0.04
	CYP27A1	cytochrome P450 family 27 subfamily A member 1	0.03
	FXN	frataxin	0.03
	TFAM	transcription factor A, mitochondrial	0.03
	GLS	glutaminase	0.02
	PRKN	parkin RBR E3 ubiquitin protein ligase	0.02
	SLC25A37	solute carrier family 25 member 37	0.02
	ATAT1	alpha tubulin acetyltransferase 1	0.01
	*ATP5F1A	ATP synthase F1 subunit alpha	0.01
	BNIP3L	BCL2 interacting protein 3 like	0.01
	CASP8	caspase 8	0.01
	DLD	dihydrolipoamide dehydrogenase	0.01
	*DNM1L	dynamin 1 like	0.01
	HADHA	hydroxyacyl-CoA dehydrogenase trifunctional multienzyme complex subunit alpha	0.01
	ITPR3	inositol 1,4,5-trisphosphate receptor type 3	0.01
	MAP1B	microtubule associated protein 1B	0.01
	MCU	mitochondrial calcium uniporter	0.01
	MRPS30	mitochondrial ribosomal protein S30	0.01
	NIF3L1	NGG1 interacting factor 3 like 1	0.01
	NLN	neurolysin	0.01
	*OPA1	OPA1 mitochondrial dynamin like GTPase	0.01
	*PARK7	Parkinsonism associated deglycase	0.01
	PDAP1	PDGFA associated protein 1	0.01
	PDSS1	decaprenyl diphosphate synthase subunit 1	0.01
	SIRT5	sirtuin 5	0.01

gda Score: DisGeneNet database gives a score of this association from 0 to 1 based on type of sources (level of curation, model organisms) and the number of publications supporting the association. * Genes containing variants that overlapped in the two datasets.

Table S4. Genes alphabetically ordered containing at least three variants that have passed variant prioritization filters in Amyotrophic Lateral Sclerosis (ALS) patients in the Whole Exome Sequencing (WES) and Whole Genome Sequencing (WGS) datasets.

OFFICIAL GENE SYMBOL	Gene Name	N° Variants WGS (WES)	WGS	WES	DisGeNET	ALSoD
ACACA	acetyl-CoA carboxylase alpha	4(0)	X			
ACSM3	acyl-CoA synthetase medium chain family member 3	2(2)	X	X		
AFG1L	AFG1 like ATPase	6(0)	X			
ALDH1L1	aldehyde dehydrogenase 1 family member L1	3(0)	X			
ARG2	arginase 2	3(0)	X			
ATP5F1D	ATP synthase F1 subunit delta	3(0)	X			
BCKDHB	branched chain keto acid dehydrogenase E1 subunit beta	6(0)	X			
BCL2	BCL2 apoptosis regulator	4(0)	X			
BCL2L1	BCL2 like 1	3(0)	X			
CCDC51	coiled-coil domain containing 51	3(0)	X			
CHCHD3	coiled-coil-helix-coiled-coil-helix domain containing 3	10(0)	X			
CHCHD6	coiled-coil-helix-coiled-coil-helix domain containing 6	7(0)	X			
CLPB	ClpB family mitochondrial disaggregase	1(2)	X	X		
CLYBL	citramalyl-CoA lyase	11(0)	X			
COMT	catechol-O-methyltransferase	3(0)	X			
CRLS1	cardiolipin synthase 1	2(1)	X	X		
CYB5R3	cytochrome b5 reductase 3	3(0)	X			
DBT	dihydrolipoamide branched chain transacylase E2	3(0)	X			
DELE1	DAP3 binding cell death enhancer 1	2(1)	X	X		
DMGDH	dimethylglycine dehydrogenase	3(0)	X			
DMPK	DM1 protein kinase	1(2)	X	X		
DNAJC11	DnaJ heat shock protein family	4(2)	X	X		
ETFA	electron transfer flavoprotein subunit alpha	5(1)	X	X		
FHIT	fragile histidine triad diadenosine triphosphatase	26(0)	X			
GADD45GIP1	GADD45G interacting protein 1	1(2)	X	X		
GATM	glycine amidinotransferase	3(0)	X			
GFER	growth factor, augmenter of liver regeneration	2(2)	X	X		
GLDC	glycine decarboxylase	3(0)	X			
GLS	glutaminase	3(0)	X		X	
GPD2	glycerol-3-phosphate dehydrogenase 2	7(0)	X			
HIBADH	3-hydroxyisobutyrate dehydrogenase	4(0)	X			
IDE	insulin degrading enzyme	5(0)	X			

IMMP2L	inner mitochondrial membrane peptidase subunit 2	11(0)	X			
LARS2	leucyl-tRNA synthetase 2, mitochondrial	2(1)	X	X		
LYRM2	LYR motif containing 2	1(2)	X	X		
MCU	mitochondrial calcium uniporter	3(0)	X		X	
METAP1D	methionyl aminopeptidase type 1D, mitochondrial	5(0)	X			
METTL8	methyltransferase 8, tRNA N3-cytidine	4(0)	X			
MICU2	mitochondrial calcium uptake 2	3(0)	X			
MRPL1	mitochondrial ribosomal protein L1	3(0)	X			
MRPS27	mitochondrial ribosomal protein S27	3(0)	X			
MRPS6	mitochondrial ribosomal protein S6	5(0)	X			
MRRF	mitochondrial ribosome recycling factor	5(1)	X	X		
MSRA	methionine sulfoxide reductase A	20(0)	X			
MTHFD1L	methylenetetrahydrofolate dehydrogenase	3(0)	X			
MTHFD2L	methylenetetrahydrofolate dehydrogenase	5(0)	X			X
MTX2	metaxin 2	5(0)	X			
NARS2	asparaginyl-tRNA synthetase 2, mitochondrial	4(0)	X			
NBR1	NBR1 autophagy cargo receptor	2(1)	X	X		
NDUFS2	NADH:ubiquinone oxidoreductase core subunit S2	3(0)	X			
NDUFV2	NADH:ubiquinone oxidoreductase core subunit V2	4(0)	X			
NRDC	nardilysin convertase	2(1)	X	X		
NUBPL	NUBP iron-sulfur cluster assembly factor, mitochondrial	4(0)	X			
OCIAD1	OCIA domain containing 1	2(1)	X	X		
OGG1	8-oxoguanine DNA glycosylase	2(2)	X	X		X
OPA1	OPA1 mitochondrial dynamin like GTPase	2(2)	X	X	X	
OSBPL1A	oxysterol binding protein like 1A	3(0)	X			
OXCT1	3-oxoacid CoA-transferase 1	3(0)	X			
OXR1	oxidation resistance 1	7(0)	X		X	
PARK7	Parkinsonism associated deglycase	1(2)	X	X	X	X
PC	pyruvate carboxylase	2(3)	X	X		
PCCA	propionyl-CoA carboxylase subunit alpha	6(0)	X			
PDE2A	phosphodiesterase 2A	4(0)	X			
PDHX	pyruvate dehydrogenase complex component X	2(1)	X	X		
PDSS1	decaprenyl diphosphate synthase subunit 1	3(0)	X		X	
PDSS2	decaprenyl diphosphate synthase subunit 2	6(0)	X			
PNKD	PNKD metallo-beta-lactamase domain containing	3(0)	X			
PNPLA8	patatin like phospholipase domain containing 8	3(0)	X			
POLQ	DNA polymerase theta	2(1)	X	X		
PRELID2	PRELI domain containing 2	1(4)	X	X		
PREPL	prolyl endopeptidase like	1(2)	X	X		
RARS2	arginyl-tRNA synthetase 2, mitochondrial	2(1)	X	X		
RTN4IP1	reticulon 4 interacting protein 1	1(2)	X	X		

SFXN2	sideroflexin 2	0(4)		X
SFXN5	sideroflexin 5	5(0)	X	
SHMT2	serine hydroxymethyltransferase 2	4(0)	X	
SLC25A21	solute carrier family 25 member 21	15(3)	X	X
SLC25A26	solute carrier family 25 member 26	5(0)	X	
SLC25A3	solute carrier family 25 member 3	0(3)		X
SLC25A35	solute carrier family 25 member 35	4(0)	X	
SND1	staphylococcal nuclease and tudor domain containing 1	11(0)	X	
SPHKAP	SPHK1 interactor, AKAP domain containing	4(0)	X	
SPIRE1	spire type actin nucleation factor 1	3(0)	X	
STX17	syntaxin 17	3(2)	X	X
SUGCT	succinyl-CoA:glutarate-CoA transferase	7(0)	X	
SUOX	sulfite oxidase	1(2)	X	X
TMEM65	transmembrane protein 65	2(1)	X	X
TSFM	Ts translation elongation factor, mitochondrial	2(1)	X	X
VPS13D	vacuolar protein sorting 13 homolog D	1(5)	X	X
VWA8	von Willebrand factor A domain containing 8	4(1)	X	X

Table S5. List of 12 prioritized mtDNA variant sites in the WES cohort

Locus	Variant Position	Variant ID	Variant nature	N° Cases	AF Het	AF Hom	Pathogenicity (Hmtdb)	ClinVarSignificance/MutPred_Prediction/Panther_Prediction/PhDSNP_Prediction/SNPsGO_Prediction/Polyphen2HumDiv_Prediction/Polyphen2HumVar_Prediction	pon_tRNA /MitoTIP_ prediction	Mitomap Disease	ClinVar's disease name
CR - HV2	152T>C	rs117135796	Het	ALS_2	0.00342		pending classification	-/././././././			
CR - HV2	195T>C	.	Het	ALS_1	0.00240753			-/././././././			
CR - HV2	198C>T	rs879040416	Het	ALS_1	0.000461525			-/././././././		BD-associated/ melanoma pts	
CR - HV3	456C>T	rs41356551	Het	ALS_2	0.000124		pending classification,	-/././././././			
12 S	721T>C	rs1556422479	Het	ALS_2	0.0000354			Likely benign/././././././		Possibly LVNC-associated	not provided
12 S	1007G>A	rs111033213	Het/ Hom	ALS_2 ALS_3	0.000124	0.00108	pending classification	Benign/././././././			not specified
MT-TQ	4336T>C	rs41456348	Het	ALS_2	0.0000177		likely pathogenic, tRNA	Conflicting interpretations of pathogenicity/././././././	Neutral/ possibly benign	ADPD / Hearing Loss & Migraine / autism spectrum / intellectual disability	Juvenile myopathy, encephalopathy, lactic acidosis AND stroke Sensori-neural deafness and migraine
MT-CO1	7410C>T	.	Het	ALS_2	/		pathogenic	-/low_pathogenicity/neutral/disease/neutral/probably_damaging/possibly_damaging/			
MT-CO2	8027G>A	rs1116904	Het/Hom	ALS_2 ALS_3	0.000124	0.074	likely pathogenic	Benign/low_pathogenicity/neutral/neutral/neutral/probably_damaging/probably_damaging/			Leigh syndrome
MT-TS2	12235T>C	rs1556424083	Het/ Hom	ALS_2 ALS_3	0.0000532	0.000514	polymorphic, tRNA	Benign/././././././	Neutral/ possibly benign	Hearing loss	Juvenile myopathy, encephalopathy, lactic acidosis AND stroke
CR - HV1	16240A>G	rs386829288	Het/ Hom	ALS_2 ALS_3	0.000177	0.00239	pending classification,	-/././././././			
CR - HV1	16304T>C	rs386829305	Het	ALS_2	0.000142		pending classification,	-/././././././			

Table S6. Statistics related to the Whole Genome Sequencing Dataset

STATUS	ALS1_497	ALS2_503	ALS3_536	ALS4_539	ALS5_5608	ALS6_5609	ALS7_5610	ALS8_5611	CNTR1_596	CNTR2_597	CNTR3_598	CNTR4_599	Mean
FASTQ FILES													
read count r1	460675189	451060045	502247526	449148192	426007105	487763586	421977005	391392148	532843239	500413481	550409446	551923000	477154997
read count r2	460675189	451060045	502247526	449148192	426007105	487763586	421977005	391392148	532843239	500413481	550409446	551923000	477154997
total read count	921350378	902120090	1004495052	898296384	852014210	975527172	843954010	782784296	1065686478	1000826962	1100818892	1103846000	954309994
nDNA Variant in Mitocarta genes													
n.extended Mitocarta	109570	106053	107555	106698	97874	98890	97096	98893	126826	119187	114456	107024	107510
median depth	31	30	31	29	26	29	26	24	36	35	34	35	30
MAPPING DATA to mtDNA													
n°Reads	2470063	2362993	3050243	3077180	3229335	3825246	3428085	3631458	5070990	6179947	8853880	6572442	4312655
% mapped reads to chrM	100	100	100	100	100	100	100	100	100	100	100	100	100
mean coverage target region	17872	16874	21638	21368	19637	22382	20685	21927	31061	36337	43373	35549	25725
mean mapping quality	40	40	40	40	37	37	37	37	40	40	40	40	39
duplicate reads	486135	489778	647242	705166	1036029	1323948	1117521	1182530	1620900	2145069	4040421	2628729	1451956
% duplicate reads	20%	21%	21%	23%	32%	35%	33%	33%	32%	35%	46%	40%	31%
GC Percentage	45%	45%	45%	45%	44%	44%	44%	44%	45%	45%	44%	44%	44%
mtDNA Variant Calling													
n.variants	69	95	35	16	15	36	41	14	180	73	55	16	54
homoplasmic	14	27	29	13	13	33	27	11	66	32	35	12	26
heteroplasmic	55	68	6	4	2	3	14	3	114	41	21	4	28
median depth	2130	3235	9640	11990	10538	10242	6232	12777	1991	10428	16278	26761	10187

Table S7. List of 842 nDNA variant sites ranked by priority in the WGS dataset.

Variant Position	ID	N° Cases	GeneName	AF	SIFT/PolyPhen/FATHMM_score/CLNSIG/Polyphen2 HDIV/Polyphen2 HVAR/MutationTaster/Mutatio.ssessor/MetaSVM Score/CADD_PHRED Score	CLNDN
1:1303203G>A	rs145087137	ALS2_503	ACAP3	0.007621	deleterious/possibly_damaging/pathogenic/./0.763/0.245/0.996/1.905/T/28.5	.
1:1304812G>A	rs138176063	ALS2_503	PUSL1	0.00934	./../benign/./././././../12.85	.
1:1305568G>A	rs746318266	ALS2_503	PUSL1	0.000342	./../benign/./././././../10.09	.
1:1462652C>G	rs181068440	ALS2_503; ALS7_5610	ATAD3C	0.003873	tolerated/benign/benign/./0.011/0.007/0.959/2.075/T/12.80	.
1:6289723G>T	rs187044080	ALS8_5611	ACOT7	0.001637	./../benign/./././././../10.34	.
1:6652197G>C	rs560875462	ALS3_536	DNAJC11	0.003279	./../benign/./././././../10.68	.
1:6665523T>C	rs755432636	ALS4_539	DNAJC11	0.000223	./../pathogenic/./././././../11.08	.
1:6681067A>C	rs41310361	ALS5_5608	DNAJC11	0.003548	./../benign/./././././../12.95	.
1:6701331G>A	rs141622479	ALS7_5610	DNAJC11	0.003896	./../benign/./././././../12.11	.
1:7962280G>A	rs1449679359	ALS8_5611	PARK7	6.58E-06	./../benign/./././././../12.62	.
1:9539454C>G	rs1316540335	ALS1_497	SLC25A33	1.98E-05	./../benign/./././././../18.16	.
1:9588674C>T	rs567615396	ALS5_5608	SLC25A33	0.002117	./../benign/./././././../10.08	.
1:12266959G>A	rs375749880	ALS8_5611	VPS13D	0	tolerated/benign/benign/./0.006/0.01/0.739/1.245/T/22.7	.
1:12332992T>C	.	ALS3_536	VPS13D	0	./../benign/./././././../10.86	.
1:12406861G>A	rs147534406	ALS3_536	VPS13D	0.009668	./../benign/./././././../15.17	.
1:12467619C>G	rs550692826	ALS4_539	VPS13D	0.001636	./../pathogenic/./././././../16.72	.
1:12481581G>A	rs149525503	ALS2_503	VPS13D	0.002668	./../benign/./././././../15.26	.
1:20654663G>A	rs142593688	ALS7_5610	PINK1	0.000493	./../pathogenic/Uncertain_significance/0.991/0.87/1/2.4/D/25.1	Congenital_disorder_of_glycosylation_type_Ir
1:33036780G>C	rs138577419	ALS1_497	AK2	0.001295	deleterious/benign/pathogenic/Conflicting_interpretations_of_pathogenicity/0.877/0.54/1/2.45/T/25.4	Reticular_dysgenesis|not_provided
1:36471505C>T	rs3918019	ALS5_5608	CSF3R	0.004671	tolerated/benign/benign/Benign/0.915/0.217/0.989/1.84/T/17.76	Neutropenia,_severe_congenital,_7,_autosomal_recessive|not_specified
1:37808187A>G	rs560598698	ALS1_497	YRDC	0.001296	./../benign/./././././../11.16	.
1:37809850A>G	.	ALS4_539	YRDC	6.57E-06	./../benign/./././././../14.59	.
1:39021936T>G	rs184314670	ALS7_5610	NDUFS5	0.003429	./../benign/./././././../13.31	.
1:39857977GATCT>G	rs993821921	ALS7_5610	TRIT1	3.94E-05	./././././././././../17.13	.
1:45326675C>T	rs571489423	ALS4_539	HPDL	0.002551	./../benign/./././././../11.83	.
1:45331467G>A	rs150792276	ALS4_539	MUTYH	0.000802	deleterious/benign/benign/Conflicting_interpretations_of_pathogenicity/0.995/0.827/0.973/1.5/T/16.64	Carcinoma_of_colon|MYH-associated_polyposis|Hereditary_

1:46298275G>C	rs148912617	ALS8_5611	LRRC41	0.002517	./ ./pathogenic/Benign/./././././10.19	cancer-predisposing_syndrome|not_specified|not_provided|not_provided
1:46672839C>T	rs932893232	ALS2_503	ATPAF1	0	././benign/./././././././13.86	.
1:51855542A>C	rs758534494	ALS2_503	NRDC	0.000302	././benign/./././././././14.82	.
1:51856928TACAAGCC>T	rs568627193	ALS7_5610	NRDC	0.001209	./././././././././././12.13	.
1:52978368G>T	rs144132787	ALS6_5609	SCP2	7.88E-05	././pathogenic/Likely_pathogenic/././1/././34	Leukoencephalopathy_with_dystonia_and_motor_neuropathy
1:53216053C>T	rs113278028	ALS3_536	C1orf123	0.004605	deleterious/possibly_damaging/pathogenic/./0.894/0.499/1/2.225/T/26.3	.
1:54619975C>T	rs17399297	ALS6_5609	ACOT11	0.005731	././pathogenic/Likely_benign/0.238/0.041/1/1.625/T/23.0	not_provided
1:77821390A>AT	rs199521038	ALS3_536	MIGA1	0.011389	./././././././././././13.39	.
1:77844376A>G	rs72685319	ALS7_5610	MIGA1	0.004505	././benign/./././././././13.32	.
1:88980424A>AT	rs879210454	ALS1_497	KYAT3	0.003464	./././././././././././11.73	.
1:88983729C>T	rs139527615	ALS4_539	KYAT3	0.006161	././benign/./0.81/0.195/1/0.49/T/18.95	.
1:94418961G>C	rs768374353	ALS2_503	ABCD3	1.31E-05	././benign/./././././././10.24	.
1:100203419A>G	rs912744456	ALS1_497	DBT	1.97E-05	././benign/./././././././12.03	.
1:100239137G>A	rs748935878	ALS7_5610	DBT	2.63E-05	././benign/./././././././12.61	.
1:100243809C>T	rs193012505	ALS7_5610	DBT	0.002687	././benign/./././././././10.61	.
1:108154533C>T	rs181542559	ALS4_539	SLC25A24	0.00021	././benign/./././././././14.84	.
1:110006905T>A	rs149653198	ALS5_5608	AHCYL1	0.004153	././benign/./././././././13.07	.
1:110023261G>A	rs140578184	ALS8_5611	AHCYL1	0.003996	././pathogenic/./././././././16.30	.
1:110028748C>CT	rs949300125	ALS1_497	STRIP1	0.000758	./././././././././././13.55	.
1:119140608A>C	rs139548132	ALS1_497	WARS2	0.003152	tolerated/benign/benign/Conflicting_interpretations_of_pathogenicity/0.001/0.002/0.963/2.14/T/17.14	Neurodevelopmental_disorder,_mitochondrial,_with_abnormal_movements_and_lactic_acidosis,_with_or_without_seizures|not_provided
1:119393538A>C	rs587702397	ALS7_5610	HAO2	0.000164	././benign/./././././././10.13	.
1:147639575T>A	.	ALS1_497	ACP6	0	././benign/./././././././17.39	.
1:147639576C>A	.	ALS1_497	ACP6	0	././benign/./././././././16.96	.
1:150302541C>T	rs188389030	ALS1_497	MRPS21	0.005442	././benign/./././././././12.33	.
1:150572917TAA>T	rs3839013	ALS1_497; ALS3_536	MCL1	0.007153	./././././././././././12.24	.
1:151770979T>C	rs192372329	ALS7_5610	TDRKH	0.001524	././benign/./././././././12.24	.
1:154979084C>T	rs545522828	ALS1_497	FLAD1	0.001039	././benign/./././././././11.31	.
1:154999693C>T	rs546726851	ALS6_5609	ZBTB7B	0.002248	././benign/./././././././10.43	.
1:155218096T>C	.	ALS5_5608	MTX1	0	././pathogenic/./././././././18.74	.
1:155218462C>T	rs191564448	ALS3_536	MTX1	0.001847	././benign/./././././././11.79	.

1:156193343CAGG>C	rs529915033	ALS4_539	SLC25A44	0.002892	./././././././././13.61	.
1:156747328C>A	rs746709931	ALS2_503	HDGF	0.000263	././benign/./././././././16.76	.
1:161038950C>T	rs114452711	ALS3_536	TSTD1	0.001788	././benign/./././././././11.65	.
1:161120749G>A	rs376379316	ALS7_5610	NIT1	6.57E-05	tolerated/benign/pathogenic/./0.089/0.057/0.957/-0.55/T/17.98	.
1:161131872T>G	rs189421017	ALS7_5610	DEDD	0.000414	././benign/./././././././10.71	.
1:161198357T>C	rs61747495	ALS1_497; ALS2_503	NDUFS2	0.004025	././benign/./0.101/0.061/0.999/1.355/T/17.09	.
1:161219108G>A	rs927999045	ALS5_5608	NDUFS2	1.97E-05	././benign/./././././././10.64	.
1:161330269A>G	rs571270748	ALS7_5610	SDHC	1.97E-05	././benign/./././././././10.02	.
1:165675863T>C	rs552342371	ALS7_5610	ALDH9A1	0.000217	././benign/./././././././11.53	.
1:165680614C>G	rs1065756	ALS3_536	ALDH9A1	0.017599	tolerated/benign/benign/./0/0.001/1/-0.395/T/11.50	.
1:167880620G>A	rs146573590	ALS8_5611	ADCY10	0.007983	././benign/./././././././16.21	.
1:167924321A>G	rs143704144	ALS3_536	MPC2	9.2E-05	././benign/./././././././14.82	.
1:167938112A>G	rs556318456	ALS6_5609	MPC2	0.000158	././benign/./././././././10.78	.
1:167944301A>G	rs1277515226	ALS7_5610	DCAF6	3.28E-05	././benign/./././././././15.51	.
1:173835452T>C	rs1036874881	ALS7_5610	DARS2	0.000749	././benign/./././././././10.70	.
1:220137982T>C	rs1488422152	ALS8_5611	IARS2	2.63E-05	tolerated/benign/pathogenic/./0.515/0.356/0.995/1.615/T/22.0	.
1:226990226CT>C	.	ALS8_5611	COQ8A	0	./././././././././11.24	.
1:228157738A>G	rs1571907281	ALS6_5609	GJC2	3.39E-05	././pathogenic/./././././././31	.
1:234373657C>G	rs117011051	ALS8_5611	COA6	0.00433	tolerated/benign/benign/Benign/././1/./T/12.28	not_specified
2:26223716C>T	rs572122315	ALS3_536	HADHA	0.00248	././benign/./././././././12.68	.
2:43967464G>A	rs1346113357	ALS2_503	LRPPRC	2.63E-05	././benign/./././././././11.84	.
2:44312539A>G	.	ALS7_5610	PREPL	0	././benign/./././././././14.51	.
2:55669490T>C	rs112939231	ALS4_539	PNPT1	0.005544	././benign/./././././././13.17	.
2:69389670A>G	rs144607557	ALS7_5610	GFPT1	0.00452	././benign/./././././././12.72	.
2:72893070A>C	.	ALS8_5611	SPR	0.000349	././benign/./././././././10.42	.
2:72934354G>C	rs569604109	ALS8_5611	EMX1	0.001281	././benign/./././././././12.26	.
2:72940760G>T	.	ALS4_539	SFXN5	0	././benign/./././././././10.33	.
2:72948099G>A	rs183314214	ALS6_5609	SFXN5	0.000118	././benign/./././././././16.77	.
2:72972747C>A	rs72918389	ALS6_5609	SFXN5	0.002826	././benign/./././././././16.80	.
2:72972962T>C	rs750429330	ALS1_497	SFXN5	0.000178	././benign/./././././././18.44	.
2:73020681T>C	rs927706146	ALS6_5609	SFXN5	3.28E-05	././benign/./././././././10.93	.
2:74206108T>C	rs762916557	ALS4_539	MTHFD2	0.000223	././benign/./././././././12.61	.
2:74538538G>A	rs556244881	ALS5_5608	LOXL3	0.000171	././benign/./././././././14.52	.
2:74539441A>C	rs538765615	ALS4_539	LOXL3	0.000211	././benign/./././././././11.22	.
2:86121020T>C	rs146520394	ALS2_503	PTCD3	0.006201	././benign/./././././././10.04	.
2:86183689A>G	rs188548908	ALS7_5610	IMMT	0.003864	././benign/./././././././10.30	.
2:86207193G>A	rs1132002	ALS1_497	MRPL35	0.00545	deleterious/benign/benign/./0.585/0.022/0.985/0/T/17.27	.

2:88692002G>A	rs114563957	ALS8_5611	RPIA	0.004541	./benign/./././././13.22	.
2:88719378C>T	rs146913994	ALS3_536	RPIA	0.000776	./benign/./././././10.17	.
2:99170686GTTA>G	rs770728381	ALS5_5608	MITD1	0.001113	./././././././10.12	.
2:99171684A>T	rs188688938	ALS4_539	MITD1	0.005245	./benign/./././././16.94	.
2:105044506A>G	rs181081347	ALS6_5609	MRPS9	0.005906	./benign/./././././13.77	.
2:156435440GT>G	rs1175590351	ALS2_503	GPD2	0.001257	./Warning:/./././././12.50	.
2:156435440G>GT	rs1175590351	ALS4_539	GPD2	0.015446	./No/./././././11.55	.
2:156478574A>G	rs530917614	ALS1_497	GPD2	0.000539	./pathogenic/./././././21.4	.
2:156486542T>C	rs1265401970	ALS2_503	GPD2	6.57E-06	./benign/./././././15.64	.
2:156490402C>A	rs990459934	ALS4_539	GPD2	0.021609	./benign/./././././14.37	.
2:156505004TG>T	rs150979484	ALS1_497; ALS5_5608	GPD2	0.006446	./././././././18.57	.
2:169564865C>T	rs150820555	ALS4_539	FASTKD1	0.001845	./benign/./././././10.69	.
2:171318334A>G	rs778098624	ALS3_536	METTL8	0.000217	./benign/./././././12.60	.
2:171342223C>T	rs142698957	ALS8_5611	METTL8	0.004153	./benign/./././././15.29	.
2:171364677A>AAC	rs150877885	ALS1_497	METTL8	0.000844	./././././././11.03	.
2:171420719A>G	rs143080157	ALS3_536	METTL8	0.003601	./benign/./././././12.64	.
2:171844803G>A	rs190065215	ALS4_539	SLC25A12	0.002798	./benign/./././././12.16	.
2:171845382CA>C	rs879467144	ALS1_497	SLC25A12	0.003816	./././././././13.52	.
2:172034159A>G	rs764308989	ALS6_5609	METAP1D	0.000217	./benign/./././././14.33	.
2:172051612T>G	rs760225350	ALS6_5609	METAP1D	5.91E-05	./pathogenic/./././././20.4	.
2:172083299G>A	rs529891603	ALS8_5611	METAP1D	0.002765	./pathogenic/./././././18.73	.
2:172083300C>T	rs549758464	ALS8_5611	METAP1D	0.002765	./pathogenic/./././././19.07	.
2:172083932A>C	rs113835394	ALS8_5611	METAP1D	0.002766	./pathogenic/./././././20.5	.
2:176269658C>A	rs551177944	ALS5_5608	MTX2	0	tolerated/benign/benign/./0.694/0.26/0.997/1.5/T/23.0	.
2:176275241AT>A	rs200488729	ALS4_539	MTX2	0.001537	./././././././13.33	.
2:176297189A>G	rs116078454	ALS4_539	MTX2	0.004791	./pathogenic/./././././14.50	.
2:176316813T>C	rs138082266	ALS8_5611	MTX2	0.006328	./benign/./././././10.36	.
2:176328829C>T	rs146164822	ALS8_5611	MTX2	0.0025	./benign/./././././10.98	.
2:190946396A>G	rs145921383	ALS1_497; ALS7_5610	GLS	0.004066	./benign/./././././12.98	.
2:190965282C>T	rs149993422	ALS2_503	GLS	0.003637	./benign/./././././13.35	.
2:197714037CTT>C	rs1234584171	ALS2_503	AC011997.1	1.31E-05	./././././././13.98	.
2:199951517A>G	rs190851356	ALS4_539	MAIP1	0.002253	./benign/./././././13.13	.
2:200886117T>C	rs561235556	ALS4_539	NIF3L1	0.00557	./benign/./././././11.67	.
2:201067811C>A	rs184374950	ALS6_5609	NDUFB3	0.001951	./benign/./././././10.88	.
2:201250042G>A	rs149077413	ALS7_5610	CASP8	0.003147	./benign/./././././13.79	.
2:206769412T>C	rs1248442679	ALS2_503	FASTKD2	0	./benign/./././././13.25	.
2:210216311T>G	rs189433918	ALS8_5611	ACADL	0.002279	./benign/./././././13.25	.
2:210538114C>A	rs541778789	ALS2_503	CPS1	1.97E-05	./benign/./././././13.03	.

2:210601647T>A	rs148720484	ALS6_5609	CPS1	0.004725	./benign/./././././20.8	.
2:218270141T>A	rs147309598	ALS1_497	PNKD	0.005099	./benign/Likely_benign/./././././18.40	not_provided
2:218270142C>A	rs140903641	ALS1_497	PNKD	0.005097	./benign/Likely_benign/./././././19.35	not_provided
2:218350493T>C	.	ALS7_5610	PNKD	0	./benign/./././././11.16	.
2:218661173G>C	.	ALS1_497	BCS1L	0	deleterious/probably_damaging/pathogenic/./0.999/0.986/1/3.495/D/29.9	.
2:218671266C>CT	rs35556340	ALS1_497; ALS3_536	RNF25	0.002663	./././././././12.74	.
2:218796543G>A	rs920145015	ALS2_503	CYP27A1	1.98E-05	./benign/./././././10.73	.
2:223951094G>A	rs1471120125	ALS4_539	MRPL44	6.57E-06	./benign/./././././10.45	.
2:223965292A>T	rs768455846	ALS2_503	MRPL44	0.000315	./benign/./././././12.55	.
2:227986002A>G	rs180935455	ALS1_497	SPHKAP	0.003188	./benign/./././././10.52	.
2:228078761C>T	rs75112508	ALS1_497	SPHKAP	0.006613	./benign/./././././10.95	.
2:228087235T>C	rs56349157	ALS5_5608	SPHKAP	0.003898	./benign/./././././10.09	.
2:228122053T>G	rs181669173	ALS3_536	SPHKAP	0.001736	./benign/./././././10.78	.
2:240029762A>G	rs61730686	ALS5_5608	NDUFA10	0.005045	./benign/./0.015/0.04/1/2.31/T/15.29	.
2:241735267C>G	rs4675887	ALS4_539	D2HGDH	0.0084	tolerated/benign/benign/Benign/Likely_benign/0/0/1/1.355/T/15.29	D-2-hydroxyglutaric_aciduria_1|not_specified|not_provided
2:241815120G>A	rs116702646	ALS6_5609	NEU4	0.005376	tolerated/benign/benign/./0.282/0.023/0.879/0.92/T/16.99	.
3:3132900A>C	rs939195240	ALS8_5611	TRNT1	6.57E-06	./benign/./././././12.21	.
3:3140565G>A	.	ALS7_5610	TRNT1	0	deleterious/probably_damaging/pathogenic/./0.992/0.901/1/2.715/T/29.4	.
3:9745596G>A	rs775839026	ALS2_503	OGG1	1.97E-05	./pathogenic/./0.997/0.947/1/2.28/T/25.1	.
3:9746037G>C	rs141075845	ALS5_5608	OGG1	0.002254	./benign/./././././10.73	.
3:14132869C>CT	rs1423539317	ALS8_5611	TMEM43	3.29E-05	././Uncertain_significance/./././././32	Cardiomyopathy|Arrhythmogenic_right_ventricular_cardiomyopathy,_type_5|not_provided
3:16293401A>C	rs926651950	ALS5_5608	OXNAD1	2.63E-05	./benign/./././././11.73	.
3:44339202A>G	rs184704981	ALS5_5608	TCAIM	0.002939	./benign/./././././10.78	.
3:45407312G>C	rs148769133	ALS8_5611	LARS2	0.003055	./benign/./././././15.82	.
3:45431711A>AG	rs890428009	ALS4_539	LARS2	0.000171	./././././././11.72	.
3:47853178G>C	rs745990474	ALS2_503	DHX30	0.000164	./pathogenic/./0.994/0.951/1/0.975/T/25.7	.
3:48310223A>T	rs1158963060	ALS4_539	SPINK8	1.31E-05	./benign/./././././10.16	.
3:48428803G>C	rs1207857528	ALS2_503	CCDC51	0.000145	./benign/./././././11.28	.
3:48429110C>T	rs145842830	ALS1_497	CCDC51	0.003771	./benign/./././././12.98	.
3:48433150C>T	rs373488371	ALS6_5609	CCDC51	0.000131	deleterious/probably_damaging/pathogenic/./1/0.987/1/2.625/T/25.6	.
3:48898874C>G	rs551279923	ALS7_5610	SLC25A20	5.91E-05	./benign/Uncertain_significance/./././././17.02	Carnitine_acylcarnitine_translocase_deficiency
3:50567652T>G	.	ALS7_5610	HEMK1	0	./benign/./././././10.63	.

3:50591029G>T	.	ALS4_539	HEMK1	0	./../benign/./././././././12.97	.
3:52199279G>A	rs35338461	ALS4_539	ALAS1	0.003801	tolerated/possibly_damaging/pathogenic/Benign/0.895/0.473/1/2.3/D/24.4	not_provided
3:52282481T>G	rs576751965	ALS3_536	GLYCTK	0.000848	./../benign/./././././././10.38	.
3:52516421G>A	rs201671103	ALS1_497	STAB1	0.00021	tolerated/possibly_damaging/benign/./0.997/0.66/1/1.565/T/22.9	.
3:52520515G>A	rs147953260	ALS1_497	NT5DC2	0.000985	./../pathogenic/Likely_benign/0.979/0.512/1/2.33/D/24.6	not_provided
3:53843065C>A	rs150555921	ALS8_5611	CHDH	0.002862	./../benign/./././././././12.80	.
3:57556300C>T	rs190633425	ALS7_5610	PDE12	0.005248	./../benign/./././././././10.13	.
3:57571523T>C	rs192919441	ALS7_5610	PDE12	0.005238	./../pathogenic/./././././././16.40	.
3:59789620A>G	rs114287574	ALS8_5611	FHIT	0.001734	./../benign/./././././././11.84	.
3:59794176C>T	rs139077383	ALS3_536	FHIT	0.005415	./../benign/./././././././17.87	.
3:59861866GA>G	rs369230614	ALS4_539	FHIT	0.017248	./././././././././././10.62	.
3:59867383A>G	rs769811679	ALS4_539	FHIT	0.000329	./../benign/./././././././13.48	.
3:59878078G>GA	rs767721160	ALS2_503	FHIT	4.6E-05	./././././././././././12.55	.
3:59887896T>C	rs1222460498	ALS8_5611	FHIT	1.31E-05	./../benign/./././././././13.20	.
3:60011227A>T	rs1468912318	ALS4_539	FHIT	1.31E-05	./../pathogenic/./././././././16.17	.
3:60250391T>C	rs138427963	ALS2_503	FHIT	0.000959	./../benign/./././././././10.99	.
3:60284198A>G	rs145602499	ALS5_5608	FHIT	0.002465	./../benign/./././././././16.89	.
3:60316043T>C	rs140683423	ALS1_497	FHIT	0.002621	./../pathogenic/./././././././10.64	.
3:60355328T>C	rs142232005	ALS6_5609	FHIT	0.000992	./../benign/./././././././15.43	.
3:60406519T>C	rs185430740	ALS2_503	FHIT	0.005461	./../pathogenic/./././././././21.3	.
3:60421116A>G	rs757733394	ALS8_5611	FHIT	0.000127	./../benign/./././././././19.47	.
3:60452624T>G	rs1447302941	ALS2_503	FHIT	1.31E-05	./../benign/./././././././16.12	.
3:60452642T>A	rs145290628	ALS6_5609	FHIT	0.001537	./../benign/./././././././10.96	.
3:60543403G>A	rs1234978919	ALS6_5609	FHIT	1.31E-05	./../benign/./././././././11.18	.
3:60568759CAGCTCTTAGTCA>C	rs948881765	ALS2_503	FHIT	7.89E-05	./././././././././././16.07	.
3:60598710G>C	rs9864591	ALS2_503	FHIT	0.026826	./../benign/./././././././13.84	.
3:60598999G>C	rs9868743	ALS2_503	FHIT	0.026832	./../pathogenic/./././././././15.72	.
3:60610497T>C	.	ALS8_5611	FHIT	0	./../benign/./././././././13.72	.
3:60948968A>G	rs73100071	ALS6_5609	FHIT	0.005004	./../benign/./././././././16.41	.
3:60984343A>G	.	ALS7_5610	FHIT	0	./../benign/./././././././13.83	.
3:61031322C>G	rs138747732	ALS5_5608	FHIT	0.004016	./../benign/./././././././12.20	.
3:61129825G>T	rs145066586	ALS2_503	FHIT	0.004666	./../benign/./././././././17.63	.
3:61141516A>AAATCCG	rs1553852573	ALS3_536	FHIT	0.00264	./././././././././././14.60	.
3:61251341G>T	rs114542366	ALS3_536	FHIT	0.004158	./../pathogenic/./././././././14.08	.
3:66124886A>C	rs539393734	ALS5_5608	SLC25A26	0.000519	./../benign/./././././././10.71	.
3:66299969T>C	.	ALS7_5610	SLC25A26	0	./../benign/./././././././13.34	.

3:66300065A>T	rs1033438858	ALS6_5609	SLC25A26	0.00092	././benign/./././././20.0	.
3:66317204C>T	rs756371413	ALS7_5610	SLC25A26	0.00023	././benign/./././././13.09	.
3:66379482A>G	rs545224126	ALS5_5608	SLC25A26	0.00069	././benign/./././././10.12	.
3:67466881C>T	rs775816086	ALS4_539	SUCLG2	0.00023	././benign/./././././11.22	.
3:67630323A>T	rs771652386	ALS4_539	SUCLG2	0.00023	././benign/./././././11.66	.
3:94132250G>A	rs929150015	ALS3_536	NSUN3	1.31E-05	././pathogenic/./././././20.4	.
3:111986079T>C	rs528364656	ALS8_5611	ABHD10	7.9E-05	././benign/./././././14.51	.
3:120587620A>G	rs1480214483	ALS5_5608	NDUFB4	6.57E-06	././pathogenic/./././././19.39	.
3:121436272C>T	rs3218635	ALS1_497	POLQ	0.000335	deleterious/probably_damaging/pathogenic/Uncertain_ significance/1/0.999/1/2.67/D/28.1	not_provided
3:121466859GAT>G	rs1182682163	ALS1_497	POLQ	1.97E-05	./././././././././17.56	.
3:122363244A>C	rs536915182	ALS3_536	CCDC58	0.001209	././benign/./././././11.87	.
3:122414868T>G	rs149616840	ALS7_5610	FAM162A	1.97E-05	././pathogenic/./1/0.999/1/1.985/T/25.5	.
3:126123001T>C	rs151184213	ALS6_5609	ALDH1L1	0.002274	././benign/./././././13.98	.
3:126160912C>T	rs143122118	ALS4_539	ALDH1L1	0.003934	deleterious/probably_damaging/pathogenic/Benign/1/1 /1/2.725/D/25.8	not_provided
3:126171095A>G	rs147639189	ALS1_497	ALDH1L1	0.049269	././benign/./././././10.26	.
3:126704269C>T	rs6804224	ALS1_497	CHCHD6	0.0244	././benign/./././././14.77	.
3:126704270C>A	rs6804225	ALS1_497	CHCHD6	0.0244	././benign/./././././13.38	.
3:126710673T>G	rs184176975	ALS3_536; ALS8_5611	CHCHD6	0.004054	././benign/./././././11.28	.
3:126723891C>T	rs115316240	ALS3_536	CHCHD6	0.004556	././benign/./././././15.39	.
3:126736581AT>A	rs768872534	ALS6_5609	CHCHD6	0.000644	./././././././././11.11	.
3:126762575A>G	rs112444343	ALS8_5611	CHCHD6	6.57E-05	././benign/./././././10.60	.
3:136322892T>C	rs183256104	ALS2_503	PCCB	0.001715	././benign/./././././13.25	.
3:139607837C>T	rs988013236	ALS3_536	NMNAT3	0	././benign/./././././16.66	.
3:158671006C>T	rs115108034	ALS8_5611	GFM1	0.00692	././benign/Benign/0/0/1/1.15/T/13.06	not_provided
3:179624018C>T	rs200889798	ALS6_5609	NDUFB5	0.000131	tolerated/possibly_damaging/benign/./0.097/0.073/0.999 /2.83/T/22.5	.
3:180975427GT>G	rs34318725	ALS3_536; ALS4_539	FXR1	0.001152	././Warning:/./././././15.03	.
3:183027754T>G	rs757603193	ALS8_5611	MCCC1	0.001051	././benign/./././././10.74	.
3:193618721T>TA	rs550849134	ALS4_539	OPA1	0.000368	./././././././././13.19	.
3:193674381T>C	rs574283226	ALS4_539	OPA1	0.00025	././benign/./././././13.09	.
3:197555663C>G	rs147953473	ALS3_536	BDH1	0.006097	././benign/./././././11.98	.
4:2065819CTG>C	rs781062846	ALS5_5608	NAT8L	0.000204	./././././././././15.31	.
4:17577460G>A	rs13123846	ALS6_5609	LAP3	0.000368	././pathogenic/./././././18.84	.
4:42005832A>T	rs188182814	ALS1_497	SLC30A9	0.000801	././benign/./././././16.08	.
4:48842106A>C	rs954310196	ALS8_5611	OCIAD1	6.57E-06	././benign/./././././13.91	.
4:48857303C>T	rs112327139	ALS2_503	OCIAD1	0.003373	deleterious/benign/pathogenic/./0.162/0.045/1/0/T/23.3	.

4:56964489G>A	rs138345362	ALS5_5608	NOA1	0.002243	deleterious/probably_damaging/pathogenic/./1/0.999/1/4.025/T/26.1	.
4:70809218CA>C	rs777261323	ALS1_497; ALS2_503; ALS4_539	MTHFD2L	0.003714	./././././././././13.26	.
4:74230886G>A	rs143361111	ALS8_5611	MTHFD2L	0.004353	./benign/./././././././15.91	.
4:74307302G>A	.	ALS1_497	MTHFD2L	0	./benign/./././././././19.40	.
4:77896963AATTCTTTC>A	rs201963250	ALS5_5608	MRPL1	0.005892	./././././././././14.69	.
4:77923581C>G	rs144348470	ALS6_5609	MRPL1	0.007574	./benign/./././././././12.08	.
4:77949867C>T	rs137874988	ALS1_497	MRPL1	0.000105	deleterious/probably_damaging/benign/./1/0.972/1/2.445/T/25.7	.
4:109605120A>G	rs745996652	ALS3_536	MCUB	0.00046	./benign/./././././././13.84	.
4:122883553G>A	rs189576239	ALS6_5609; ALS7_5610	FGF2	0.003851	./benign/./././././././10.41	.
4:127744218G>T	rs116441649	ALS2_503	SLC25A31	0.005128	./benign/./././././././11.02	.
4:139040927G>A	rs912870142	ALS8_5611	NOCT	0.000132	./benign/./././././././13.14	.
4:139299974G>A	rs144920917	ALS1_497	NDUFC1	0.002662	./benign/./././././././10.97	.
4:151712371C>T	rs77139350	ALS8_5611	GATB	0.003883	./benign/./././././././17.36	.
4:184630850A>G	rs564549833	ALS2_503	CASP3	0.00069	./benign/./././././././10.81	.
4:184756015C>T	rs562295208	ALS2_503	ACSL1	0.00048	./benign/./././././././15.77	.
4:185134437T>C	rs1188756713	ALS2_503	NA	0	./benign/./././././././10.44	.
5:36209771T>C	rs908829844	ALS8_5611	NADK2	6.57E-06	./benign/./././././././12.50	.
5:36231804T>C	rs185791886	ALS7_5610	NADK2	0.000683	./benign/./././././././10.24	.
5:41727118G>A	rs143464951	ALS1_497	OXCT1	0.004004	./benign/./././././././15.04	.
5:41795015C>T	rs144963931	ALS5_5608	OXCT1	0.002063	./benign/./././././././10.87	.
5:41861521A>T	rs141686747	ALS2_503	OXCT1	0.000834	./benign/./././././././17.37	.
5:41875624A>T	rs1184193565	ALS7_5610	OXCT1-AS1	1.31E-05	./benign/./././././././14.58	.
5:43651183G>A	rs184982634	ALS1_497	NNT	0.001363	./benign/./././././././15.12	.
5:44808996C>A	rs112901974	ALS8_5611	MRPS30	0.001859	tolerated/benign/benign/./0.71/0.313/1/2.34/T/16.15	.
5:53690955A>G	.	ALS5_5608	NDUFS4	6.57E-06	./benign/./././././././14.02	.
5:56934376T>C	rs550394008	ALS8_5611	MIER3	0.000243	./benign/./././././././10.22	.
5:61048145A>G	rs751050690	ALS2_503	NDUFAF2	8.55E-05	./benign/./././././././10.73	.
5:65754225T>C	rs16894294	ALS5_5608	NLN	0.00853	./benign/./././././././11.33	.
5:71638343C>T	rs186273368	ALS7_5610	MCCC2	0.004442	./benign/./././././././10.17	.
5:72209520G>GA	rs878901158	ALS2_503	MAP1B	0.001776	./././././././././14.53	.
5:72217396G>A	rs1053508166	ALS5_5608	MRPS27	1.97E-05	./benign/./././././././14.46	.
5:72242428GA>G	rs750987632	ALS4_539	MRPS27	0.001159	./././././././././10.50	.
5:72275097T>C	rs754220252	ALS2_503	MRPS27	0.001314	./benign/./././././././10.56	.
5:78998322T>G	rs185395937	ALS2_503	DMGDH	0.000756	./benign/./././././././10.52	.

5:79013528A>G	rs116702331	ALS8_5611	DMGDH	0.003904	./../benign/./././././././10.79	.
5:79044326C>T	rs139044238	ALS2_503	DMGDH	0.000887	./../benign/Conflicting_interpretations_of_pathogenicity/././1/././40	Dimethylglycine_dehydrogenase_deficiency|not_provided
5:126595278A>C	rs563928852	ALS1_497	ALDH7A1	0.003084	./../benign/Conflicting_interpretations_of_pathogenicity/0.012/0.008/1/./T/17.84	Pyridoxine-dependent_epilepsy|not_provided
5:127061996T>C	rs116162582	ALS4_539	C5orf63	0.000158	./../benign/./././././././14.79	.
5:131208871T>A	rs997591800	ALS7_5610	LYRM7	3.29E-05	./../benign/./././././././15.12	.
5:131997947G>A	rs767394745	ALS8_5611	ACSL6	2.63E-05	./../benign/./././././././16.74	.
5:132015504TA>T	rs771045372	ALS2_503	AC034228.2	0.000887	./././././././././12.35	.
5:133983468A>G	rs74762551	ALS3_536	VDAC1	0.004148	./../benign/./././././././15.17	.
5:133991168A>G	rs766212922	ALS2_503	VDAC1	1.31E-05	./../benign/./././././././16.43	.
5:140657125C>T	rs1461096690	ALS8_5611	IK	1.31E-05	./../benign/./././././././10.64	.
5:141923217A>G	rs558291156	ALS4_539	DELE1	0.000716	./../benign/./././././././12.66	.
5:141924071G>A	rs570254184	ALS6_5609	DELE1	0.000795	./../benign/./././././././14.52	.
5:145753221GGCTGCTATTTTCTCTCTGCT>G	rs562806501	ALS5_5608	PRELID2	0.006962	./././././././././16.26	.
5:145800593G>T	rs768856904	ALS4_539	PRELID2	3.29E-05	./../benign/./././././././15.39	.
5:145801452C>G	rs142521367	ALS3_536	PRELID2	0.002497	./../benign/./././././././11.50	.
5:145835260G>GCCGCGGGCCCCGCGCA	rs749870614	ALS5_5608	PRELID2	0.003803	./././././././././10.60	.
5:154938231G>A	rs983356481	ALS1_497	MRPL22	7.88E-05	./../benign/./././././././15.48	.
5:177302011C>T	rs376678813	ALS4_539	RAB24	7.23E-05	./../benign/./././././././15.18	.
6:3152060C>G	rs569185118	ALS8_5611	BPHL	0.000769	./../benign/./././././././11.05	.
6:5564222G>A	rs192624970	ALS1_497	FARS2	0.002478	./../benign/./././././././11.18	.
6:5631272A>G	.	ALS4_539	FARS2	0	./../benign/./././././././15.77	.
6:13615497G>A	.	ALS8_5611	SIRT5	0	./../benign/./0.778/0.083/0.554/1.1/T/24.7	.
6:30617265A>G	rs533548299	ALS6_5609	MRPS18B	0.00224	./../benign/./././././././19.25	.
6:30617305G>A	rs528193181	ALS2_503	MRPS18B	0.002365	./../pathogenic/./././././././19.20	.
6:30637367C>CT	rs769594150	ALS1_497	ATAT1	0.003905	./././././././././10.96	.
6:30644142A>G	.	ALS6_5609	C6orf136	0	./../benign/./././././././21.2	.
6:33688111CAGG>C	rs757436464	ALS6_5609	ITPR3	0.001091	./././././././././18.88	.
6:33697267G>T	rs779010521	ALS7_5610	UQCC2	5.91E-05	./../benign/./././././././15.50	.
6:39897231T>C	rs61748650	ALS8_5611	MOCS1	0.003963	./../pathogenic/./0.544/0.343/1/1.485/T/22.6	.
6:42225005A>G	rs190167403	ALS8_5611	TRERF1	0.005086	./../benign/./././././././15.04	.
6:44313205G>C	rs778971317	ALS8_5611	AARS2	6.57E-06	deleterious/benign/benign/./0.953/0.366/1/0/T/22.3	.
6:46653111G>A	rs906344645	ALS7_5610	SLC25A27	2.63E-05	./../benign/./././././././10.51	.

6:49472903A>G	rs751354871	ALS3_536	CENPQ	0	./../benign/./././././.10.88	.
6:73452132G>C	rs147035222	ALS1_497	CGAS	0.00446	tolerated/benign/benign/Likely_benign/0.014/0.004/1/1.67/T/10.48	not_provided
6:80152041T>C	rs17808284	ALS4_539	BCKDHB	0.004783	./../pathogenic/./././././.19.54	.
6:80180347A>G	rs780633715	ALS2_503	BCKDHB	3.28E-05	./../benign/./././././.10.60	.
6:80299529C>G	rs543075053	ALS2_503	BCKDHB	0.000184	./../benign/./././././.10.51	.
6:80327325C>T	rs559991659	ALS1_497	BCKDHB	0.001117	./../benign/./././././.10.97	.
6:80346019A>AT	rs757446051	ALS1_497	BCKDHB	0.001404	./././Uncertain_significance/./././././10.98	Maple_syrup_urine_disease
6:80350779A>C	rs146824244	ALS5_5608	BCKDHB	0.007176	./../benign/./././././.10.16	.
6:87343950T>G	rs1239137380	ALS1_497	SMIM8	1.31E-05	./../benign/./././././.10.91	.
6:87513967G>T	rs139134669	ALS4_539;ALS7_5610	RARS2	0.005988	./../benign/./././././.10.23	.
6:89623997T>A	.	ALS7_5610	LYRM2	0.004519	./../benign/./0.973/0.942/0.999/2.215/T/13.56	.
6:96886859T>G	rs182505265	ALS5_5608	NDUFAF4	0.001715	./../benign/./././././.13.26	.
6:98917495A>G	rs143154211	ALS6_5609	FBXL4	0.000723	tolerated/benign/pathogenic/Uncertain_significance/0.004/0.073/0.989/0.69/T/16.79	Mitochondrial_DNA_depletion_syndrome_13_(encephalomyopathic_type)|not_provided
6:99378948CT>C	rs144759090	ALS1_497	COQ3	0.001824	./././././././././.10.82	.
6:99378948CTT>C	rs144759090	ALS3_536	COQ3	0.025838	./././././././././.10.82	.
6:106575931T>A	rs557628780	ALS2_503	RTN4IP1	0.00027	./../benign/./././././.10.61	.
6:107177862G>A	rs1426407103	ALS5_5608	PDSS2	0	./../benign/./././././.16.25	.
6:107201390CA>C	rs747612959	ALS4_539	PDSS2	0.018184	./././././././././.12.52	.
6:107239395AATT>A	rs557244043	ALS1_497	PDSS2	0.002286	./././././././././.15.07	.
6:107365710T>C	rs574613432	ALS7_5610	PDSS2	0.000756	./../benign/./././././.12.37	.
6:107416562G>C	.	ALS4_539	PDSS2	0	./../benign/./././././.10.77	.
6:107427560A>G	rs187176999	ALS6_5609	PDSS2	0.005833	./../benign/./././././.14.43	.
6:108326540A>G	rs770968102	ALS4_539	AFG1L	0.000591	./../benign/./././././.14.07	.
6:108340896T>A	rs546059523	ALS6_5609	AFG1L	0.001439	./../benign/./././././.10.72	.
6:108363868A>G	rs551257933	ALS5_5608	AFG1L	0.000637	./../pathogenic/./././././.22.0	.
6:108377818CT>C	rs199511475	ALS1_497	AFG1L	0.011786	./././././././././.11.96	.
6:108495721A>G	rs767102878	ALS4_539	AFG1L	0.000578	./../benign/./././././.17.81	.
6:108507884C>T	rs533731425	ALS1_497	AFG1L	0.000125	./../benign/./././././.11.67	.
6:127284727T>G	rs184496482	ALS3_536	ECHDC1	0.000572	./../benign/./././././.11.55	.
6:127337490C>G	rs776241829	ALS8_5611	ECHDC1	7.23E-05	./../benign/./././././.10.82	.
6:150994945A>G	rs1207556353	ALS3_536	MTHFD1L	1.31E-05	./../benign/./././././.12.98	.
6:151042587G>T	rs575020062	ALS2_503; ALS8_5611	MTHFD1L	0.000421	./../benign/./././././.16.66	.
6:151452445G>C	rs756319585	ALS8_5611	RMND1	1.97E-05	./../benign/./././././.10.06	.
6:161642098A>G	rs546252361	ALS6_5609	PRKN	0.00025	./../benign/./././././.12.25	.
6:162648998A>G	rs529518802	ALS7_5610	PRKN	0.001459	./../benign/./././././.12.95	.

7:23299353A>G	rs200950879	ALS4_539	MALSU1	0.000493	deleterious/benign/benign/./0.231/0.051/0.999/./T/18.80	.
7:27517685A>AT	rs200032035	ALS3_536	HIBADH	0.042601	./././././././././10.11	.
7:27541591T>C	rs572713424	ALS7_5610	HIBADH	0.001163	././benign/./././././12.34	.
7:27556307T>G	rs551345110	ALS8_5611	HIBADH	0.000191	././benign/./././././12.86	.
7:27662588A>T	rs966917581	ALS2_503	HIBADH	4.6E-05	././benign/./././././11.57	.
7:40300374A>G	rs186425207	ALS3_536	SUGCT	0.000283	././benign/./././././10.63	.
7:40348277C>G	rs772809878	ALS3_536	SUGCT	8.54E-05	././benign/./././././13.46	.
7:40379144A>G	rs767110809	ALS5_5608	SUGCT	0.000736	././benign/./././././12.34	.
7:40530559C>A	rs191253702	ALS7_5610	SUGCT	0.003142	././benign/./././././15.34	.
7:40721879A>G	rs139915353	ALS1_497	SUGCT	0.002707	././benign/./././././13.29	.
7:40762228T>C	rs540139546	ALS6_5609	SUGCT	0.000374	././benign/./././././15.89	.
7:40813970G>A	rs573239511	ALS5_5608	SUGCT	0.000598	././benign/./././././14.50	.
7:42935589A>G	.	ALS8_5611	MRPL32	0	././benign/./././././12.83	.
7:43859793C>G	rs187912470	ALS8_5611	MRPS24	3.29E-05	././benign/./././././13.15	.
7:75021908G>A	rs942662977	ALS6_5609	CASTOR2	0.002352	deleterious/probably_damaging/benign/./1/1/0.996/./T/26.4	.
7:95588230T>C	.	ALS6_5609	PDK4	0	././benign/./././././16.87	.
7:96118628GA>G	rs78530901	ALS4_539	SLC25A13	0.00318	./././././././././10.03	.
7:96174671C>T	rs189529761	ALS7_5610	SLC25A13	0.001511	././benign/./././././14.27	.
7:99407238T>G	rs185197449	ALS5_5608	PDAP1	0.005722	././benign/./././././15.03	.
7:102799451A>G	rs765437889	ALS1_497	FAM185A	5.91E-05	././pathogenic/./././././10.88	.
7:103067392T>C	rs112939502	ALS8_5611	FBXL13	0.005091	././benign/./././././12.52	.
7:103099796CT>C	rs546919510	ALS4_539	ARMC10	0.001358	./././././././././10.36	.
7:107899984T>A	rs1330646280	ALS2_503	DLD	0.000539	././benign/./././././13.58	.
7:108514933G>A	rs139626312	ALS5_5608	PNPLA8	0.002971	deleterious/benign/benign/Conflicting_interpretations_of_pathogenicity/0.9/0.316/1/0.345/D/22.6	Mitochondrial_myopathy-lactic_acidosis-deafness_syndrome|not_provided
7:108526309A>G	rs571574461	ALS2_503	PNPLA8	0.001375	././benign/./././././11.62	.
7:110724979G>A	rs112072300	ALS8_5611	IMMP2L	0.004619	././pathogenic/./././././21.2	.
7:110781410CTTTT>C	rs549941484	ALS2_503	IMMP2L	0.005663	./././././././././10.55	.
7:110786678C>T	rs189599734	ALS1_497	IMMP2L	0.001659	././benign/./././././10.55	.
7:110888464A>G	rs536032055	ALS3_536	IMMP2L	0.002168	././benign/./././././11.04	.
7:110963539ATCT>A	rs780790549	ALS3_536	IMMP2L	1.32E-05	./././././././././20.8	.
7:111171362T>C	rs571823194	ALS2_503	IMMP2L	0.002894	././benign/./././././10.95	.
7:111304069C>T	rs557153940	ALS8_5611	IMMP2L	0.002368	././benign/./././././13.92	.
7:111349229T>G	rs193092417	ALS1_497	IMMP2L	0.002005	././benign/./././././12.52	.

7:111413698A>ATGATATGGCTGTTTCC	rs1180257335	ALS6_5609	IMMP2L	0.00193	./././././././././12.05	.
7:111413700G>GAGACTATAGTGGATGGTCATAGTTCTTTATTGGGACTTC	rs1274795952	ALS6_5609	IMMP2L	0.000807	./././././././././10.45	.
7:111424999TAGTCATTACATGA>T	rs537695206	ALS5_5608	IMMP2L	0.000184	./././././././././12.04	.
7:122089949G>A	rs1041107168	ALS8_5611	AASS	5.26E-05	././benign/./././././12.48	.
7:127591374C>T	rs192153447	ALS6_5609	ARF5	0.000315	././pathogenic/./././././15.78	.
7:127595331G>A	rs150511464	ALS5_5608	ARF5	0.000841	././benign/./0.998/0.947/1/2.28/T/23.3	.
7:127816222T>C	rs112156758	ALS3_536	SND1	0.000552	././benign/./././././11.66	.
7:127845068T>A	.	ALS5_5608	SND1	0	././benign/./././././10.61	.
7:127860092G>T	rs188923418	ALS6_5609	SND1	0.000644	././benign/./././././20.5	.
7:127882871A>G	rs555946924	ALS5_5608	SND1	0.00092	././benign/./././././16.54	.
7:127940507C>T	rs187512631	ALS2_503	SND1	0.005001	././benign/./././././15.12	.
7:128007844A>G	rs577667784	ALS1_497	SND1	9.85E-05	././benign/./././././18.36	.
7:128027686C>T	rs147853002	ALS7_5610	SND1	0.004593	././benign/./././././16.89	.
7:128028381A>G	rs555511715	ALS6_5609	SND1	0.000158	././pathogenic/./././././16.75	.
7:128032205CGCCGGCGCCTTCCAGCGCCGCGCCG>C	rs1472981829	ALS6_5609	SND1	0.000686	./././././././././18.41	.
7:128042174G>A	.	ALS6_5609	SND1	0	././pathogenic/./././././20.7	.
7:128075844C>A	rs1044295059	ALS4_539	SND1	2.63E-05	././benign/./././././10.92	.
7:132798021T>C	rs1160432075	ALS3_536	CHCHD3	6.57E-06	././benign/./././././14.44	.
7:132798288A>T	rs139333910	ALS3_536	CHCHD3	0.002332	././pathogenic/./././././18.76	.
7:132798304A>G	.	ALS6_5609	CHCHD3	0	././benign/./././././13.79	.
7:132876156A>G	rs138496938	ALS7_5610	CHCHD3	0.003318	././benign/./././././18.12	.
7:132923864G>C	rs1341558471	ALS3_536	CHCHD3	6.57E-06	././benign/./././././12.50	.
7:132971786C>T	rs150816250	ALS5_5608	CHCHD3	0.005981	././benign/./././././10.55	.
7:133007747T>C	rs143878940	ALS7_5610	CHCHD3	0.003331	././benign/./././././17.72	.
7:133008425GA>G	rs988855864	ALS1_497; ALS2_503	CHCHD3	0.001919	./././././././././10.02	.
7:133035857C>T	rs571009566	ALS2_503	CHCHD3	0.000716	././pathogenic/./././././12.16	.
7:141636963C>A	rs771945804	ALS2_503	AGK	0	././benign/Pathogenic/././1/././37	not_provided
7:141748982A>G	rs1299536412	ALS2_503	SSBP1	0	././benign/./././././10.31	.
7:151068211C>T	rs567715989	ALS6_5609	SLC4A2	0.000585	././benign/./././././12.12	.
8:10095244G>A	rs182405042	ALS4_539	MSRA	0.003228	././benign/./././././16.90	.
8:10125004T>G	rs936657190	ALS8_5611	MSRA	3.94E-05	././benign/./././././11.58	.
8:10142677C>T	rs568303957	ALS3_536	MSRA	0.000191	././benign/./././././10.10	.

8:10167169C>G	rs75317853	ALS2_503; ALS4_539;	MSRA	0.003785	./../benign/./././././../16.14	.
8:10185181C>A	rs139050473	ALS6_5609	MSRA	0.002592	./../pathogenic/./././././../13.25	.
8:10246985A>T	rs191223351	ALS2_503; ALS4_539;	MSRA	0.003857	./../benign/./././././../14.67	.
8:10275062A>G	rs144600842	ALS2_503; ALS4_539;	MSRA	0.005009	./../benign/./././././../11.69	.
8:10281800C>A	rs116860713	ALS2_503; ALS4_539;	MSRA	0.007734	./../benign/./././././../13.27	.
8:10291612A>G	rs773931706	ALS4_539	MSRA	1.31E-05	./../benign/./././././../17.60	.
8:10299511A>G	rs117445903	ALS2_503; ALS4_539;	MSRA	0.005226	./../benign/./././././../13.71	.
8:10302187A>G	rs114677739	ALS2_503; ALS4_539;	MSRA	0.006746	./../benign/./././././../15.64	.
8:10310102T>A	rs142936623	ALS2_503; ALS4_539;	MSRA	0.005774	./../pathogenic/./././././../16.27	.
8:10361043T>C	rs368404603	ALS1_497	MSRA	0.001241	./../benign/./././././../14.02	.
8:17027610C>T	rs200500624	ALS3_536	MICU3	0.00539	tolerated/benign/benign/./0.093/0.029/1/0.55/T/12.01	.
8:17068197A>G	rs562110617	ALS8_5611	MICU3	0.000237	./../benign/./././././../11.59	.
8:23572231AAAAAAAAAAAAAAAAAT>A	rs1219172223	ALS3_536	SLC25A37	0.000842	./././././././././../10.46	.
8:26383076G>T	rs572496113	ALS8_5611	BNIP3L	7.89E-05	./../benign/./././././../20.6	.
8:26386009G>A	rs562664239	ALS2_503	BNIP3L	9.2E-05	./../benign/./././././../19.74	.
8:27491109G>C	rs72473929	ALS5_5608	EPHX2	0.001005	./../benign/./././././../12.66	.
8:30677094A>C	rs932806479	ALS2_503	GSR	1.97E-05	./../benign/./././././../15.14	.
8:37763474G>T	rs182737918	ALS1_497	PLPBP	0.005171	./../benign/./././././../12.68	.
8:54142706T>C	rs146739374	ALS2_503	MRPL15	0.000709	tolerated/benign/pathogenic/./0.002/0.016/0.96/-0.295/T/22.4	.
8:54157269A>G	rs144453766	ALS2_503	RNU6ATAC32P	0.005047	./../benign/./././././../11.19	.
8:65718158C>G	rs118128137	ALS8_5611	MTFR1	0.004829	./../pathogenic/./././././../16.82	.
8:66430093G>A	rs34974332	ALS6_5609	ADHFE1	0.000933	./../benign/./0.004/0.006/1/1.83/D/21.0	.
8:70642042C>T	rs552289232	ALS8_5611	LACTB2	0.000999	./../benign/./././././../11.12	.
8:70644217TCAGTGTGGC>T	rs561918644	ALS8_5611	LACTB2	0.003523	./././././././././../21.8	.
8:73971874C>A	rs186794408	ALS5_5608	TMEM70	0.002945	./../benign/./././././../12.04	.
8:79931048A>T	rs948304431	ALS3_536	MRPS28	3.94E-05	./../benign/./././././../10.04	.
8:79950699T>C	rs116995073	ALS5_5608	MRPS28	0.005241	./../benign/./././././../11.21	.
8:86496044A>G	rs562605558	ALS1_497	RMDN1	0.000368	./../benign/./././././../11.12	.
8:93917735A>C	rs1377008043	ALS4_539	PDP1	0.000874	./../benign/./././././../16.06	.
8:95025091G>C	rs201223057	ALS3_536	NDUFAF6	0.002089	tolerated/benign/benign/Likely_benign/0.088/0.034/1/0.69/T/10.52	not_provided

8:96234823G>A	rs1473636960	ALS2_503	UQCRB	0	./../benign/./././././././17.19	.
8:96250935G>C	.	ALS4_539	MTERF3	0	tolerated/benign/benign/./0.006/0.005/0.966/0.41/T/13.93	.
8:106332021T>C	rs534692339	ALS2_503	OXR1	0.001003	./../benign/./././././././14.67	.
8:106337072A>G	rs187360751	ALS7_5610	OXR1	0.004762	./../benign/./././././././15.47	.
8:106358275A>G	rs562953453	ALS6_5609	OXR1	0.001039	./../benign/./././././././11.46	.
8:106414890T>C	rs1009583516	ALS2_503	OXR1	1.97E-05	./../benign/./././././././20.4	.
8:106455579A>G	rs536247877	ALS5_5608	OXR1	0.000552	./../benign/./././././././13.69	.
8:106679718A>G	rs181430419	ALS4_539	OXR1	0.002648	./../benign/./././././././12.19	.
8:106739321T>C	rs3739323	ALS8_5611	OXR1	0.016118	./../benign/./././././././11.03	.
8:120389801C>A	rs144906584	ALS2_503	MRPL13	0.003401	./../benign/./././././././13.93	.
8:120450065T>A	rs62528319	ALS5_5608	MRPL13	0.002394	./../benign/./././././././11.43	.
8:124350070A>C	rs115607174	ALS3_536	TMEM65	0.028676	./../benign/./././././././10.35	.
8:143299423C>T	rs531354684	ALS3_536	TOP1MT	0.000664	./../benign/./././././././14.58	.
8:144385963CA>C	rs879964077	ALS2_503	ADCK5	0.002394	./././././././././././11.45	.
8:144385963C>CA	rs879964077	ALS3_536	ADCK5	0.001423	./././././././././././10.81	.
8:144395469G>A	rs782156961	ALS7_5610	ADCK5	1.97E-05	./../pathogenic/./0.981/0.768/1/2.94/D/24.4	.
8:144399650G>A	.	ALS7_5610	CPSF1	0	deleterious/probably_damaging/pathogenic/./0.997/0.967/1/1.655/T/26.2	.
8:144502615G>C	rs1053742811	ALS2_503	PPP1R16A	1.32E-05	./../benign/./././././././13.16	.
8:144529620G>A	rs371288035	ALS8_5611	C8orf82	0.001321	./../benign/./././././././16.29	.
8:144529788T>A	.	ALS8_5611	C8orf82	0.002328	./../benign/./././././././14.10	.
9:6553028T>C	rs777466122	ALS8_5611	GLDC	9.21E-05	./../benign/./././././././12.02	.
9:6611238G>C	rs193049097	ALS8_5611	GLDC	0.000815	./../benign/./././././././11.64	.
9:6625683T>A	.	ALS4_539	GLDC	0	./../benign/./././././././11.33	.
9:69064224C>G	rs761070483	ALS8_5611	FXN	4.6E-05	./../benign/./././././././13.85	.
9:76394542A>AGCCCCGCCCCATCATGGCCCCGCCCCATCATG	rs538738583	ALS8_5611	RFK	0.001947	./././././././././././18.43	.
9:99906588CGGGGCGGGGGCG>C	rs746882663	ALS2_503	STX17	0.004865	./././././././././././10.50	.
9:99917473G>A	rs182341907	ALS5_5608	STX17	0.002248	./../benign/./././././././17.05	.
9:99967591T>A	rs117850621	ALS6_5609	STX17	0.005697	./../pathogenic/./././././././18.15	.
9:104739806T>C	rs763561001	ALS1_497	NIPSNAP3A	5.26E-05	./../benign/./././././././13.61	.
9:121336783C>T	rs182519055	ALS5_5608	STOM	0.006547	./../benign/./././././././10.15	.
9:122271603C>A	rs12376093	ALS1_497; ALS3_536	MRRF	0.003397	./../benign/./././././././16.34	.
9:122281288T>G	rs139673690	ALS7_5610	MRRF	0.004441	./../pathogenic/./././././././16.85	.
9:122281988G>C	rs965893026	ALS2_503	MRRF	3.29E-05	./../benign/./././././././15.28	.
9:122291452G>T	rs140077839	ALS6_5609	MRRF	0.000191	./../pathogenic/./././././././20.3	.

9:127798462T>C	.	ALS3_536	FPGS	0	./benign/./././././12.30	.
9:128101311C>T	rs35473740	ALS1_497	SLC25A25	0.000151	tolerated/benign/benign/./0.022/0.022/1/1.615/T/22.0	.
9:129104214G>C	rs148243375	ALS6_5609	CRAT	0.001045	tolerated/benign/pathogenic/Benign/0/0.001/0.621/0.945/T/17.04	not_provided
9:133350074G>A	rs41313873	ALS6_5609	SURF1	0.007044	./benign/./././././10.02	.
9:133360052G>A	rs62637581	ALS2_503	SURF2	0.000269	tolerated/benign/benign/./0.795/0.113/1/2.08/T/11.13	.
9:133362377G>A	rs139684139	ALS2_503	SURF4	0.003726	./benign/./././././12.42	.
9:133729791C>T	rs137999690	ALS2_503	SARDH	0.00094	tolerated/possibly_damaging/pathogenic/./0.948/0.591/1/1.78/D/21.6	.
9:137552502G>C	rs4551	ALS5_5608	MRPL41	0.003475	./benign/./././././10.91	.
10:12129703G>A	rs531245228	ALS7_5610	SEC61A2	0.002183	./benign/./././././12.40	.
10:26697752G>T	rs1337919886	ALS1_497	PDSS1	6.58E-06	deleterious/benign/benign/./0.002/0.003/0.607/1.845/T/23.9	.
10:26710156A>T	rs1370374849	ALS1_497	PDSS1	0.000206	./benign/./././././10.11	.
10:26746373A>G	rs770190130	ALS6_5609	PDSS1	1.31E-05	deleterious/probably_damaging/pathogenic/./1/0.996/1/2.645/D/32	.
10:26755327T>TA	rs1338584730	ALS4_539	ABI1	2.63E-05	./././././././././13.17	.
10:46006369A>G	rs113983044	ALS6_5609	TIMM23	0.007699	./benign/./././././15.51	.
10:46010581T>C	rs61754798	ALS8_5611	NCOA4	0.003541	tolerated/benign/benign/not_provided/./././././19.78	not_provided
10:49739779A>G	rs143105288	ALS7_5610	OGDHL	0.006263	deleterious/probably_damaging/pathogenic/Likely_benign/0.996/0.954/1/4.385/D/31	Inborn_genetic_diseases
10:58272225TA>T	rs11435069	ALS2_503	CISD1	0.001373	./././././././././10.51	.
10:58394263G>A	rs191159394	ALS8_5611	TFAM	4.6E-05	./benign/./././././11.68	.
10:68454356CA>C	rs562333641	ALS3_536	DNA2	0.062868	./././././././././11.82	.
10:68491046T>A	rs139555638	ALS6_5609	SLC25A16	0.001862	./benign/./././././10.18	.
10:70124080C>A	rs41277978	ALS3_536	AIFM2	0.004099	deleterious/probably_damaging/pathogenic/./1/0.999/1/1.995/T/23.6	.
10:72721357A>T	rs761605865	ALS8_5611	MCU	0.000151	./benign/./././././12.46	.
10:72812965T>C	.	ALS5_5608	MCU	0	./benign/./././././17.08	.
10:72853203T>C	rs147565386	ALS6_5609	MCU	0.000842	./benign/./././././11.39	.
10:73122286T>G	rs149066778	ALS6_5609	NUDT13	0.002115	deleterious/possibly_damaging/benign/./0.985/0.706/0.88/2.52/T/25.3	.
10:73785808C>T	rs536569732	ALS4_539	CHCHD1	0.00094	./benign/./././././19.95	.
10:80414331C>T	rs765873836	ALS5_5608	PRXL2A	9.2E-05	./benign/./././././12.92	.
10:84140097C>T	rs1469461250	ALS4_539	GHITM	6.57E-06	./benign/./././././12.46	.
10:87801289T>C	rs186947620	ALS4_539	ATAD1	0.006373	./benign/./././././14.73	.
10:87818279A>G	rs41299161	ALS4_539	ATAD1	0.006144	./benign/./././././15.71	.
10:92472642CTT>C	rs35959170	ALS2_503; ALS4_539	IDE	0.000763	./././././././././14.75	.
10:92472642C>CT	rs35959170	ALS5_5608	IDE	0.006283	./././././././././15.57	.

10:92521398TA>T	rs879425676	ALS1_497; ALS4_539	IDE	0.001898	./././././././././10.14	.
10:97578505C>T	rs36020819	ALS6_5609	ANKRD2	0.004587	deleterious/benign/pathogenic/Benign/0.96/0.711/1/1.04/T/24.1	not_provided
10:99614939A>G	rs117154413	ALS6_5609	SLC25A28	0.00701	./../benign/./././././14.54	.
10:99706982T>C	rs774468091	ALS3_536	COX15	0.000282	./../benign/./././././14.59	.
10:99729694C>T	rs141506146	ALS6_5609	COX15	0.000112	deleterious/benign/benign/Uncertain_significance/0.001/0.003/0.567/0.895/T/23.4	Leigh_syndrome
10:100514556T>TA	rs1193831818	ALS2_503	SEC31B	0.010723	./././././././././11.62	.
10:101032325C>T	rs185856010	ALS4_539	SFXN3	0.000815	./../benign/./././././15.25	.
10:103396597G>A	rs762283604	ALS3_536	ATP5MD	0.000177	./../benign/./././././10.22	.
10:103398247A>G	rs76145370	ALS3_536	ATP5MD	0.004105	./../benign/./././././10.10	.
10:112151477T>C	rs144051314	ALS7_5610	GPAM	0.006332	./../pathogenic/./././././15.80	.
10:112152824G>A	rs144174877	ALS3_536	GPAM	0.003699	./../benign/./././././12.13	.
10:123000628T>C	rs940170030	ALS6_5609	IKZF5	0.000486	./../benign/./././././10.65	.
10:123051188TA>T	rs10571424	ALS1_497	ACADSB	0.032543	./././././././././10.14	.
10:124420383C>T	.	ALS5_5608	OAT	0	./../benign/./././././10.43	.
10:133388960G>GGTCA	rs1459322189	ALS7_5610	PAOX	2.63E-05	./././././././././23.6	.
10:133407970G>A	rs150538191	ALS2_503	MTG1	0.007253	./../benign/./././././11.61	.
10:133423524G>A	rs1351288595	ALS2_503	MTG1	1.34E-05	./../benign/./0.354/0.041/0.764/2.39/T/22.9	.
11:6474177C>G	rs925982476	ALS5_5608	TRIM3	2.63E-05	./../benign/./././././11.27	.
11:6488086T>C	rs192750116	ALS4_539	TIMM10B	0.001774	./../benign/./././././10.04	.
11:20366039A>C	rs543506625	ALS8_5611	HTATIP2	0.000201	./../benign/./././././10.77	.
11:28110143G>A	rs35686304	ALS7_5610	METTL15	0.002036	./../benign/./././././12.79	.
11:31449125A>T	.	ALS1_497	IMMP1L	0	./../benign/./././././15.63	.
11:34919550C>T	rs1215715731	ALS3_536	PDHX	6.57E-06	./../benign/./././././13.77	.
11:34926910T>G	rs528331306	ALS1_497	PDHX	0.003616	./../benign/./././././11.11	.
11:59810778C>T	rs970272411	ALS7_5610	MRPL16	0	./../benign/./././././11.31	.
11:64072034C>T	rs9783361	ALS3_536	MACROD1	0.011985	./../benign/./././././13.83	.
11:64293146G>T	rs150371580	ALS8_5611	KCNK4	0.000585	tolerated/benign/benign/./0.021/0.024/0.986/1.15/T/21.1	.
11:65128334G>A	.	ALS1_497	MRPL49	0	./../benign/./././././13.94	.
11:65132603C>G	rs763493149	ALS1_497	SYVN1	1.31E-05	./../benign/./././././14.68	.
11:65386664G>C	.	ALS8_5611	SLC25A45	6.57E-06	./../benign/./././././10.41	.
11:66426135G>A	.	ALS1_497	NPAS4	0	./../benign/./././././14.04	.
11:66858373C>T	rs537084771	ALS4_539	PC	6.57E-05	./../No/./1/1/1/2.695/T/23.6	.
11:66953162CAAG>C	rs535692776	ALS6_5609	PC	0.003679	./././././././././11.05	.
11:66961586A>T	.	ALS3_536	PC	0	./../benign/./././././16.73	.
11:68815058C>T	rs560794902	ALS5_5608	CPT1A	0.001078	./../benign/./././././11.39	.

11:68908213G>T	.	ALS8_5611	MRPL21	0	./../benign/./0.129/0.043/0.995/2.265/T/14.26	.
11:72372993C>T	rs143097446	ALS8_5611	CLPB	0.00445	tolerated/benign/benign/Benign/Likely_benign/0.019/0.011/1/0.69/T/17.59	3-methylglutaconic_aciduria_with_cataracts,_neurologic_involvement,_and_neutropenia|not_specified|not_provided
11:72422923C>T	rs184738178	ALS5_5608	CLPB	0.003824	./../benign/./././././././14.29	.
11:72580315C>T	rs143118302	ALS2_503; ALS6_5609	PDE2A	0.004261	./../benign/./././././././10.66	.
11:72584037G>A	rs956157527	ALS6_5609	PDE2A	0.000151	./../benign/./././././././14.20	.
11:72601407G>A	rs1482066676	ALS7_5610	PDE2A	1.6E-05	./../benign/./././././././12.24	.
11:73964917T>G	rs139317756	ALS6_5609	DNAJB13	0.001685	deleterious/benign/pathogenic/Likely_benign/0.03/0.055/1/3.4/T/24.5	not_provided
11:73970199G>C	rs188177227	ALS7_5610	UCP2	0.001366	./../benign/./././././././10.56	.
11:78075459C>T	rs117547352	ALS6_5609	NDUFC2	0.005197	./../benign/./././././././12.77	.
11:78466153C>T	rs765066308	ALS4_539	NARS2	6.57E-05	./../benign/./././././././14.90	.
11:78526339G>A	.	ALS2_503	NARS2	0	./../benign/./././././././17.32	.
11:78562134T>C	rs148993906	ALS6_5609	NARS2	0.002294	./../benign/./././././././13.75	.
11:78571419T>C	rs201751992	ALS2_503	NARS2	4.62E-05	tolerated/probably_damaging/pathogenic/Conflicting_interpretations_of_pathogenicity/1/0.986/1/3.49/T/24.3	Combined_oxidative_phosphorylation_deficiency_24|not_provided
11:83285711G>A	rs536788844	ALS7_5610	CCDC90B	0.000112	./../benign/./././././././10.24	.
11:83286535A>G	rs75113853	ALS7_5610	CCDC90B	0.005298	./../benign/./././././././11.44	.
11:85631991T>G	rs528300437	ALS3_536	TMEM126B	0.002765	./../benign/./././././././12.02	.
11:86524653T>G	rs139524923	ALS8_5611	ME3	0.005736	./../benign/./././././././15.34	.
11:110456995A>G	rs147778113	ALS4_539	FDX1	0.00065	tolerated/benign/benign/./0.005/0.005/0.978/0.69/T/11.16	.
11:112014888C>CT	rs200874690	ALS3_536	DIXDC1	0.001275	./././././././././././13.70	.
11:112170498A>G	rs113640749	ALS5_5608	BCO2	0.004047	./../benign/./0.003/0.004/0.897/0.695/T/15.44	.
11:112224960A>C	rs7945257	ALS8_5611	BCO2	0.072339	./../benign/./././././././12.63	.
11:119188823C>T	rs146107413	ALS6_5609	NLRX1	0.000434	./../benign/./././1/././38	.
11:126285162T>C	rs150070873	ALS3_536	TIRAP	0.006439	./../benign/./././././././10.53	.
12:6492453G>A	rs141891222	ALS5_5608	MRPL51	0.006015	deleterious/probably_damaging/pathogenic/./1/0.998/1/2.75/T/31	.
12:6963603G>A	rs745889249	ALS7_5610	PHB2	0.001725	./../benign/./././././././12.96	.
12:6977972A>C	rs782301379	ALS7_5610	EMG1	0.000782	./../benign/./././././././10.37	.
12:32705995T>C	rs553601095	ALS2_503	DNM1L	0.00021	./../pathogenic/./././././././17.57	.
12:39629500G>T	rs140979273	ALS5_5608	C12orf40	0.005733	./../benign/./././././././11.89	.
12:50123671C>CT	rs113279297	ALS3_536	COX14	0.029875	./././././././././././10.82	.
12:55715531G>C	rs144234513	ALS5_5608; ALS6_5609	BLOC1S1	0.004762	./../benign/./././././././16.23	.

12:56002556CCCT>C	rs765291957	ALS2_503	SUOX	1.31E-05	./././././././././12.97	.
12:56477848G>C	rs12819483	ALS6_5609	SPRYD4	0.004061	././pathogenic/./././././././13.05	.
12:57235379G>T	rs1032051308	ALS2_503	SHMT2	6.59E-06	././benign/./././././././17.58	.
12:57241598T>C	rs541722468	ALS5_5608; ALS6_5609	SHMT2	0.002352	././benign/./././././././19.01	.
12:57241873GC>G	rs961228457	ALS8_5611	SHMT2	0.000303	./././././././././13.19	.
12:57755023CATCCTTCA>C	rs1595113302	ALS5_5608	MARCH9	0.000838	./././././././././15.21	.
12:57755034CGCT>C	rs1394647227	ALS5_5608	MARCH9	0.000465	./././././././././15.44	.
12:57801373G>T	rs560739712	ALS6_5609	TSFM	0.001951	././benign/./././././././11.40	.
12:57807722C>G	rs150785031	ALS2_503	TSFM	0.001091	././pathogenic/./1/0.999/1/3.28/T/28.6	.
12:65436663A>G	rs146310140	ALS6_5609	MSRB3	0.001573	././benign/./././././././11.50	.
12:65459278C>T	rs764167816	ALS8_5611	MSRB3	0.001059	././benign/./././././././10.18	.
12:101714235C>T	.	ALS8_5611	CHPT1	0	deleterious/possibly_damaging/pathogenic/./0.675/0.351/1/2.285/T/31	.
12:101717303A>G	rs757223358	ALS8_5611	CHPT1	0.000197	././benign/./././././././14.26	.
12:104308878A>G	rs117196303	ALS3_536; ALS6_5609	TXNRD1	0.00589	././benign/./././././././14.02	.
12:105045410A>C	rs186839012	ALS8_5611	ALDH1L2	0.002269	././benign/./././././././10.69	.
12:109548440A>C	rs1485878921	ALS6_5609	NA	2.63E-05	././benign/./././././././14.22	.
12:110535360CATTCTT>C	rs1487066813	ALS1_497	PPTC7	0.000131	./././././././././12.82	.
12:110581650C>T	rs139936355	ALS5_5608	PPTC7	0.00207	././benign/./././././././13.40	.
12:120515156A>G	rs921605157	ALS7_5610	COQ5	7.9E-05	././benign/./././././././11.60	.
12:120530067CTG>C	rs575206600	ALS2_503	COQ5	0.00376	./././././././././15.12	.
12:122200274G>A	rs201278911	ALS4_539	LRRC43	0.000762	tolerated/benign/benign/./0.035/0.019/0.997/1.19/T/10.84	.
12:122206937T>TA	rs745507521	ALS5_5608	DIABLO	0.000237	./././././././././24.8	.
12:123261282C>A	rs41276694	ALS7_5610	C12orf65	0.001558	././pathogenic/./././././././14.19	.
13:21518872A>C	rs116841139	ALS3_536	MICU2	0.005271	././benign/./././././././10.20	.
13:21564339G>A	rs117665658	ALS1_497	MICU2	0.006193	././benign/./././././././10.28	.
13:21576346A>G	rs142228016	ALS8_5611	MICU2	0.00637	././benign/./././././././13.22	.
13:23743263T>C	rs184338018	ALS2_503	MIPEP	0.002787	././benign/./././././././15.32	.
13:40809725G>T	rs17090557	ALS1_497	SLC25A15	0.0057	././pathogenic/Likely_benign/./././././16.73	Hyperornithinemia-hyperammonemia-homocitrullinuria_syndrome
13:41600776G>C	rs113422215	ALS4_539	VWA8	0.005573	././benign/./././././././10.83	.
13:41615006C>T	rs73464952	ALS1_497	VWA8	0.004568	deleterious/possibly_damaging/benign/./0.996/0.781/1/2.34/T/23.8	.

13:41659961T>C	rs36093708	ALS3_536	VWA8	0.006484	./../benign/./././././././13.50	.
13:41704667C>T	rs12872656	ALS3_536	VWA8	0.003001	./../benign/./././././././10.30	.
13:43064930A>C	rs112965900	ALS4_539:<br>ALS5_5608	DNAJC15	0.005568	./../benign/./././././././11.76	.
13:47954201C>A	rs1245036990	ALS8_5611	SUCLA2	0	deleterious/probably_damaging/pathogenic/./1/1/1/4.885/D/26.2	.
13:48000448G>A	rs540656578	ALS4_539	SUCLA2	0.000585	./../benign/./././././././11.13	.
13:76957044A>G	rs116855637	ALS1_497	ACOD1	0.006669	deleterious/probably_damaging/pathogenic/./././0.993/2.355/T/25.3	.
13:99608942T>G	rs1306842617	ALS4_539	CLYBL	0.00279	./../benign/./././././././11.99	.
13:99644510A>C	rs572421145	ALS8_5611	CLYBL	0.002681	./../benign/./././././././12.48	.
13:99687807A>G	rs145405115	ALS7_5610	CLYBL	0.007766	./../benign/./././././././15.51	.
13:99698937T>C	rs571053083	ALS2_503	CLYBL	0.000296	./../pathogenic/./././././././21.3	.
13:99734808A>C	.	ALS8_5611	CLYBL	0	./../benign/./././././././10.38	.
13:99764847A>AT	rs756835141	ALS1_497	CLYBL	0.03148	./././././././././././10.83	.
13:99799525G>A	rs772327955	ALS1_497	CLYBL	3.94E-05	./../benign/./././././././13.98	.
13:99824682T>C	rs117372177	ALS1_497	CLYBL	0.014365	./../benign/./././././././14.31	.
13:99838100A>G	rs11840555	ALS1_497	CLYBL	0.021581	./../benign/./././././././14.78	.
13:99844622C>T	rs1156647734	ALS4_539	CLYBL	2.63E-05	./../benign/./././././././11.56	.
13:99857487A>C	rs551189169	ALS8_5611	CLYBL	0.00021	./../pathogenic/./././././././11.10	.
13:100119093A>G	rs184566622	ALS3_536	PCCA	0.001941	./../benign/./././././././11.49	.
13:100221531G>C	rs554147983	ALS8_5611	PCCA	0.00094	./../benign/./././././././10.09	.
13:100306635T>C	rs188919545	ALS4_539	PCCA	0.001562	./../pathogenic/./././././././15.31	.
13:100409218C>A	rs189134186	ALS2_503	PCCA	0.001617	./../benign/./././././././11.91	.
13:100522709G>A	rs557247098	ALS2_503	PCCA	0.002071	./../pathogenic/./././././././20.5	.
13:100526466G>T	rs1004400979	ALS4_539	PCCA	0	./../benign/./././././././10.45	.
13:110683132T>C	rs150286306	ALS2_503	CARS2	0.000769	tolerated/benign/benign/Uncertain_significance/0.005/0.02/0.849/-0.84/T/22.1	Combined_oxidative_phosphorylation_deficiency_27|Inborn_genetic_diseases
14:23310153G>A	rs543708304	ALS5_5608	BCL2L2	0.000947	./../benign/./././././././11.51	.
14:24094544G>A	rs561035624	ALS5_5608	PCK2	0.003417	./../benign/./././././././17.16	.
14:24287248C>T	rs74485895	ALS5_5608	DHRS1	0.001163	./../benign/./././././././11.00	.
14:24303966G>T	rs189010949	ALS3_536	DHRS1	0.000874	./../benign/./././././././11.36	.
14:24305018C>T	rs201242778	ALS3_536	NOP9	0.000375	deleterious/possibly_damaging/benign/./0.998/0.819/1/0.895/T/28.3	.
14:31690126A>C	rs185824116	ALS5_5608	NUBPL	0.001332	./../benign/./././././././10.77	.
14:31801847A>G	rs1397191929	ALS1_497	NUBPL	1.31E-05	./../benign/./././././././10.55	.
14:31848903C>CT	.	ALS4_539	NUBPL	2.63E-05	./././././././././././14.24	.
14:31848904A>AG	.	ALS4_539	NUBPL	2.63E-05	./././././././././././14.29	.
14:35203946A>T	rs138842527	ALS6_5609	KIAA0391	0.00842	./../benign/./././././././15.06	.
14:35204024T>G	rs74752973	ALS6_5609	KIAA0391	0.008395	./../benign/./././././././15.30	.

14:35224887C>CT	rs993242741	ALS1_497	DPRXP3	0.000237	./././././././././13.18	.
14:36671971C>T	rs565545610	ALS7_5610	PAX9	0.001486	././benign/./././././././15.93	.
14:36676130T>G	.	ALS4_539	SLC25A21	0	././benign/./././././././10.58	.
14:36677543AC>A	rs201941449	ALS1_497; ALS7_5610	SLC25A21	0.005347	././Warning:/./././././././14.05	.
14:36719763T>G	rs569626298	ALS8_5611	SLC25A21	0.000611	././benign/./././././././14.91	.
14:36734190G>C	rs143539961	ALS4_539	SLC25A21	0.000836	././benign/./././././././14.87	.
14:36742967A>T	rs537989009	ALS4_539	SLC25A21	0.000835	././benign/./././././././10.14	.
14:36805357C>A	rs887879406	ALS8_5611	SLC25A21	3.29E-05	././benign/./././././././14.97	.
14:36810369G>C	rs541899469	ALS1_497	SLC25A21	0.004254	././benign/./././././././14.76	.
14:36848617A>G	rs537925955	ALS6_5609; ALS7_5610	SLC25A21	0.002168	././benign/./././././././12.30	.
14:36880721C>A	rs143857917	ALS3_536	SLC25A21	0.003293	././benign/./././././././15.41	.
14:36918621C>T	rs184596038	ALS1_497	SLC25A21	0.002803	././benign/./././././././16.65	.
14:36934194A>T	rs144214659	ALS3_536	SLC25A21	0.004929	././benign/./././././././10.26	.
14:37069879C>A	rs184512432	ALS7_5610	SLC25A21	0.00301	././benign/./././././././11.63	.
14:37080267T>C	rs771329095	ALS2_503	SLC25A21	5.26E-05	././benign/./././././././12.44	.
14:50249392C>T	rs531609885	ALS7_5610	L2HGDH	0.001045	././benign/./././././././10.19	.
14:67620024A>G	rs17185189	ALS7_5610	ARG2	0.0043	tolerated/benign/benign/./0/0.001/1/0.525/T/10.34	.
14:67620335C>T	rs559246839	ALS6_5609	ARG2	0.001376	././benign/./././././././15.40	.
14:67651918C>T	rs181149950	ALS4_539	ARG2	0.003233	././benign/./././././././11.18	.
14:69228807C>T	rs368100825	ALS8_5611	EXD2	9.86E-05	././pathogenic/./././././././16.28	.
14:70344337A>G	rs142286905	ALS2_503	COX16	0.003317	././benign/./././././././11.22	.
14:70388458GC>G	.	ALS1_497	SYNJ2BP	0	./././././././././15.71	.
14:73950229C>T	.	ALS6_5609	COQ6	0	././benign/./././././././13.94	.
14:73961778C>T	rs201434148	ALS7_5610	COQ6	0.000158	deleterious/benign/pathogenic/./0.244/0.133/1/2.205/T/24.6	.
14:74066599T>C	rs769234420	ALS4_539	ALDH6A1	0.000151	././benign/./././././././12.62	.
14:74882509A>G	rs185461540	ALS1_497	DLST	0.000914	././benign/./././././././14.22	.
14:77865720T>C	.	ALS1_497	ADCK1	0	././benign/./././././././10.15	.
14:77868965G>A	rs775473644	ALS4_539	ADCK1	8.54E-05	././benign/./././././././16.00	.
14:93344034T>G	rs1267890632	ALS8_5611	COX8C	0.001557	././benign/./././././././16.75	.
14:95532442A>AGGT	rs532109833	ALS5_5608	GLRX5	0.002733	./././././././././11.38	.
14:100320435T>C	rs118060880	ALS1_497	SLC25A47	0.005227	././benign/./././././././13.95	.
15:43700427A>G	rs397834749	ALS3_536	CKMT1A	0.004367	././benign/./././././././16.35	.
15:43700428G>T	rs535882235	ALS3_536	CKMT1A	0.004366	././benign/./././././././14.10	.
15:45361410T>G	rs750817737	ALS1_497	GATM	0.000946	././benign/Uncertain_significance/./././././.13.78	Arginine:glycine_amidinotransferase_deficiency
15:45368101T>C	rs759012962	ALS4_539	GATM	0	tolerated/probably_damaging/pathogenic/Uncertain_significance/1/0.999/1/1.77/T/24.8	Arginine:glycine_amidinotransferase_deficiency

15:45376722G>A	.	ALS6_5609	GATM	0	tolerated/benign/pathogenic/./0/0.001/0.862/-0.235/T/22.3	.
15:65027202CT>C	rs544080836	ALS2_503; ALS3_536	MTFMT	0.001906	./././././././././11.12	.
15:65175610T>C	rs183566733	ALS2_503	CLPX	0.001525	././benign/./././././././11.74	.
15:76253599C>T	rs191775189	ALS5_5608	ETFA	0.000211	././benign/./././././././10.05	.
15:76280593A>G	rs187611885	ALS1_497	ETFA	0.000605	././benign/./././././././13.82	.
15:76295259C>A	rs1166715405	ALS6_5609	ETFA	6.57E-06	././benign/./././././././16.51	.
15:76305693C>T	rs147506144	ALS6_5609	ETFA	0.001749	././benign/./././././././12.75	.
15:78168982C>T	rs116374996	ALS2_503	IDH3A	0.002675	deleterious/benign/pathogenic/Likely_benign/0.664/0.258/1/2.73/T/24.9	not_provided
15:88476750G>A	rs117548718	ALS4_539	MRPS11	0.006337	././benign/./././././././14.94	.
15:90266255G>T	rs201430256	ALS6_5609	NGRN	0.001525	././benign/./././././././13.93	.
16:662270C>T	rs148985409	ALS2_503	WDR90	0.003759	tolerated/benign/benign/./1/0.998/1/1.915/T/13.49	.
16:1420582C>G	rs45490596	ALS3_536	C16orf91	0.005215	tolerated/benign/benign/./0.043/0.024/1/0.935/T/14.27	.
16:1772442C>G	rs139953295	ALS5_5608	MRPS34	0.003436	tolerated/probably_damaging/pathogenic/./0.999/0.948/0.998/2.7/T/25.5	.
16:1775066C>T	rs148313225	ALS6_5609	MRPS34	0.000604	././benign/./0.606/0.372/1/1.905/T/23.2	.
16:1778029G>A	rs35816944	ALS6_5609	MRPS34	0.003687	././pathogenic/./0.969/0.558/0.999/0.72/T/25.0	.
16:1984584C>A	rs550296574	ALS4_539	GFER	0.002142	././benign/./././././././10.08	.
16:3721457T>G	.	ALS4_539	TRAP1	0	././benign/./././././././12.75	.
16:4441400A>G	.	ALS6_5609	DNAJA3	0	tolerated/benign/pathogenic/./0.285/0.091/1/0.37/T/23.5	.
16:8682582C>G	rs141354253	ALS4_539	ABAT	0.000406	././benign/./././././././11.69	.
16:20465616G>A	rs141326932	ALS3_536	ACSM2A	0.000276	tolerated/benign/pathogenic/./0.052/0.091/1/0.945/T/13.65	.
16:20794779C>A	rs142404681	ALS3_536	ACSM3	0.005041	././benign/./././././././10.17	.
16:20915595C>T	rs149175521	ALS5_5608	LYRM1	0.000388	deleterious/probably_damaging/pathogenic/./1/1/1/3.44/D/29.1	.
16:23556714T>G	rs2234422	ALS5_5608	EARS2	0.005388	././benign/./././././././10.17	.
16:28834571G>A	rs7499664	ALS5_5608	ATXN2L	0.001702	deleterious/possibly_damaging/pathogenic/./0.997/0.839/1/1.905/T/24.6	.
16:28845000T>C	rs201756974	ALS4_539	TUFM	6.57E-05	tolerated/benign/pathogenic/Uncertain_significance/0.018/0.009/0.958/-0.37/T/22.5	Combined_oxidative_phosphorylation_deficiency_4
16:68015505A>G	rs150890440	ALS6_5609	DUS2	0.000877	././benign/./././././././12.54	.
16:68085961G>T	rs921754118	ALS5_5608	NFATC3	4.6E-05	././benign/./././././././15.31	.
16:69464598T>C	rs534941397	ALS8_5611	CYB5B	0.000447	././benign/./././././././13.88	.
16:69464728G>A	rs975433596	ALS5_5608	CYB5B	2.63E-05	././benign/./././././././11.16	.
16:70156611G>A	rs117263218	ALS3_536	PDPR	0.002775	deleterious/probably_damaging/pathogenic/./0.999/0.936/1/2.725/D/32	.
16:70156640A>G	rs201033817	ALS7_5610	PDPR	0.001445	deleterious/probably_damaging/pathogenic/./1/0.998/1/4.06/D/26.7	.

16:80981911C>T	.	ALS5_5608	CMC2	0	./../benign/./././././././15.00	.
16:85800009C>A	rs898250068	ALS8_5611	COX4I1	0.000657	./../benign/./././././././17.75	.
17:4949611AGGGCGGGAC>A	rs528614399	ALS3_536	PFN1	0.000355	./././././././././10.86	.
17:7207348GAGCC>G	rs543025747	ALS8_5611	SLC25A35	0.004747	./././././././././13.76	.
17:7211821C>CGG	rs1318925894	ALS6_5609	SLC25A35	0.002996	./././././././././12.36	.
17:8292555C>T	rs146737646	ALS6_5609	SLC25A35	0.000434	tolerated/benign/benign/./0.629/0.326/0.961/0.73/T/23.5	.
17:8299016A>G	rs146571796	ALS5_5608	SLC25A35	0.005492	./../benign/./././././././11.57	.
17:14083986C>A	rs116935344	ALS4_539	COX10	0.002598	./../benign/./././././././12.09	.
17:14147952T>C	rs73979168	ALS2_503	COX10	0.008693	./../benign/./././././././10.68	.
17:18255257C>T	rs138000313	ALS8_5611	FLII	0.000795	tolerated/possibly_damaging/pathogenic/./1/0.914/1/1.75/T/22.9	.
17:18315487G>A	rs139090902	ALS2_503	TOP3A	0.003593	./../benign/./././././././14.98	.
17:19665048G>A	.	ALS1_497	ALDH3A2	0	./../pathogenic/Likely_pathogenic/././1/././34	**Sjgren**-Larsson_syndrome
17:21132892AT>A	rs531121612	ALS3_536;ALS4_539	DHRS7B	0.001089	./././././././././10.29	.
17:32208185A>G	rs139664057	ALS5_5608	RHOT1	0.000532	tolerated/benign/benign/./0.002/0.002/1/-0.565/T/16.99	.
17:37164226C>T	rs79949332	ALS1_497	ACACA	0.007489	./../pathogenic/./././././././18.50	.
17:37176896T>A	rs149221856	ALS1_497	ACACA	0.002932	./../benign/./././././././15.72	.
17:37202553T>TA	rs1208918443	ALS2_503	ACACA	0.000946	./././././././././15.12	.
17:37233460G>A	rs12937687	ALS4_539	ACACA	0.003929	./../pathogenic/./././././././18.04	.
17:38731375T>A	.	ALS7_5610	CISD3	6.58E-06	deleterious/possibly_damaging/pathogenic/./0.921/0.691/0.831/2.27/T/26.9	.
17:42568087C>T	rs541382133	ALS6_5609	COASY	0.00021	./../benign/./././././././12.07	.
17:43199739C>T	rs1384764445	ALS2_503	NBR1	0	./../benign/./././././././10.14	.
17:43216403A>G	rs201122046	ALS8_5611	NBR1	0.000315	./../benign/./././././././15.50	.
17:50115869C>T	rs80248520	ALS8_5611	PDK2	0.003403	./../pathogenic/./1/0.982/0.958/1.995/T/28.1	.
17:50362160A>T	rs546433529	ALS1_497	XYLT2	3.94E-05	./../benign/./././././././15.80	.
17:50540181G>A	rs781087683	ALS3_536	SPATA20	0.000585	./../benign/./././././././15.02	.
17:50565009A>G	.	ALS5_5608	CACNA1G	0	./../benign/./././././././17.67	.
17:57075202T>C	rs72843409	ALS7_5610	AKAP1	0.00262	./../benign/./././././././10.55	.
17:57840922T>G	rs938915754	ALS2_503	MRPS23	0	./../pathogenic/./././1/././22.5	.
17:58518972C>G	rs1008716867	ALS2_503	SEPT4	1.97E-05	./../benign/./././././././11.74	.
17:58531444T>C	rs150891701	ALS7_5610	SEPT4	0.005749	./../benign/./././././././10.01	.
17:59694241C>T	rs145708582	ALS6_5609	PTRH2	0.004233	./../benign/./././././././11.73	.
17:68984907G>T	rs148927389	ALS7_5610	ABCA9	0.004694	deleterious/probably_damaging/pathogenic/./0.999/0.995/1/2.96/D/23.4	.
17:69027383C>T	rs79212004	ALS3_536	ABCA9	0.00161	tolerated/possibly_damaging/pathogenic/./0.923/0.559/1/3.48/T/21.3	.
17:75047230C>A	rs1268258474	ALS6_5609	ATP5PD	1.35E-05	./../benign/./././././././13.42	.

17:75047949C>A	rs547077735	ALS5_5608	ATP5PD	0.002228	./../benign/./././././../13.09	.
17:75266752T>A	rs138489085	ALS4_539	MRPS7	0.000164	./../pathogenic/./0.993/0.967/1/2.085/T/23.6	.
17:75292549CT>C	rs757201892	ALS2_503	SLC25A19	0.002038	./././././././././10.64	.
17:75914150C>T	rs773400065	ALS4_539	FBF1	1.97E-05	deleterious/benign/benign/./0.887/0.179/0.996/2.6/T/22.5	.
17:78378682C>T	rs200541156	ALS3_536	PGS1	0.005688	tolerated/probably_damaging/pathogenic/./0.98/0.935/0.605/0.69/T/22.9	.
17:78403579G>A	rs201765653	ALS1_497	PGS1	5.92E-05	tolerated/benign/benign/./0.008/0.001/1/-0.26/T/17.22	.
17:81924943T>C	rs757356237	ALS8_5611	MAFG	0.000105	./../benign/./././././../15.90	.
18:2541907T>C	.	ALS3_536	METTL4	0	./../benign/./././././../14.95	.
18:2574875T>G	rs186283183	ALS8_5611	METTL4	0.003127	./../benign/./././././../22.1	.
18:9119262T>C	rs41274296	ALS6_5609	NDUFV2	0.005907	./../benign/Likely_benign/./././././10.75	not_provided
18:9138684A>G	rs75099041	ALS6_5609	NDUFV2	0.005918	./../pathogenic/./././././../19.00	.
18:9141620G>T	rs184360741	ALS1_497	NDUFV2	0.002853	./../benign/./././././../12.42	.
18:9142305T>C	rs117942008	ALS6_5609	NDUFV2	0.002608	./../benign/./././././../11.59	.
18:12322543TG>T	rs535384834	ALS3_536	TUBB6	0.000671	./././././././././13.00	.
18:12377153G>A	rs571340907	ALS4_539	AFG3L2	0.005938	./../benign/Benign/Likely_benign/./././././10.02	Spinocerebellar_ataxia_type_28|not_provided
18:12466248T>C	rs1020573012	ALS4_539	SPIRE1	3.94E-05	./../benign/./././././../13.38	.
18:12471091C>T	rs529668495	ALS7_5610	SPIRE1	0.00627	./../benign/./././././../11.08	.
18:12479755G>C	rs373281197	ALS1_497	SPIRE1	1.31E-05	deleterious/benign/benign/./0.333/0.108/0.538/2.975/T/20.6	.
18:13681911A>C	rs151157304	ALS4_539	FAM210A	3.29E-05	deleterious/probably_damaging/pathogenic/./1/0.999/0.993/2.505/T/24.6	.
18:13726581G>T	rs554143670	ALS3_536	FAM210A	0.00023	./../pathogenic/./././././../19.76	.
18:13731836A>G	rs143290824	ALS3_536	RNMT	0.00023	tolerated/benign/benign/./0.157/0.051/0.998/0.69/T/15.78	.
18:24234511T>C	rs61125050	ALS1_497	OSBPL1A	0.008674	./../benign/./././././../11.88	.
18:24264254G>GA	rs371588904	ALS1_497	OSBPL1A	0.010224	./././././././././15.54	.
18:24395155C>T	rs146527591	ALS8_5611	OSBPL1A	0.004764	./../benign/./././././../15.37	.
18:46084534C>T	.	ALS4_539	ATP5F1A	0	tolerated/benign/pathogenic/./0/0.002/1/1.235/T/22.4	.
18:50881524G>T	rs1337541880	ALS3_536	ME2	0	./../benign/./././././../13.50	.
18:63152365T>G	rs144031174	ALS6_5609	BCL2	0.004447	./../benign/./././././../13.68	.
18:63155666A>T	rs977024450	ALS7_5610	BCL2	7.89E-05	./../benign/./././././../11.15	.
18:63253835GA>G	rs1346311538	ALS2_503	BCL2	0.001813	./././././././././15.29	.
18:63320237GAAGA>G	rs1037532057	ALS8_5611	BCL2	0.000149	./././././././././14.96	.
18:75204513C>T	rs764292109	ALS3_536	ZADH2	0.00111	./../benign/./././././../10.23	.
18:80055904C>G	rs540444302	ALS1_497	RBFADN	0.000204	./../benign/./././././../13.14	.
19:635424C>T	rs541034247	ALS1_497	POLRMT	0.000171	./../benign/./././././../14.19	.
19:1113681C>T	rs765765176	ALS2_503	SBNO2	1.31E-05	tolerated/benign/pathogenic/./0.1/0.073/0.611/1.95/T/19.60	.

19:1248497G>C	rs1007783413	ALS8_5611	ATP5F1D	0.000102	./.benign/./././././.16.70	.
19:1248501G>C	rs1019127196	ALS8_5611	ATP5F1D	8.46E-05	./.benign/./././././.12.39	.
19:1248509G>C	rs1458908167	ALS8_5611	ATP5F1D	0.000274	./.benign/./././././.16.85	.
19:2434019C>T	rs555606326	ALS6_5609	LMNB2	1.31E-05	deleterious/probably_damaging/benign/./0.998/0.847/0.991/1.335/T/24.9	.
19:3759732G>A	rs138562152	ALS5_5608	MRPL54	0.001072	./.benign/./0.27/0.026/0.968/1.4/T/15.79	.
19:5692106C>T	rs367780804	ALS2_503	LONP1	0.000143	deleterious/probably_damaging/pathogenic/./1/0.998/1/3.72/D/26.9	.
19:5720218C>T	rs112324623	ALS7_5610	LONP1	0.002325	./.benign/Likely_benign/./././././12.72	CODAS_syndrome
19:6432083G>A	rs200270386	ALS7_5610	SLC25A41	0.000243	deleterious/probably_damaging/pathogenic/./1/0.957/1/2.9/D/25.5	.
19:6464292G>A	rs569671916	ALS7_5610	SLC25A23	0.000801	./.benign/./././././.14.94	.
19:8307860C>T	rs180797395	ALS6_5609	NDUFA7	0.003096	./.benign/./././././.12.38	.
19:10259727T>C	rs142765226	ALS6_5609	MRPL4	0.001224	tolerated/benign/pathogenic/./0.152/0.059/1/0/T/20.8	.
19:11529580C>T	rs150929921	ALS4_539	ECSIT	0.00079	./.benign/./././././.11.93	.
19:12788836C>T	rs1009172567	ALS8_5611	JUNB	6.57E-06	./.benign/./././././.16.51	.
19:12790369T>C	rs562884462	ALS7_5610	JUNB	0.001769	./.benign/./././././.18.83	.
19:12954497T>C	rs149233851	ALS7_5610	GADD45GIP1	0.00209	deleterious/possibly_damaging/pathogenic/./0.907/0.524/0.958/2.28/T/26.0	.
19:13096950G>GA	rs112879585	ALS4_539	NFIX	0.002929	./././././././././14.60	.
19:13109979A>C	rs771096015	ALS1_497	TRMT1	1.31E-05	deleterious/probably_damaging/pathogenic/./1/1/1/4.14/D/32	.
19:14118204G>C	rs1032868709	ALS7_5610	PRKACA	0.003808	./.benign/./././././.19.30	.
19:14118233G>A	rs1420557074	ALS5_5608	PRKACA	2.03E-05	./.benign/./././././.18.65	.
19:14560229G>T	rs1978626	ALS1_497	TECR	0.001108	./.benign/./././././.12.02	.
19:18545339CT>C	rs576891720	ALS3_536	FKBP8	0.000975	./././././././././12.12	.
19:19535632C>G	rs146733860	ALS1_497	YJEFN3	0.005283	tolerated/probably_damaging/benign/./1/0.992/1/1.83/T/23.6	.
19:32895750T>TA	rs113777086	ALS1_497	CEP89	0.003457	./././././././././10.54	.
19:35807078AG>A	rs755582106	ALS6_5609	PRODH2	0.000388	./././././././././32	.
19:38907561C>T	rs187346322	ALS4_539	NFKBIB	0.000525	tolerated/benign/benign/./0.009/0.005/0.994/2.42/T/21.1	.
19:41426222G>A	rs137913069	ALS2_503	BCKDHA	0.002182	./.pathogenic/./1/0.989/0.968/1.5/T/24.8	.
19:43525635C>T	rs750638520	ALS1_497	ETHE1	0.000177	./.benign/./././././.14.12	.
19:44883203A>G	rs76725281	ALS3_536; ALS7_5610	NECTIN2	0.015928	./.benign/./././././.11.67	.
19:45591958T>G	rs536994157	ALS1_497	OPA3	0.004576	./.pathogenic/./././././.15.87	.
19:45767559G>A	rs138291642	ALS6_5609	DMPK	0.003042	./.benign/./././././.19.16	.
19:47230968G>A	rs901494477	ALS2_503	BBC3	8.56E-05	./.benign/./././././.18.57	.
19:47232589A>G	rs45477493	ALS2_503	BBC3	0.002936	deleterious/benign/benign/./0.013/0.004/1/0/T/12.56	.
19:48332539T>G	rs1316711001	ALS3_536	TMEM143	6.57E-06	./.benign/./././././.12.46	.
19:48629592T>C	rs754083757	ALS8_5611	SPHK2	0	tolerated/benign/benign/./0.989/0.648/1/1.74/T/22.6	.

19:48635643G>C	rs555937572	ALS6_5609	SPHK2	0.00173	./.benign/./0.369/0.091/0.915/1.5/T/20.8	.
19:51368599G>A	rs138507210	ALS7_5610	ETFB	0.01083	./.benign/./././././12.78	.
19:55062802G>GACCTGGCCGGCC	rs1359976349	ALS1_497	RDH13	0.001064	./././././././././13.74	.
19:55361093C>T	rs145275620	ALS1_497	FAM71E2	0.003461	tolerated/benign/benign/./0.013/0.005/1/0.345/T/11.72	.
19:55464389T>G	rs1055912659	ALS3_536	ISOC2	1.32E-05	./.benign/./././././22.0	.
20:2655111ATAGAAAGAGGAGG>A	.	ALS5_5608	IDH3B	0	./././././././././18.42	.
20:3168342G>A	rs180708780	ALS4_539	LZTS3	0.001547	./.benign/./././././15.89	.
20:3880500G>A	rs186126558	ALS7_5610	MAVS	0.006105	./.benign/./././././11.98	.
20:3891972G>T	rs185319894	ALS4_539	PANK2	0.001321	./.benign/./././././11.43	.
20:6006764A>G	.	ALS2_503	CRLS1	0	./.benign/./././././10.13	.
20:6019560T>C	rs149361586	ALS6_5609	CRLS1	0.005823	./.benign/./././././12.45	.
20:31669244G>A	rs138191924	ALS7_5610	BCL2L1	0.002846	./.benign/./././././13.94	.
20:31694415G>A	rs73117919	ALS5_5608	BCL2L1	0.001026	./.benign/./././././19.08	.
20:31723587C>T	rs571505406	ALS5_5608	BCL2L1	0.005829	./.benign/./././././17.40	.
20:35662961CAAAGT>C	rs548165033	ALS7_5610	CPNE1	0.00161	./././././././././12.27	.
20:44966567C>G	rs190920315	ALS4_539	STK4	0.002733	./.pathogenic/./././././21.1	.
20:54169957T>A	rs149998531	ALS3_536	CYP24A1	0.004154	./.benign/./././././13.49	.
21:31671122AAAAT>A	rs1057273288	ALS2_503	SOD1	0.001078	./././././././././12.93	.
21:33909192T>C	rs140827929	ALS3_536	ATP5PO	0.003888	tolerated/benign/pathogenic/./0.024/0.08/1/2.145/T/23.0	.
21:34074476G>C	rs115729130	ALS3_536	MRPS6	0.006472	./.benign/./././././17.75	.
21:34074488C>A	rs553429190	ALS3_536	MRPS6	0.000269	./.benign/./././././17.40	.
21:34097704A>T	rs115860747	ALS3_536	MRPS6	0.004703	./.benign/./././././13.07	.
21:34105041CTT>C	rs146361117	ALS3_536	MRPS6	0.005346	./././././././././10.19	.
21:34113185C>T	rs114699237	ALS3_536	MRPS6	0.005293	./.benign/./././././12.51	.
21:46287125T>TAAAAAAA	rs58271568	ALS3_536	YBEY	0.00087	./././././././././16.27	.
22:19930742G>A	rs189705806	ALS5_5608	TXNRD2	0.000801	./.benign/./././././11.64	.
22:19949559G>C	rs188233565	ALS4_539	COMT	0.000964	./.benign/./././././13.00	.
22:19971942G>A	rs34687532	ALS5_5608	COMT	0.00067	./.benign/./0.007/0.004/0.873/1.5/T/17.97	.
22:19972989A>G	rs115344498	ALS2_503	COMT	0.001303	./.pathogenic/Likely_benign/0.005/0.007/0.999/2.275/T/23.6	not_provided
22:23768447A>G	rs555114361	ALS8_5611	CHCHD10	0.005464	./.benign/./././././13.00	.
22:37024382G>T	rs201058049	ALS6_5609	MPST	0.002615	deleterious/probably_damaging/pathogenic/./1/0.997/1/4.92/D/25.1	.
22:40896050T>A	rs148928334	ALS4_539	XPNPEP3	0.000276	./.benign/./././././12.07	.

22:41515801G>C	rs141878785	ALS7_5610	ACO2	0.000933	deleterious/possibly_damaging/pathogenic/Uncertain_significance/0.861/0.62/1/2.57/D/24.7	Retinal_dystrophy∣Infantile_cerebellar-retinal_degeneration∣Optic_atrophy_9∣not_provided
22:42076885G>C	rs537373965	ALS5_5608	SMDT1	0.001578	./benign/./././././12.52	.
22:42086248G>A	rs113437301	ALS5_5608	NDUFA6	0.003719	tolerated/benign/pathogenic/Likely_benign/0.008/0.004/0.999/1.3/T/22.5	not_specified
22:42615296C>T	rs148484665	ALS8_5611	CYB5R3	0.006204	././pathogenic/./././././18.56	.
22:42615315C>T	rs180887630	ALS1_497	CYB5R3	0.002907	././pathogenic/./././././15.91	.
22:42640000ATCTC>A	rs761239976	ALS5_5608	CYB5R3	1.31E-05	./././././././././22.6	.
22:50573628C>T	rs144936442	ALS5_5608	CPT1B	3.94E-05	deleterious/possibly_damaging/benign/./0.999/0.92/0.999/2.77/D/22.8	.
22:50582829C>A	rs41282357	ALS6_5609	CPT1B	0.006557	././benign/Benign/././././16.62	Megaconial_type_congenital_muscular_dystrophy∣not_provided
X:1388460C>T	rs1365596781	ALS2_503	SLC25A6	0.000577	./././././././././10.61	.
X:24459860C>T	rs753480552	ALS7_5610	NA	0.003647	./././././././././11.24	.
X:24489234A>G	.	ALS6_5609	PDK3		./././././././././15.99	.
X:55028316G>A	rs143742765	ALS3_536	ALAS2	0.003912	./././././././././11.18	.
X:91443567A>G	.	ALS6_5609	PABPC5		./././././././././10.07	.
X:119470504C>T	rs747700813	ALS2_503	SLC25A5	0.001639	tolerated/benign/././0.583/0.055/1/2.935/T/23.6	.
X:155055309A>G	rs933583357	ALS1_497	FUNDC2	0.000134	./././././././././11.17	.
X:155066486AAG>A	rs1257819775	ALS7_5610	CMC4	6.22E-05	./././././././././15.20	.

Table S8. Fifty-two prioritized mtDNA variants sites in the WGS cohort

Locus	Nucleotide/ AA change	Variant ID	Variant nature	Cases	AF_Hom	AF_Het	Pathogenicity (Hmtdb)	ClinVarSignificance/MutPred_Prediction/Panther_Prediction/PhDSNP_Prediction/SNPsGO_Prediction/Polyphen2HumDiv_Prediction/Polyphen2HumVar_Prediction	pon_tRNA/MitoTIP_prediction	Mitomap Disease
CR -HV2	65TG>T/.	.	Het	ALS3_536		0.000659548		./././././././	./.	
CR - HV2	143G>A/.	rs375589100	Het	ALS3_536		0.000141781	pending classification,	./././././././	./.	-
CR - HV2	183A>G/.	rs113913230	Hom	ALS1_497	0.00846135		pending classification,	./././././././	./.	-
CR - HV2	204T>C/.	rs3135032	Het	ALS7_5610		0.00498455	pending classification,	./././././././	./.	-
CR - HV2 - TFX mtTF1 bind site	247G>T/.	rs41334645	Het	ALS6_5609		0	-	./././././././	./.	-
CR - HV3 - TFL mtTF1 bind site	539T>A/.	.	Het	ALS6_5609		0	-	./././././././	./.	-
12 S	1474G>A/.	.	Het	ALS1_497		0	pending classification,	./././././././	./.	-
12 S	1485G>A/.	.	Het	ALS2_503		1.77208E-05	-	./././././././	./.	-
12 S	1589C>T/.	.	Het	ALS3_536		0	-	./././././././	./.	-
MT-TV	1664G>A/.	rs200807305	Het	ALS1_497		0.000106349	polymorphic, , tRNA	Benign/./././././.	Neutral/likely_benign	-
16 S	1700T>C/.	rs2854126	Hom	ALS7_5610	0.00781471		pending classification,	./././././././	./.	-

16 S	1770G>A/.	.	Het	ALS1_497		0.000017723	-	./././././././	./.	-
16 S	2571G>A/.	rs28683810	Het	ALS2_503		1.77261E-05	pending classification,	./././././././	./.	-
MT-ND1	3380G>A/R25Q	rs1603218926	Het	ALS7_5610		0	pathogenic	Pathogenic/high_pathogenicity/./neutral/disease/probably_damaging/probably_damaging/	./.	MELAS
MT-ND1	3454G>A/A50T	.	Het	ALS2_503		1.77214E-05	pathogenic	./high_pathogenicity/neutral/disease/neutral/probably_damaging/probably_damaging/	./.	-
MT-ND1	3550G>A/A82T	.	Het	ALS1_497		0	likely_benign	./low_pathogenicity/neutral/neutral/neutral/benign/benign/	./.	-
MT-ND1	3796A>G/T164A	rs28357970	Hom	ALS1_497	0.0082589		benign	Benign/low_pathogenicity/neutral/neutral/neutral/benign/benign/	./.	Adult-Onset Dystonia
MT-ND1	3952G>A/A216T	rs1603219215	Het	ALS1_497		0	pathogenic	./low_pathogenicity/neutral/disease/neutral/possibly_damaging/possibly_damaging/	./.	-
MT-TI	4310A>G/.	rs1556422841	Het	ALS7_5610		3.54465E-05	polymorphic, , tRNA	Benign/./././././././	Neutral/likely_benign	-
MT-ND2	4996G>A/R176H	rs1603219712	Het	ALS7_5610		.	pathogenic	./high_pathogenicity/disease/disease/disease/benign/benign/	./.	-
MT-TA	5623G>A/.	.	Het	ALS1_497		.	polymorphic, , tRNA	./././././././	Likely_neutral/likely_benign	-
MT-CO1	6840G>A/A313T	.	Het	ALS1_497		1.77242E-05	pathogenic	./high_pathogenicity/disease/disease/disease/probably_damaging/probably_damaging/	./.	-
MT-CO1	6891A>G/S330G	rs879091068	Hom	ALS5_5608	0.000513947		likely_benign	Benign/low_pathogenicity/neutral/neutral/neutral/benign/benign/	./.	-

MT-CO1	7341C>A/R480X	.	Het	ALS2_503	.	.	pending classification,	./././././././	./.	-
MT-CO2	7793G>A/A70T	.	Het	ALS1_497; ALS2_503	0	0	pathogenic	./low_pathogenicity/./disease/neutral/probably_damaging/probably_damaging/	./.	-
MT-ATP6-MT-ATP8	8557G>A/A11T	rs386829040	Hom	ALS1_497	0.00843732		benign	Benign/low_pathogenicity/neutral/neutral/neutral/benign/benign/	./.	-
MT-ATP6	8584G>A/A20T	rs3135028	Het	ALS1_497		8.86509E-05	benign	Benign/low_pathogenicity/neutral/neutral/neutral/benign/benign/	./.	-
MT-ATP6	9145G>A/A207T	rs1556423622	Het	ALS2_503		5.31736E-05	likely_pathogenic	Benign/low_pathogenicity/./disease/disease/probably_damaging/probably_damaging/	./.	-
MT-ND4L	10677G>A/E70K	rs1603222944	Het	ALS7_5610		0	pathogenic	Uncertain_significance/high_pathogenicity/disease/disease/disease/benign/benign/	./.	-
MT-ND4	11004G>T/R82L	.	Het	ALS7_5610		.	likely_benign	./low_pathogenicity/./neutral/neutral/benign/benign/	./.	-
MT-ND4	11031GA>G/.	rs1556423884	Het	ALS7_5610		0		Uncertain_significance/./././././. /	./.	-
MT-ND4	11456G>A/A233T	.	Het	ALS1_497		0	pathogenic	./low_pathogenicity/neutral/disease/neutral/probably_damaging/probably_damaging/	./.	-
MT-ND5	12818G>A/R161Q	.	Het	ALS7_5610		0	pathogenic	./high_pathogenicity/disease/neutral/disease/probably_damaging/probably_damaging/	./.	-
MT-ND5	12820G>A/A162T	rs200567053	Hom	ALS3_536	0.000177214		likely_benign	Benign/low_pathogenicity/disease/neutral/neutral/benign/benign/	./.	-
MT-ND5	12889G>A/A185T	rs1556424185	Het	ALS1_497; ALS2_503		0.00010637	likely_benign	Uncertain_significance/low_pathogenicity/disease/neutral/neutral/benign/benign/	./.	-
MT-ND5	13528A>G/T398A	rs55882959	Hom	ALS6_5609	0.00209142		likely_pathogenic	Conflicting_interpretations_of_pathogenicity/low_pathogenicity/./neutral/neutral/probably_damaging/probably_damaging/	./.	LHON-like, LHON, MELAS

MT-ND5	13565C>T/S410F	rs56039545	Hom	ALS6_5609	0.00166569		likely_pathogenic	Benign/low_pathogenicity/neutral/neutral/neutral/probably_damaging/probably_damaging/	./.	-
MT-ND5	13810G>A/A492T	rs1603224361	Het	ALS1_497; ALS2_503		1.77223E-05	benign	Benign/low_pathogenicity/disease/neutral/neutral/benign/benign/	./.	-
MT-ND5	14047A>G/I571V	rs1603224486	Hom	ALS1_497; ALS2_504	0.000230366		likely_benign	Likely_benign/low_pathogenicity/neutral/neutral/neutral/benign/benign/	./.	-
MT-ND6	14384G>A/A97V	rs1556424435	Het	ALS1_497; ALS2_503		8.86211E-05	benign	Benign/low_pathogenicity/neutral/neutral/neutral/benign/benign/	./.	-
MT-ND6	14459G>A/A72V	rs199476105	Het	ALS7_5610		1.77204E-05	pathogenic	Pathogenic/low_pathogenicity/disease/disease/disease/probably_damaging/probably_damaging/	./.	LDYT / Leigh Disease / dystonia / carotid atherosclerosis risk
MT-ND6	14577T>G/I33L	rs386829219	Hom	ALS2_503	0.000212642		benign	Likely_benign/low_pathogenicity/neutral/neutral/disease/benign/benign/	./.	-
MT-CYB	14750A>G/T2A	rs1603224853	Hom	ALS6_5609	0.0009393		benign	Benign/low_pathogenicity/neutral/neutral/neutral/benign/benign/	./.	-
MT-CYB	14861G>A/A39T	rs2853505	Het	ALS1_497		0.000141834	benign	Benign/low_pathogenicity/neutral/neutral/neutral/benign/benign/	./.	-
MT-CYB	14921G>A/A59T	rs1603224964	Het	ALS2_503		0	pathogenic	Uncertain_significance/low_pathogenicity/neutral/neutral/neutral/probably_damaging/probably_damaging/	./.	-

MT-CYB	15005G>A/A87T	.	Het	ALS2_503		3.54503E-05	pathogenic	./low_pathogenicity/disease/disease/disease/probably_damaging/probably_damaging/	./.	-
MT-CYB	15200G>A/A152T	rs1603225142	Het	ALS1_497; ALS2_503		5.31934E-05	pathogenic	Uncertain_significance/low_pathogenicity/disease/disease/disease/probably_damaging/probably_damaging/	./.	-
MT-CYB	15591G>A/R282Q	.	Het	ALS2_503		.	pathogenic	./high_pathogenicity/disease/disease/disease/probably_damaging/probably_damaging/	./.	-
MT-CYB	15831T>C/I362T	rs1603225529	Het	ALS2_503		3.54717E-05	pathogenic	Uncertain_significance/low_pathogenicity/./neutral/neutral/probably_damaging/probably_damaging/	./.	-
CR - HV1 - TAS2	16084G>A/.	rs1603225661	Het	ALS2_503		0	-	./././././././	./.	-
CR - HV1	16265A>G/.	rs386829295	Hom	ALS3_536	0.00402461		-	./././././././	./.	-
CR	16496G>A/.	.	Het	ALS2_503		1.80584E-05	-	./././././././	./.	-

Table S9. Clusters of highly interconnected genes as identified by MCODE in the network generated with genes containing variants in the WES cohort. To analyse the physical relationships among genes containing variants, the Cytoscape plug-in Molecular Complex Detection (MCODE, version 2.0.0; http://apps.cytoscape.org/apps/mcode) was used to identify the most important sub-modules of the network map. The criteria of MCODE analysis were degree cut-off = 2, MCODE score > 5, Max depth = 100, node score cut-off = 0.2, and k-score = 2. The Biological Networks Gene Ontology tool (BiNGO, version 3.0.3; http://apps.cytoscape.org/apps/bingo) was used to analyze and visualize the biological processes of identified sub-modules with an FDR corrected P-value<0.001.

Cluster	Score (Density*#Nodes)	Nodes	Edges	Node IDs	GO Biological Processes P<0.001*
1	9	9	34	MRPL43, MRPL40, TSFM, GADD45GIP1, TUFM, MRPL37, GFM1, MRRF, MRPL3	translation, cellular macromolecule biosynthetic process, macromolecule biosynthetic process, gene expression, cellular biosynthetic process, biosynthetic process, translational elongation, cellular protein metabolic process, protein metabolic process, mitochondrial translational elongation,
2	7	8	25	BCS1L, MT-CO2, NDUFB6, MT-CO1, CYC1, NDUFV1, PMPCA, UQCR10	electron transport chain, ATP synthesis coupled electron transport, mitochondrial ATP synthesis coupled electron transport, respiratory electron transport chain, cellular respiration, oxidative phosphorylation, generation of precursor metabolites and energy, energy derivation by oxidation of organic compounds, oxidation reduction

* False Discovery Rate (FDR) P<0.001 with BiNGO.

Table S10. Clusters of highly interconnected genes as identified by MCODE in the network generated with genes containing variants in the WGS cohort. To analyse the physical relationships among genes containing variants, the Cytoscape plug-in Molecular Complex Detection (MCODE, version 2.0.0; http://apps.cytoscape.org/apps/mcode) was used to identify the most important sub-modules of the network map. The criteria of MCODE analysis were degree cut-off = 2, MCODE score > 5, Max depth = 100, node score cut-off = 0.2, and k-score = 2. The Biological Networks Gene Ontology tool (BiNGO, version 3.0.3; http://apps.cytoscape.org/apps/bingo) was used to analyze and visualize the biological processes of identified sub-modules with an FDR corrected P-value<0.001.

Cluster	Score (Density*#Nodes)	Nodes	Edges	Node IDs	GO Biological Processes P<0.001*
1	32	32	491	MRPS28, MRPL1, PTCD3, MRPL21, MRPL13, MRPS30, MRPL54, TSFM2, MRPL4, MRPS23, GFM1, TUFM, MRPS34, MRPS11, MRPS21, MRPS7, GADD45GIP1, MRPL44, CHCHD1, MRPL22, MRRF, MRPS6	translation, cellular macromolecule biosynthetic process, gene expression, macromolecule biosynthetic process, cellular biosynthetic process, biosynthetic process, cellular protein metabolic process, protein metabolic process, cellular macromolecule metabolic process, macromolecule metabolic process, cellular metabolic process, metabolic process, primary metabolic process, DNA damage response, detection of DNA damage, cellular process
2	24	28	323	NDUFB5, NDUFAF2, NDUFB3, MT-ND1, MT-CO1, SDHC, NDUFA6, MT-CYB, MT-CO2, MT-ND6, NDUFC1, PINK1, MT-ND5, NDUFA7, NDUFS2, PRKACA, COX4I1, NDUFAF4, MT-ND4, ECSIT, UQCRB, MT-ND2, NDUFS5, NDUFA10, TMEM126B, MT-ND4L, NDUFC2, NDUFV2	ATP synthesis coupled electron transport, mitochondrial ATP synthesis coupled electron transport, respiratory electron transport chain, cellular respiration, mitochondrial electron transport, NADH to ubiquinone, electron transport chain, oxidative phosphorylation, energy derivation by oxidation of organic compounds, generation of precursor metabolites and energy, phosphorylation, oxidation reduction, phosphorus metabolic process, phosphate metabolic process, transport, localization, establishment of localization, cellular metabolic process, response to oxidative stress, metabolic process, NADH dehydrogenase complex assembly, mitochondrial respiratory chain complex I assembly, mitochondrial respiratory chain complex assembly, response to reactive oxygen species, response to stress
3	9	17	72	DLD, OGDHL, SUCLG2, BCKDHB, BCKDHA, MT-ATP6, DLST, PDHX, IDH3A, ATP5F1D, COX10, IDH3B, ATP5PD, DBT, ACO2, NDUFA4L2, SUCLA2	aerobic respiration, cellular respiration, tricarboxylic acid cycle, generation of precursor metabolites and energy, acetyl-CoA catabolic process, cofactor metabolic process, coenzyme catabolic process, acetyl-CoA metabolic process, energy derivation by oxidation of organic compounds, cofactor catabolic process, coenzyme metabolic process, dicarboxylic acid metabolic process, 2-oxoglutarate metabolic process, carboxylic acid metabolic process, oxoacid metabolic process, organic acid metabolic process, cellular ketone metabolic process, small molecule metabolic process, catabolic process, NADH metabolic process, cellular catabolic process, oxidation reduction, NAD metabolic process, metabolic process, succinyl-CoA metabolic process, nicotinamide nucleotide metabolic process, pyridine

					nucleotide metabolic process, isocitrate metabolic process, cellular metabolic process, oxidoreduction coenzyme metabolic process, cellular nitrogen compound metabolic process, nitrogen compound metabolic process, regulation of acetyl-CoA biosynthetic process from pyruvate, branched chain family amino acid catabolic process, regulation of cofactor metabolic process, regulation of coenzyme metabolic process,
4	9	9	36	WARS2, RARS2, NARS2, LARS2, IARS2, FARS2, DARS2, CARS2, AARS2	tRNA aminoacylation for protein translation, amino acid activation, tRNA aminoacylation, tRNA metabolic process, cellular amino acid metabolic process, ncRNA metabolic process, translation, cellular amine metabolic process, cellular amino acid and derivative metabolic process, amine metabolic process, carboxylic acid metabolic process, oxoacid metabolic process, organic acid metabolic process, cellular ketone metabolic process, RNA metabolic process, cellular macromolecule biosynthetic process, macromolecule biosynthetic process, gene expression, small molecule metabolic process, nucleic acid metabolic process, cellular biosynthetic process, nucleobase, nucleoside, nucleotide and nucleic acid metabolic process, biosynthetic process, cellular nitrogen compound metabolic process, cellular protein metabolic process, nitrogen compound metabolic process, protein metabolic process, aspartyl-tRNA aminoacylation, cellular macromolecule metabolic process, macromolecule metabolic process, cellular metabolic process, primary metabolic process, metabolic process,

* False Discovery Rate (FDR) P<0.0001 with BiNGO.

Figure S1. RNA duplex formation predicted by RNAhybrid of hsa-miR-4639-5p and **A)** human PARK7/DJ-1 3′UTR wild type sequence **B)** human PARK7/DJ-1 3′UTR mutated sequence.

a)

```
dataset: 1
Target: PARK7|NM_001123377|3'UTR
length: 42
MiRNA: hsa-miR-4639-5p
length: 22

mfe: -18.3 kcal/mol
p-value: 1.000000e+00

Position: 1
target 5' A   A AG            A 3'
           UAA C  GGC  AUUUAGCAA
           GUU G  UCG  UGAAUCGUU
miRNA  3' A   A AG   GA          5'

plot as png,jpeg or ps (in a new window)
```

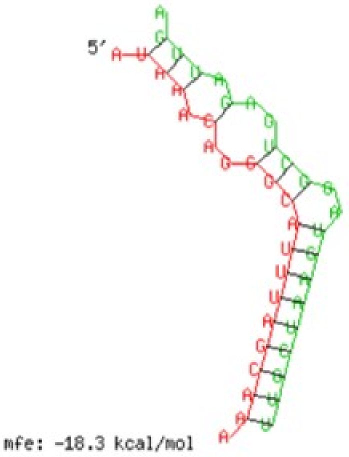

b)

```
dataset: 1
Target: PARK7|NM_001123377|3'UTR
length: 42
MiRNA: hsa-miR-4639-5p
length: 22

mfe: -18.4 kcal/mol
p-value: 1.000000e+00

Position: 1
target 5' A   A     G           A 3'
           UAA C CGG C AUUUAGCAA
           GUU G GUC G UGAAUCGUU
miRNA  3' A   A A   G A          5'

plot as png,jpeg or ps (in a new window)
```

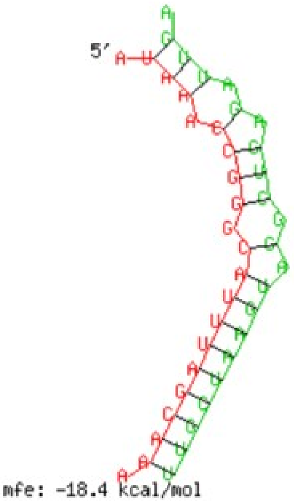

Figure S2. mtDNA variant counts and proportions in WES dataset . (a) Number of the Homoplasmic (HOM) and heteroplasmic (HET) variants in ALS and CNTR tissues. (b) Proportion (%) of variants in the Control and Coding regions of the mitochondrial genome in ALS and control subjects. (c) Number of variants in Hyper-variable regions of the Control Region. (d) Proportion (%) of Homoplasmic (HOM) and heteroplasmic (HET) variants in the Control Region in ALS and control subjects. (e) Total number of variants in mitochondrially encoded protein-coding genes observed in ALS and CNTR tissues. (f) Proportion (%) of Homoplasmic (HOM) and heteroplasmic (HET) variants in mitochondrially encoded protein coding genes in ALS and CNTR tissues. (g) Total number of variants in tRNA and rRNA genes in ALS and CNTR samples. (h) Proportion(%) of Homoplasmic (HOM) and heteroplasmic (HET) variants in tRNA and rRNA genes in ALS and CNTR tissues. (i) Proportion (%) of variants with HIGH, MODERATE, LOW and MODIFIER predicted effect in ALS and CNTR samples. (j) Proportion (%) of Heteroplasmic (HET) variants with HIGH, MODERATE, LOW and MODIFIER predicted effect in ALS and CNTR samples. P-values designated with 'P' are derived from the Fisher's exact test, whereas 'p' indicates values obtained from chi-square test.

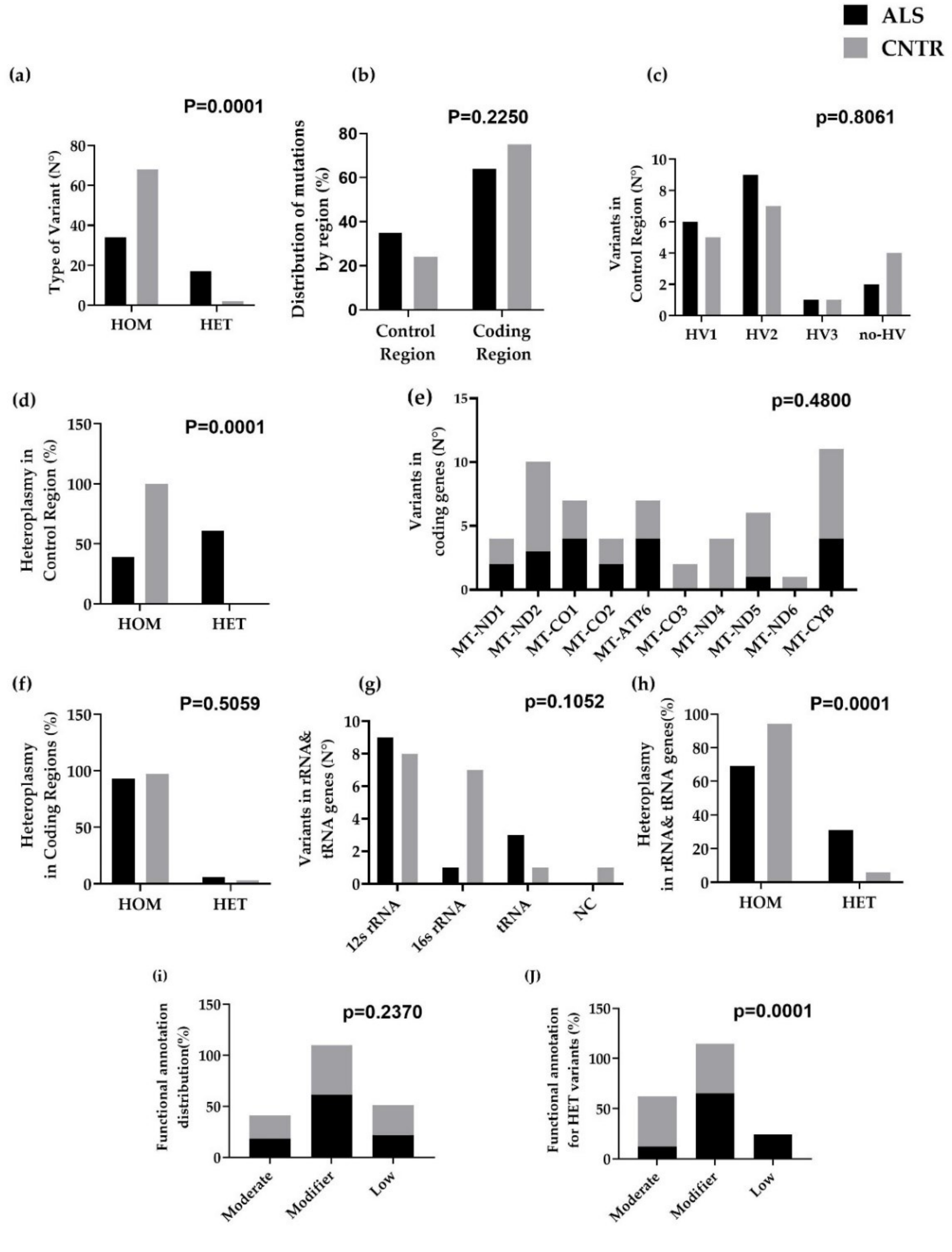

Figure S3. Structural or thermodynamic differences between wild type Cambridge Reference Sequence (rCRS) and mutated forms in ALS of the secondary structure C. First 42 nt of the ETAS2 region sequence as identified by Sbisà et al., which includes the secondary structure C as described by Pereira et al. Secondary structure and energy information are depicted along with a graphical drawing and Dot bracket representation of **a)** wild type rCRS **b)**ALS_1 m.16298T>C mutated sequence **c)** ALS_2 m.16304T>C; m.16311T>C mutated sequence **d)** ALS_3 m.16304T>C; m.16311T>C mutated sequence.

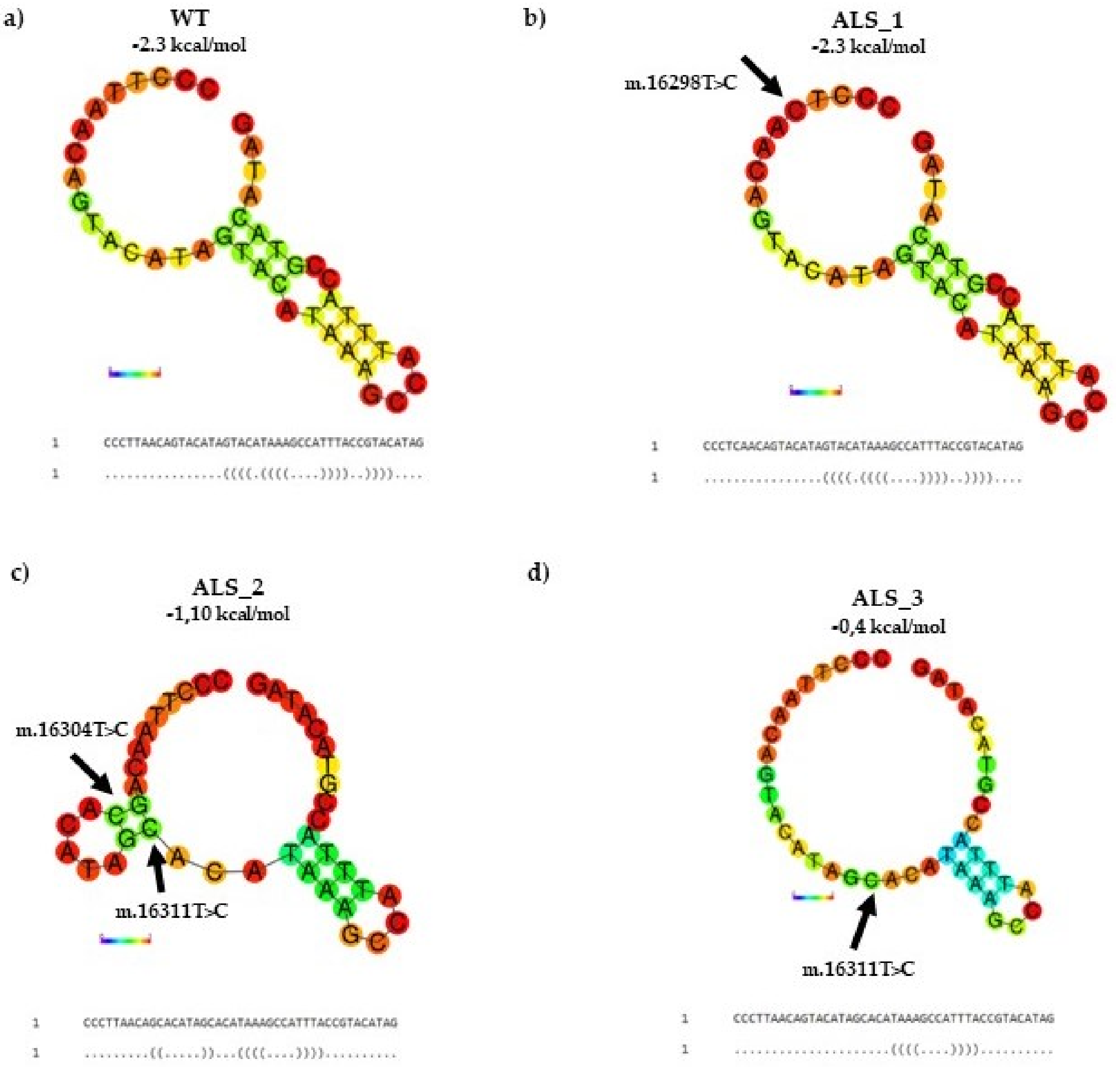

Figure S4. Structural or thermodynamic differences of variant sites in mtDNA that passed the filtering and prioritization steps in the WES dataset. Structural or thermodynamic differences between wild type Cambridge Reference Sequence (rCRS) and mutated forms of secondary structures as described by Pereira et al. Secondary structure and energy information are depicted along with a graphical drawing and dot bracket representation of **a)** wild type K secondary structure (nt. 181-226); **b)** ALS_1 m.195T>C, m198C>T mutated sequence **c)** wild type J secondary structure with 4 added nucleotides (nt.116-154); **d)** ALS_2 m.16304T>C; m.153T>C mutated sequence. The major H-strand replication initiation site (O_H).

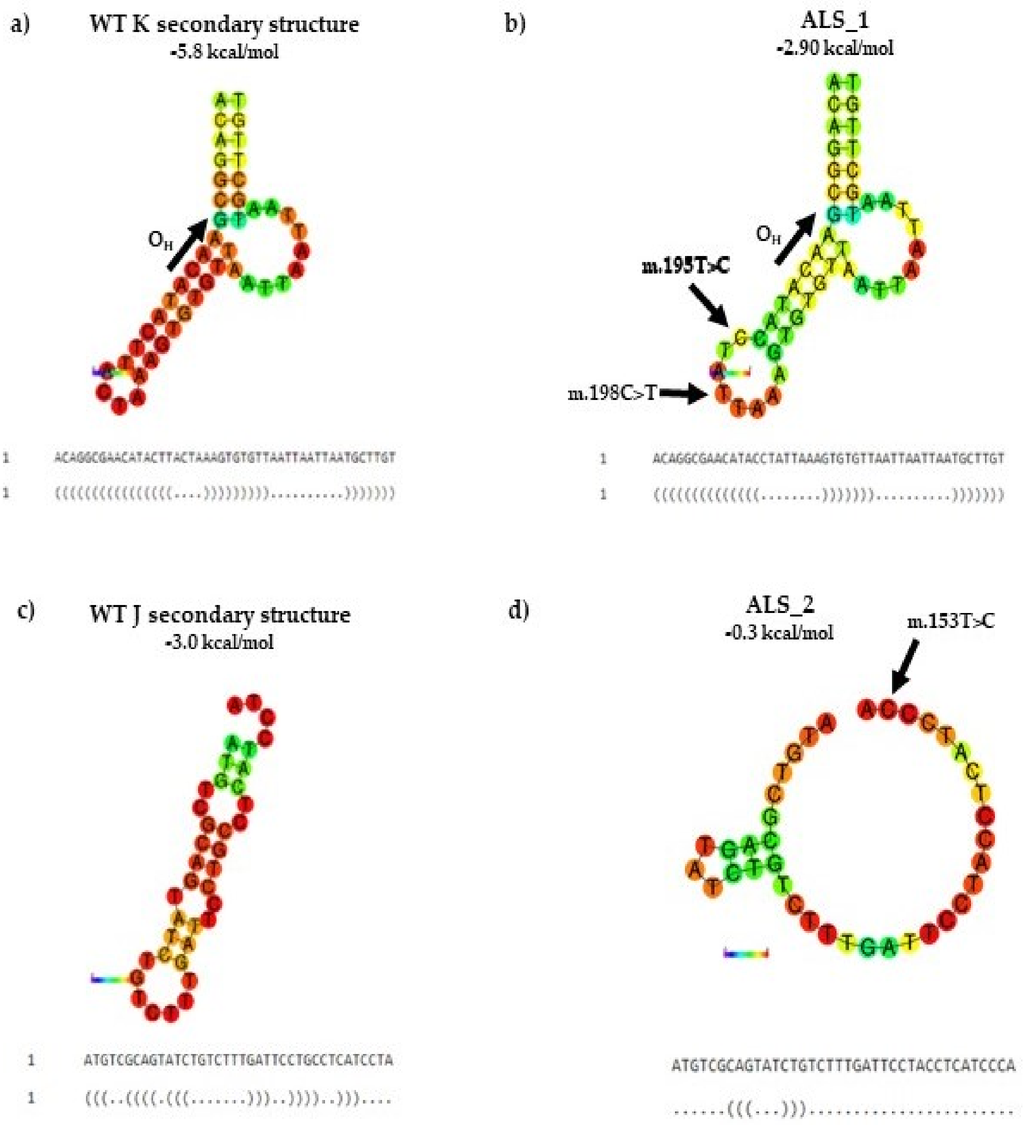

Figure S5. mtDNA variant counts and proportions in WGS dataset. (a) Number of homoplasmic (HOM) and heteroplasmic (HET) variants between ALS and CNTR tissues. (b) Proportion (%) of variants in the Control and Coding regions of the mitochondrial genome in ALS and control subjects (c) Number of variants in Hyper-variable regions of the Control Region. (d) Proportion (%) of Homoplasmic (HOM) and heteroplasmic (HET) variants in the Control Region in ALS and control subjects. (e) Total number of variants in mitochondrially encoded protein-coding genes observed in ALS and CNTR tissues. (f) Proportion (%) of Homoplasmic (HOM) and heteroplasmic (HET) variants in mitochondrially encoded protein coding genes in ALS and CNTR tissues. (g) Total number of variants in tRNA and rRNA genes in ALS and CNTR samples. (h) Proportion (%) of Homoplasmic (HOM) and heteroplasmic (HET) variants in tRNA and rRNA genes in ALS and CNTR tissues. (i) Proportion (%) of variants with HIGH, MODERATE, LOW and MODIFIER predicted effect in ALS and CNTR tissues. (j) Proportion (%) of Homoplasmic (HOM) variants with HIGH, MODERATE, LOW and MODIFIER predicted effect in ALS and CNTR samples.

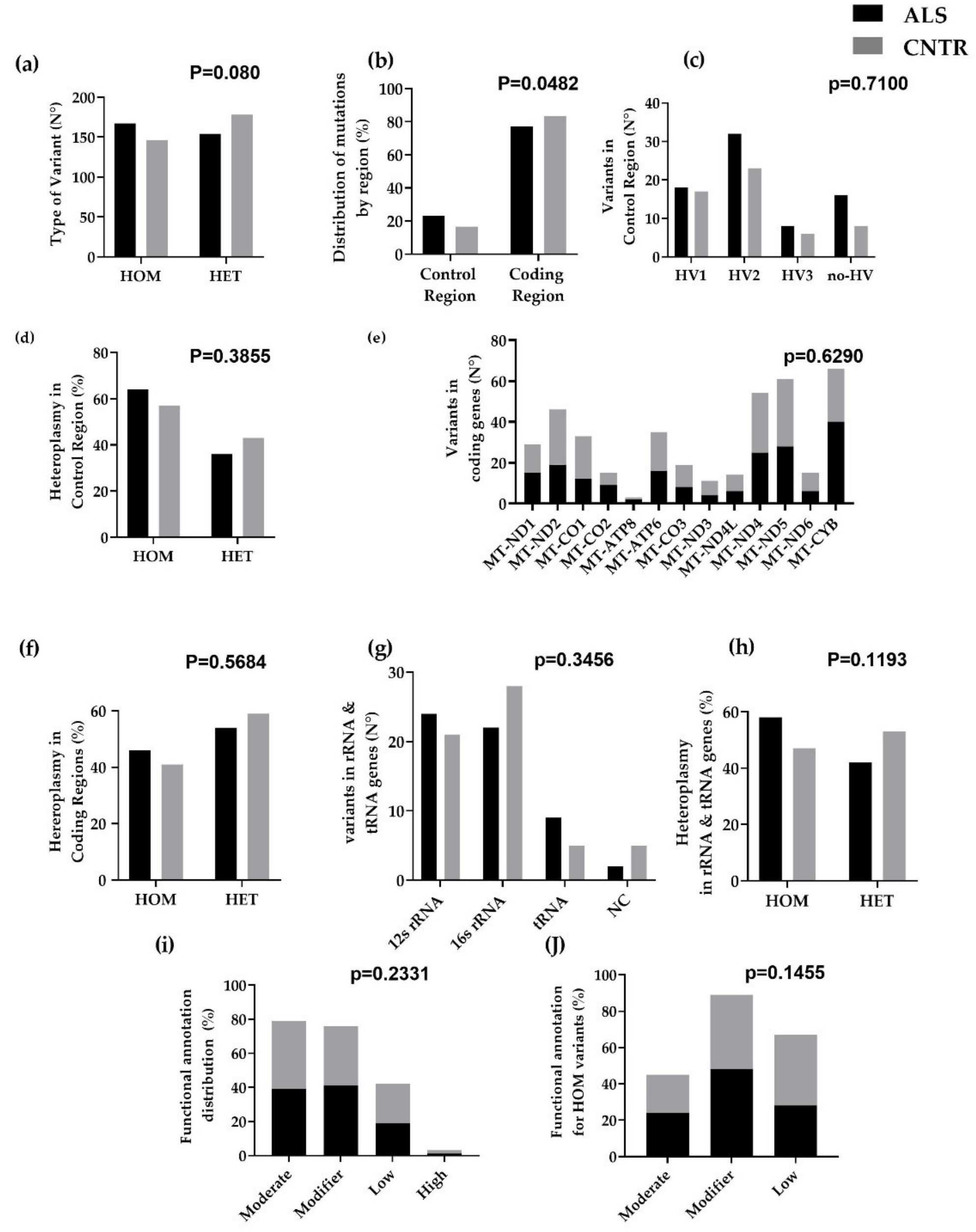

Figure S6. Structural or thermodynamic differences of variant sites in mtDNA that passed the filtering and prioritization steps in the WGS dataset. Structural or thermodynamic differences between wild type Cambridge Reference Sequence (rCRS) and mutated forms of secondary structures as described by Pereira et al. Secondary structure and energy information are depicted along with a graphical drawing and dot bracket representation of **a)** wild type J secondary structure (nt 116-149); **b)** ALS_3_536 m.143G>A mutated sequence **c)** wild type K secondary structure (nt 181-226); **d)** ALS1_497 m.183A>G mutated sequence **e)** ALS7_5610 m.204T>C mutated sequence **f)** wild type L secondary structure (nt 249-279) with 4 added nt (nt 245-279); **g)** ALS6_5609 with a m.247G>T mutated sequence; **h)** wild type A secondary structure (nt 16028-16120); **I)** AL2_503 m.16084G>A mutated sequence; **j)** WT F secondary structure (nt. 16481-16513) **k)** AL2_503 m.16496G>A mutated sequence. The major H-strand replication initiation site (OH).

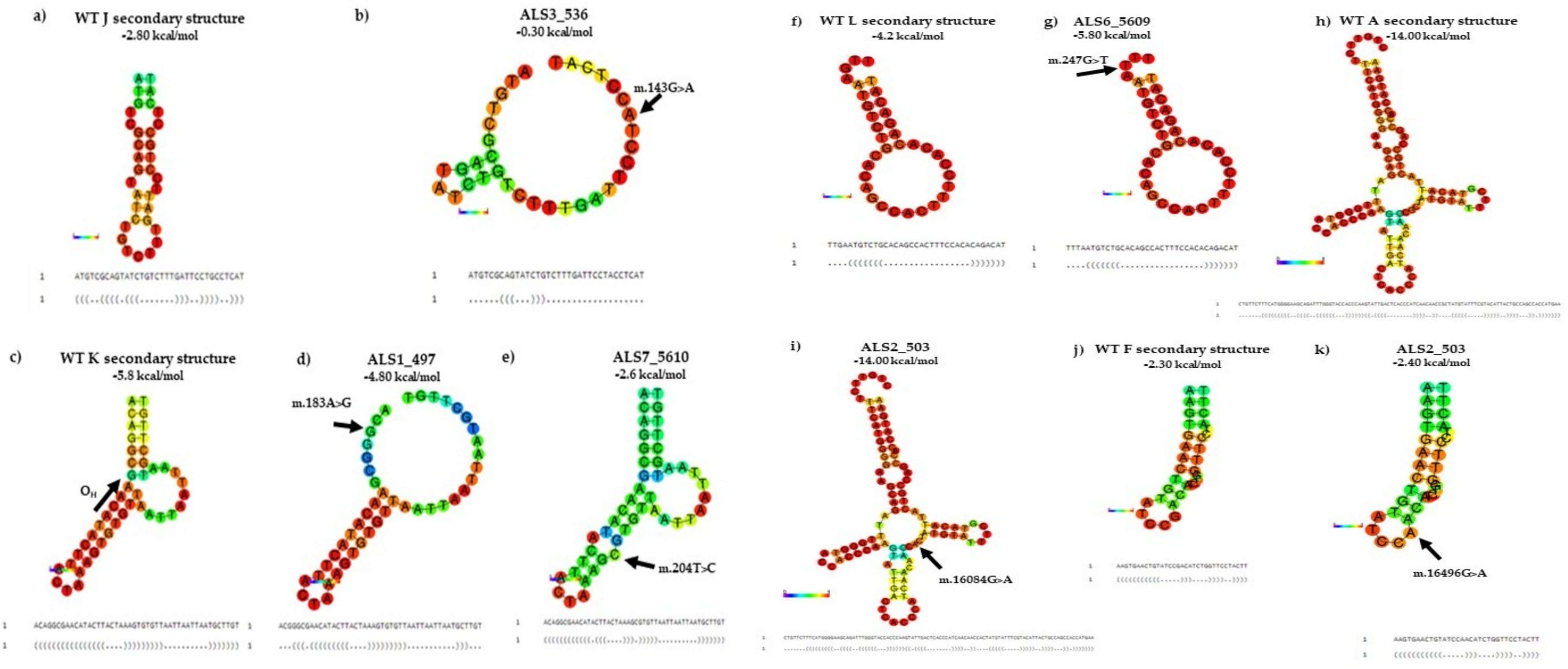

Supplement: Supplementary file 1 [file biomolecules-14-00411-s001.zip › biomolecules-2879041-supplementary.pdf]
